# State-of-the-art boron clusters for boron neutron-capture therapy

**DOI:** 10.7150/thno.123376

**Published:** 2026-01-01

**Authors:** Weiyao Wang, Enze Zhang, Jiaojiao Shan, Min Zhang, Renwei Cai, Runze Li, Lulian Pang, Baosheng Li, Dejin Zang

**Affiliations:** 1 School of Pharmaceutical Sciences & Institute of Materia Medica, National Key Laboratory of Advanced Drug Delivery System, Key Laboratory for Biotechnology Drugs of National Health Commission (Shandong Academy of Medical Sciences), Key Lab for Rare & Uncommon Diseases of Shandong Province, Shandong First Medical University & Shandong Academy of Medical Sciences, Jinan, Shandong, 250117, China.; 2 Department of Radiation Oncology, Shandong Cancer Hospital and Institute, Shandong First Medical University and Shandong Academy of Medical Sciences, Jinan, Shandong, 250117, China.; 3 Qingdao Medical College of Qingdao University, No. 1 Ningde Road, Haoyuan, Qingdao University, Qingdao, 266071, China.; 4 The Affiliated Hospital of Shandong University of Traditional Chinese Medicine, Jinan, 250114, China.

**Keywords:** BNCT, Boron clusters, Targeted drugs delivery, Accelerator devices, Anti-cancer mechanism

## Abstract

Boron neutron-capture therapy (BNCT) is a highly precise, cell-level cancer radiotherapy. It exploits the neutron-capture reaction that occurs when low-energy thermal neutrons are absorbed by a boron-10 atom, triggering a nuclear fission reaction that releases high-energy particles to selectively kill cancer cells. BNCT is at the forefront of cancer treatment. Presently, only sodium mercaptoundecahydro-closo-dodecaborate and boron borylphenylalanine (BPA) have been approved as boron drugs for clinical trials by the Food and Drug Administration. However, these drugs still suffer from shortcomings, such as poor targeting, low concentration in cancer cells, a short residence time, and low overall applicability. Conversely, boron clusters are three-dimensional polyhedral structures composed of carbon, boron, and hydrogen atoms. Owing to their excellent stability and unique three-dimensional shape, they are ideal candidates for next-generation boron drugs. These unique features make boron clusters an ideal model for correlating macroscopic properties with the microstructures of substances, providing a valuable framework for the rational design of next-generation boron drugs. Thus, from an interdisciplinary perspective, this review summarizes new strategies for constructing boron clusters, including multi-level structures. We describe key chemical strategies for their functionalization for clinical applications, reveal the multi-scenario applications of their line-functionalized derivatives, and highlight their cross-disciplinary value in precision synthesis, biomedicine, and advanced materials, all with a focus on elucidating the structure-function relationship in boron clusters. Additionally, we explored the latest advancements in the visual evaluation of BNCT, its anticancer mechanism, and exclusive neutron accelerator devices. In summary, the development of novel boron drugs based on functional boron clusters is a prerequisite to resolving the key technical issues in the research and development of new BNCT agents. This review provides insights into the design of new BNCT drugs, as well as related supporting equipment and treatment options, from the perspectives of medicinal chemistry and clinical applications.

## 1. Introduction

Boron neutron-capture therapy (BNCT), as an emerging cancer treatment, has demonstrated numerous unique advantages in the field of precision medicine. It originated in the 1930s, beginning with the 1932 discovery of the neutron by British physicist, James Chadwick 1, who first bombarded beryllium with alpha particles before using the resulting new rays to bombard hydrogen, nitrogen, and other elements. Interestingly, the hydrogen and nitrogen nuclei exhibited a recoil phenomenon. Thus, by measuring the speed of these nuclei, Chadwick deduced the mass of this new particle and demonstrated its neutral nature with a mass nearly identical to that of a proton. This new particle was named the neutron. Three years later, in 1935, Taylor and Goldhaber described thermal-neutron capture by the boron-10 (^10^B) nucleus, ultimately laying the theoretical foundation for BNCT. In 1936, Lohr proposed the use of this reaction (thermal-neutron capture) to treat cancer tumors, marking the first time BNCT was envisioned for cancer treatment 2. The early clinical exploration of BNCT began in 1951, with a collaboration between William Herbert Sweet, a neurosurgeon at the Massachusetts Institute of Technology, and Brookhaven National Laboratory. They initiated the first human BNCT study on malignant gliomas, opening a new phase of clinical application of the therapy 3, 4. During the experiments, the neurosurgeon, Sweet, used inorganic boric acid (sodium tetraborate) as the boron-containing drug, and subsequently irradiated the tumor sites with neutrons produced by a nuclear reactor. However, none of the patients who participated in the experiment survived more than one year post-treatment. Additionally, severe adverse reactions were observed during the treatment, including radioactive dermatosis, cerebral edema, intractable shock, and cerebral necrosis. These side effects were attributed to two main factors. First, the neutron source at that time produced and facilitated limited neutron energy and penetration, respectively, complicating the penetration of deep-seated tumor tissues in the brain and significantly reducing the therapeutic effect, which made the implementation of effective neutron captures challenging in such deep regions. Second, the selected inorganic boric acid exhibited poor selectivity, preventing its selective aggregation in the tumor cells. This led to the non-specific uptake of a certain amount of boron by normal brain tissues, causing a certain degree of damage during neutron irradiation. These adverse effects led to the abrupt suspension of the clinical trials of BNCT in the United States in 1961. As the interest in the topic faded within the US, the international level became more active. In Japan, Hiroshi Hatanaka played a pioneering role in developing the clinical trials of BNCT, leading a team that began treating malignant brain tumors in 1968. The team conducted clinic studies between 1966 and 1993, in which they treated approximately 120 patients with high-grade gliomas. Using a new boron compound, sodium mercaptoundecahydro-closo-dodecaborate (BSH), in combination with surgery and BNCT, the team achieved some successes. A data comparison revealed that the BNCT group had a 5-year survival rate of 19%, a significant improvement over the 5% rate in the standard treatment group; additionally, the BNCT group for patients with tumors at a depth of 6 cm or less from the skin surface of the head had a 5-year survival rate that was as high as 58% 5, 6. These clinical trials validated the potential of BNCT, providing valuable data and references for subsequent research and application, and establishing its clinical foundation as a cancer-treatment procedure. In 1987, Mishima in Japan first reported the use of boron phenylalanine (BPA) as a boron compound for BNCT in the treatment of malignant melanoma, and this accounted for the first significant applications of the BNCT technology in extracranial treatment 7, 8. The selective uptake of BPA, mediated by the L-type amino acid transporter (LAT1), enhanced and suppressed ^10^B uptake by tumor cells and normal tissues, respectively, thus improving the precision and safety of the treatment 9. Ever since, BNCT has experienced a resurgence, with clinical trials being conducted in the US and Japan. After decades of research inactivity, BNCT clinical trials are gradually increasing, offering new development opportunities. For example, a prospective, single-center phase I/II clinical study of recurrent head and neck tumors was conducted in Finland. The study included 12 patients with recurrent and inoperable locally advanced head and neck tumors who had received photon radiotherapy. These patients were treated with an intravenous infusion of the boron-containing drug (400 mg/kg), and the most common acute side effects were mucositis, fatigue, and localized pain. In 2012, Kankaanranta *et al.* further analyzed the safety and efficacy of this phase I/II clinical study, revealing that BNCT was an effective and acceptably well-tolerated treatment for locally recurrent, inoperable, and previously treated head and neck cancers 10. The widespread utilization of BPA and BSH marked the second-phase entry of BNCT, with improved efficacy for the treatment of recurrent head and neck tumors, gliomas, and malignant melanomas. In the 1990s, the modern era of BNCT was marked by the introduction of the second-generation compounds, levoborate-BPA (L-BPA) and BSH. At that time, neutrons were still supplied by nuclear reactors. For BNCT to achieve broader and more effective clinical applications, several issues must be urgently addressed. These include improving the targeting and boron loading of new boron-based drugs, achieving technological breakthroughs in neutron-source equipment, and expanding the range of treatable cancers and personalized therapies. These factors collectively determine whether BNCT can become a powerful tool in cancer treatment 11. As a precision tumor therapy, the clinical efficacy of BNCT depends highly on the targeting efficiency of the boron-based drug, as well as the boron loading. Consistent in-depth exploratory studies worldwide have revealed that an ideal boron-based drug exhibits the following characteristics: a) high uptake by tumor cells, with a ^10^B concentration of 20-50 μg/g in tumor tissues; b) high tumor specificity, with a tumor-to-normal tissue concentration ratio (T/N) and tumor-to-blood concentration ratio (T/B) of more than 3; c) intrinsic low toxicity, good water solubility, and no harm to normal tissues; and d) good pharmacokinetics (possibility of being rapidly cleared from blood and normal tissues while being retained for a long time in tumors) 12-16. It can also be absorbed by anaerobic cells to cover different cellular states within the tumor. Currently, conventionally utilized boron drugs for BNCT include 4-dihydroxyboronyl-L-type phenylalanine (BPA) and BSH, although they suffer from significant deficiencies, such as tumor targeting and boron loading. First, the solubility limitations of BPA and its inability to carry boron atoms mean that multiple BPA injections are required to maintain the required boron concentration for the treatment, and this increases the burden on the patient and may also result in drug accumulation in normal tissues, thereby increasing the risk of potential side effects. Second, the T/N values of BPA and BSH 17 are only 3-5, which are significantly lower than the ideal. Conventional BNCT mainly relies on nuclear reactors to produce the neutron beams for the treatment. However, the limitations of nuclear reactor neutron sources have hindered the widespread adoption of BNCT. After 2014, three Japanese industries and an American neutron-processing company collaborated to develop an accelerator-based BNCT (AB-BNCT) neutron source. This source, which can be installed in hospitals, produces a shallow-forming thermal-neutron beam. The AB-BNCT neutron source has several advantages over nuclear reactor neutron sources, including on-demand shutdown, negligible permanent radioactive residues, simpler licensing procedures, easier installation and maintenance, lower costs. Their design also aligns with the established experience with gas pedals in hospital radiotherapy departments and provides a higher-quality neutron source. Consequently, several clinical AB-BNCT development programs are underway, with a leading Japanese company developing the most advanced cyclotron 18. Despite significant advances in gas-pedal neutron source technology, its global penetration remains limited. Currently, only a few countries and regions have advanced gas-pedal BNCT facilities that have undergone relevant clinical trials. For example, Kyoto University in Japan and Hongai Hospital in Xiamen are typical representatives of facilities that have achieved the clinical application of the gas-pedal BNCT equipment 14. These clinical applications revealed that gas-pedal neutron sources exhibit good application prospects in BNCT, although further technological breakthroughs and equipment optimization are required to improve their performance and reliability. Most importantly, the application scope of BNCT in tumor treatment is still relatively small, significantly restricting its role in clinical applications 19. The extant clinical trials and studies revealed that BNCT is mainly effective in the treatment of a few specific tumor types, such as malignant gliomas 19-22, cutaneous melanomas 23, 24, and recurrent head and neck tumors 25. While BNCT has been applied to the treatment of malignant gliomas, melanomas, and recurrent head and neck tumors, its overall clinical scope remains relatively limited. However, with the development of new boron-carrying drugs, technological innovation in gas-pedal neutron sources, and breakthroughs in the design of key technologies, such as the design of individualized treatment plans, the scope of its clinical research has gradually expanded to include the treatment of multiple types of solid tumors. Preliminary clinical data confirm that BNCT has demonstrated significant dual-targeted radiobiological effects and clinical potential in treating tumors, such as hepatocellular carcinoma (HCC) 26, breast cancer (BC) 27, and other malignant tumors. With the continuous breakthroughs in key technologies and the accumulation of evidence-based medical evidence, BNCT is projected to overcome the existing limitations, gradually realize pan-cancer precision treatment, and provide innovative solutions for the comprehensive management of malignant tumors.

Further, the development of boron carriers has always been considered a key driver of the clinical translation of BNCT, following its emergence in the mid-20^th^ century. To date, the first generation of BNCT drugs includes boric acid and its derivatives. The second generation of conventional boron-based drugs, represented by the organic boron compounds, BPA and BSH, has formed the fundamental framework for clinical applications. BPA is an L-phenylalanine-derived boronic acid, which is recognized and absorbed by tumor cells via their highly expressed LAT1. BPA exhibits a T/N of more than 3:1 in tumors, such as glioma and head and neck cancer. In practice, limitations such as low solubility and rapid metabolism have necessitated the development of improved dosage forms. The complexation of BPA with fructose and sorbitol has been reported to significantly ameliorate these limitations. In contrast to BPA, BSH has been employed in the early stages of research, mainly in brain tumors, owing to its excellent capability of crossing the pathologically permeable blood-brain barrier (BBB), its high boron concentration, and its excellent water solubility 28, 29. While the caged molecular structure of BSH is advantageous for its high boron-loading capacity, its delivery mechanism is limited by its sole reliance on passive diffusion. This results in inadequate tumor targeting and an inability to localize effectively within cells. Moreover, BSH is susceptible to nephrotoxicity owing to its accumulation in the kidneys. Inorganic boron is represented by carbon boranes, and the studies on boron agents have advanced over time and experience. Ongoing studies on the cellular and subcellular localization of promising boron agents have stimulated a greater role for modern cell biology in the design of these agents and their delivery vehicles. Third-generation boron compounds have significantly improved the precision and efficacy of BNCT through molecular targeting, nanotechnology, and multifunctional designs. They are projected to propel it into becoming a mainstream choice for treating solid tumors 30-35.

Boron clusters (e.g., dodecaborane) are ideal BNCT carriers owing to their unique chemical structures. First, their high boron density significantly exceeds those of traditional boron carriers (e.g., BPA), i.e., a single molecule of a boron cluster contains 9-12 boron atoms, facilitating high intra-tumor boron concentration at low administration doses and reducing systemic toxicity 36. Second, their closed-cage structures equip them with excellent chemical stability, enabling them to maintain their integrity under complex in vivo conditions (e.g., pH fluctuations and enzymatic reactions) and ensuring effective delivery to the target site. Furthermore, by enriching the ¹⁰B isotope, boron clusters can optimize the neutron-capture reaction. This process releases high-energy α-particles (^4^He^2+^) and lithium nuclei (^7^Li^3+^), which can precisely target and destroy tumor cells with minimized collateral damage to surrounding normal tissues 37, 38. Collectively, these attributes underscore the central role of boron clusters in BNCT and position them as ideal platforms for functional modifications. The innovation of third-generation boron compounds mainly focuses on boron clusters, using targeted delivery systems (e.g., functionalized nanocarriers and peptide couplings) to significantly enhance the selective accumulation of boron compounds in tumor tissues and solving the core limitations of traditional small-molecule boron carriers (e.g., BPA and BSH), such as low targeting, poor permeability, and unstable metabolism 31, 39, 40.

**Scheme 1:**
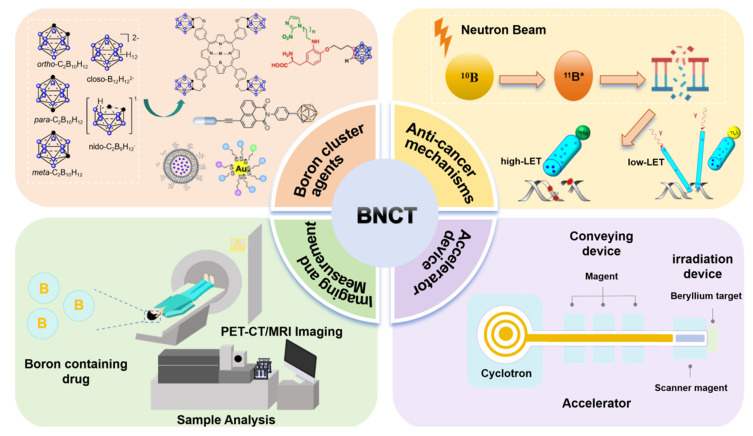
The system demonstrates the core elements of BNCT, including the typical boron cluster structure of boron drugs, the cancer-fighting mechanisms triggered by neutron capture, the accelerator-based equipment system, and the techniques for boron detection and imaging analysis.

In this review, we explore the development trend of BNCT from four key perspectives: boron-cluster agents, anticancer mechanisms, visual assessment, and accelerators (Scheme 1). We systematically elucidate the development history, core technologies, and future directions of BNCTs, as well as explore their potential for expansion into pan-cancer types, combination therapies, and precision clinical applications.

## 2. Boron clusters as potential agents for boron neutron-capture therapy

**Figure 1:**
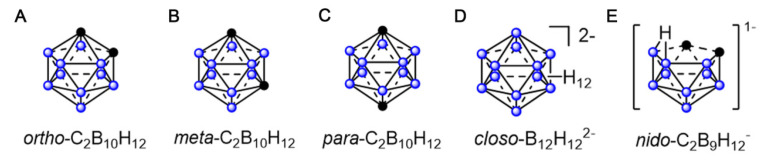
** (A)** rtho-(1,2-C_2_B_10_H_12_) carborane. **(B)** meta-(1,7-C_2_B_10_H_12_) carborane. **(C)** para- (1,12-C_2_B_10_H_12_) carborane. **(D)** closo**-**B_12_H_12_^2-^. **(E)** *nido*-C_2_B_9_H_12_^-^.

Boron clusters are polyhedral structures comprising boron, carbon, and hydrogen atoms. Their boron-rich properties and unique three-dimensional (3D) structures, good catabolic stability, and low-toxicity position them as promising candidates for driving BNCT 41. Polyhedral boron clusters, such as boranes, carboranes, and other related heteroboranes, are mainly categorized into four structural types: closo, nido, arachno, and hypho. The closo structure is a complete polyhedron. Typically exhibiting the closo- prefix, this structure is a complete polyhedron. The nido-, arachno-, and hypho- terminologies are used when one, two, or three vertices are missing from the structure, respectively 41. The physicochemical properties of carbaboranes differ significantly with their sizes and shapes. Dicarba-closo-dodecaboranes (C_2_B_10_H_12_), simply referred to as closo-carboranes, are the most stable and widely deployed compounds in boron clusters. They comprise two carbon atoms and 10 boron atoms that are arranged at each vertex of an icosahedron. Depending on the positions of their carbon atoms, closo-carboranes mainly exist as (1)ortho-1,2-C_2_B_10_H_12_, (2)meta-1,7-C_2_B_10_H_12_, and (3)para-1,12-C_2_B_10_H_12_ (Figures 1A-C) 39, 42-47. Other boron-containing isomeric carbaboranes have also exhibited promise as tumor-targeted boron-delivery agents, representing significant progress in the field 48-50(Figures 1D-E). Polyhedral boranes are attractive for medical and pharmacological applications owing to two key properties: first, their low chemical reactivity and decomposition resistance in biological systems make them relatively non-toxic; second, they can be readily modified for specific purposes. Moreover, current boron-cluster design strategies are mainly based on achieving their targeted accumulation at tumor sites.

### 2.1 Boron-cluster-based small-molecule compounds

This section is more detailed and will focus on classical small-molecule carriers and delivery strategies using peptide-boron cluster conjugates, with a primary emphasis on amino acids, porphyrins, and peptides 40, 51.

#### 2.1.1 Boron-cluster-based amino acid carriers

Amino acids are fundamental substances in the human body, serving as a basis for metabolism. They also act as a crucial nutrient for tumor cells, which require a large number of amino acids for their proliferation. Additionally, many solid tumors, such as glioblastoma, melanoma, and glioma, highly express LAT1, which is prominently present in the BBB and distributed on the luminal and abluminal sides of brain capillary endothelial and parenchymal cells 52. It transports naturally occurring substrates and substrate-associated compounds. These properties have enabled the targeted delivery of amino acid-derived boron carriers, which have emerged as therapeutic targets for brain tumor therapy. The study of amino acid carriers began with BPA, which was readily recognized and absorbed by tumor cells via the highly expressed LAT1. This inspired the design of boron drugs and has inspired the synthesis of numerous BPA derivatives.

**Figure 2:**
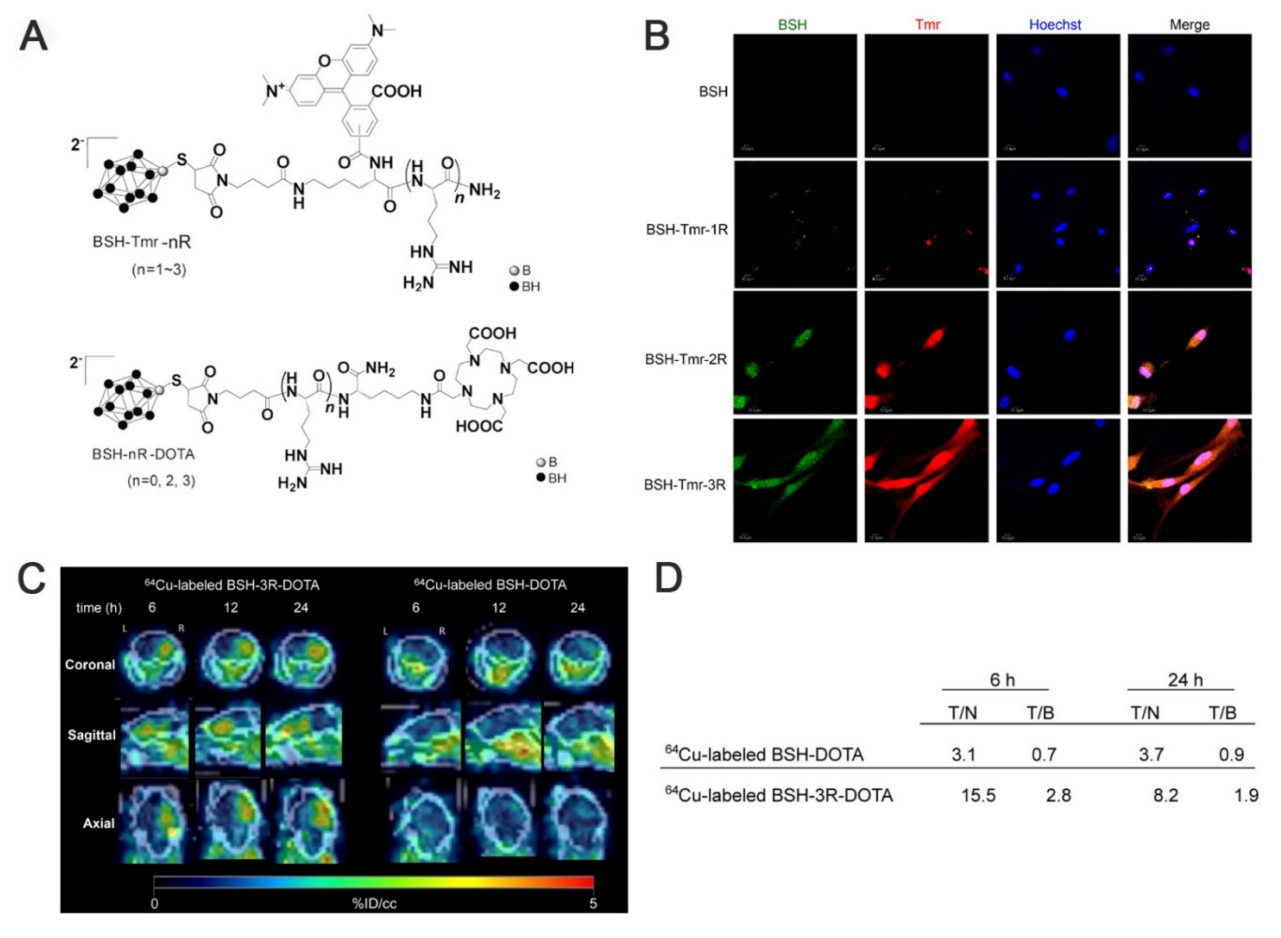
**(A)** Chemical structure of BSH peptide fused with either tetramethylrhodamine (Tmr) or 1,4,7,10-tetraazacyclododecane-1,4,7,10-tetraacetic acid (DOTA). **(B)** Confocal imaging of U87ΔEGFR cells. **(C)** PET images of the brain of U87ΔEGFR tumor-bearing mice at 6 h, 12 h, and 24 h post-injection of ^64^Cu-labeled BSH-3R-DOTA and ^64^Cu-labeled BSH-DOTA. **(D)** The radioisotope ratio of tumor to normal brain (T/N) and tumor to blood (T/B) measured by the gamma counter in each time course, 6 h and 24 h, with ^64^Cu-labeled BSH-3R-DOTA and ^64^Cu-labeled BSH-DOTA. Reproduced with permission from Ref. 54. Copyright 2015, Elsevier.

**Figure 3:**
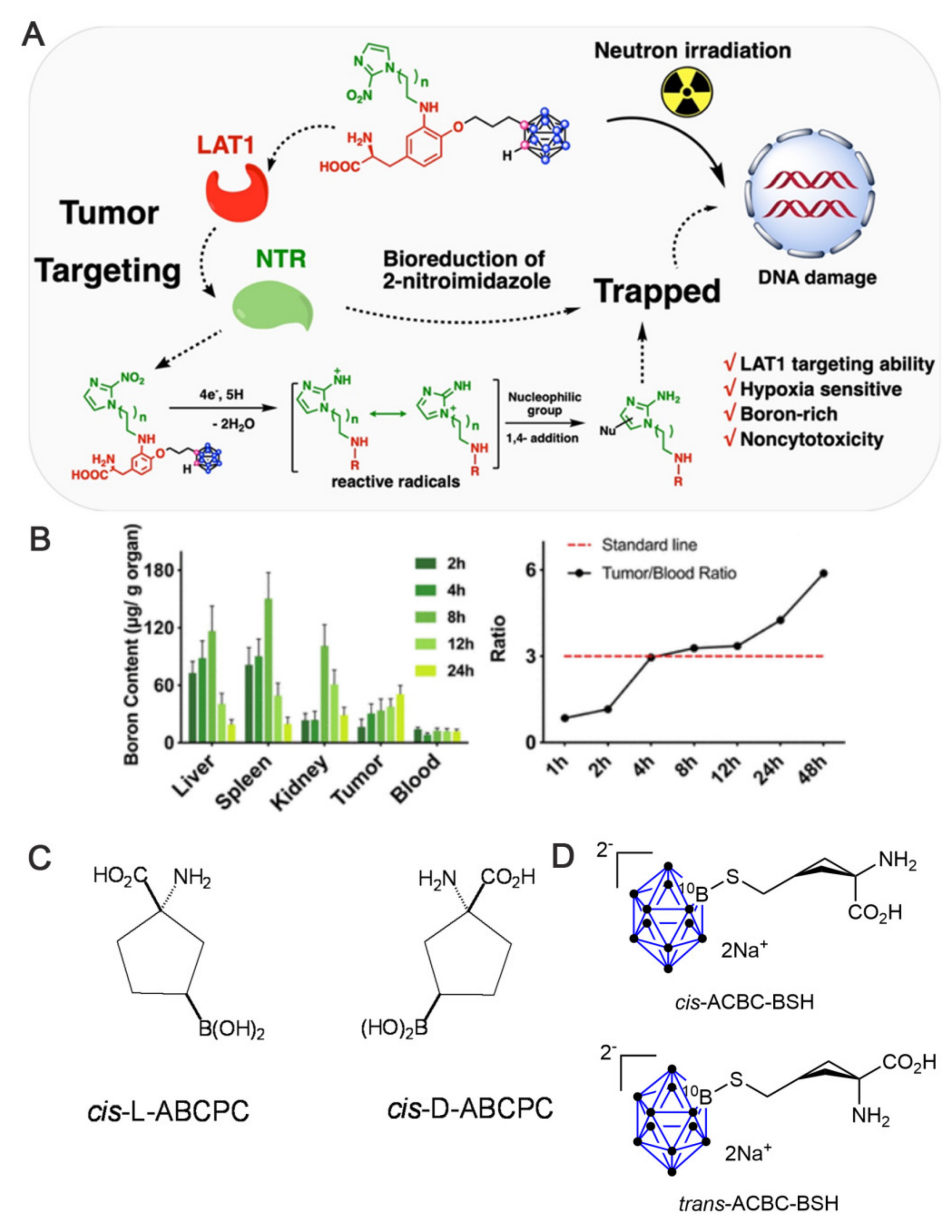
** (A)** Schematic representation of the BNCT principle for nitroimidazole-carborane-modified phenylalanine derivatives. Reproduced with permission from Ref. 12. Copyright 2019, ACS. **(B)** Boron content in major organs and T/B boron concentration ratio in mice. Reproduced with permission from Ref. 12. Copyright 2019, ACS. **(C)** Chemical Structures of L- and D- enantiomers of cis-1 amino-3-borono-cyclopentanecarboxylic acid (cis-ABCPC). Reproduced with permission from Ref. 56. Copyright 2013, PLOS ONE. **(D)** Structures of cis-and trans-ACBC-BSH. Reproduced with permission from Ref. 57. Copyright 2014, Springer Nature.

As a small-molecule boron cluster, BSH has garnered interest among researchers for its more abundant ^10^B content and excellent water solubility despite its poor absorption by cellular tissues 19, 53. The Iguchi team 54, after transducing BSH into the cells using polyarginine, combined it with a short arginine via ^64^Cu-labeled imaging to design a BSH peptide-boron formulation (BSH-3R), a compound that penetrates tumor cells, exhibits excellent cell membrane permeability, and displays higher boron concentrations in vitro and in vivo compared with conventional BSH. Immunohistochemistry (IHC) confirmed the stable presence and precise localization of BSH-3R in the cytoplasm and nucleus. Additionally, the inductively coupled plasma (ICP) data obtained after 24 h of injecting the boron drug revealed that the T/N and T/B of ^10^B were 8.2 and 1.9, respectively. The experimental results demonstrated that BSH-3R could be utilized as an effective BNCT drug (Figure 2). Li *et al.* 12 developed a nitroimidazole-carbon-boron-modified phenylalanine derivative exhibiting a dual-targeting mechanism. This compound simultaneously targets the overexpressed LAT1 and the response mechanism of the hypoxic microenvironment, which elevates boron absorption in melanoma cells by up to 70-fold compared with BPA in vitro. In vivo, the compound demonstrated a T/B of 5.88 and a significantly higher lethal dose, 50% than BSH. Overall, this design skillfully circumvents the shortcomings of traditional boron carriers (e.g., the low boron content of BPA and zero targeting of BSH; Figures 3A-B).

In addition to natural amino acids, unnatural amino acids (UNAAs) have been explored. Some UNAAs, such as 1-aminocycloalkanecarboxylic acid, can participate in BBB transport through special carriers on the cerebral vasculature 55, with stable metabolism. The resulting boron-containing unnatural cyclic compounds (UNAAs) exhibit better water solubility than their natural counterparts. Furthermore, ICP-optical emission spectrometry (ICP-OES) revealed that one of these compounds, cis-ABCPC (Figure 3C), was significantly superior to BPA 56. This superiority was demonstrated by favorable tumor-to-plasma boron ratios in the B16 mouse melanoma model and T/N brain tissue boron ratios in F98 rat gliomas. Another α-amino acid, 1-aminocyclobutanecarboxylic acid (ACBC), binds unnaturally to L-transporter proteins to deliver BSH to tumors. Its fluorinated marker, ^18^F-ACBC, also functions as an effective tumor tracer, which significantly enhances the visualization of the tumor-target area during the verification of BNCT precision and loading 9. The effectiveness of BSH can be significantly enhanced by functionalizing it with ACBC. A study demonstrated that the resulting ACBC-BSH conjugate functioned as an effective ^10^B carrier and that its tans-isomer exerted more significant tumor-cell-killing effects (Figure 3D) 57. The Futamura team 58 observed that the high molecular weight of ACBC-BSH prevented its crossing of the BBB. Thus, they designed and synthesized its trans-isomer (trans-ACBC-BSH), which innovatively leveraged convection-enhanced delivery (CED) to deliver local drugs to tumor sites. The team demonstrated that the introduction of ACBC significantly enhanced the tumor-targeting capacity of BSH. Notably, the ^10^B-absorption capacity of ACBC-BSH in glioma cells was elevated by 2-fold compared with that of unmodified BSH. When administered via CED, the boron concentration of the tumor reached 21.1 μg/g in 1 h, representing a significant increase over the 19.7 μg/g observed for intravenously administered BPA, indicating a more effective local enrichment. Additionally, T/B boron-concentration ratio increased to 14.2, which was superior to that of BPA (6.7). Notably, the combination of ACBC-BSH/CED with BPA prolonged the median survival period of the rats in this treatment group to 44.3 days, which was significantly better than that of the BPA-alone group (37.4 days). This was attributed to the enhanced delivery efficiency of ACBC across the BBB via LAT1 and the CED-driven precise drug penetration of tumor-infiltrated regions. This represents a new strategy for BNCT vector design, i.e., the synergistic optimization of molecularly targeted modifications and local delivery systems.

**Figure 4:**
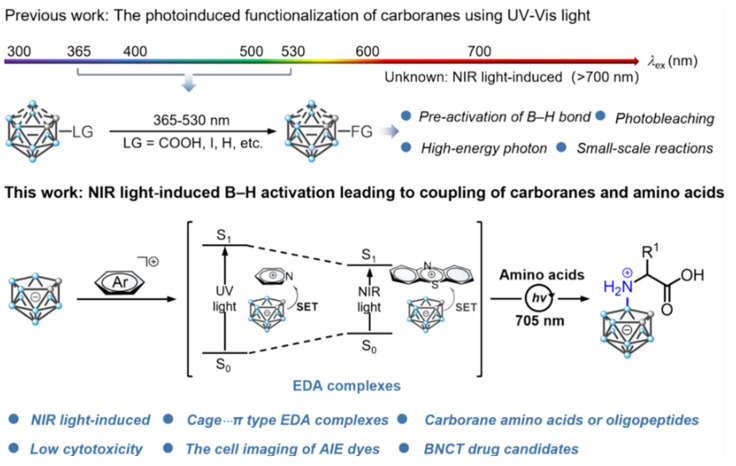
Schematic representation of direct coupling of nido-carboranes to oligopeptides or amino acids using light-induced *nido*-carboranes. Reproduced with permission from Ref. 59. Copyright 2025, ACS.

Other researchers, such as Yan's group, have been exploring the photo-induced B-H functionalization of carboranes 48. Recently, they proposed a new strategy that utilizes near-infrared (NIR) light to functionalize the B-H bonds of inert carborane clusters (nido-carborane). This approach has achieved the efficient coupling of carboranes with amino acids or oligopeptides under mild reaction conditions (Figure 4) 59. The conventional B-H bond functionalization of nido-carborane relies on high-energy ultraviolet (UV) or visible light, which results in low efficiency and numerous side reactions.

To overcome these limitations, an electron-donor-acceptor complex was designed using a carborane and a photocatalyst. Employing NIR photoexcitation, this complex initiated single-electron transfer to generate carborane cage radicals, which subsequently react with amino acids/oligopeptides. Moreover, further modifications, such as introducing fluorescent groups and radioactive iodine, facilitated the synthesis of boron carriers with targeting and imaging functions. Their solubility, stability, and toxicity properties were significantly better than those of existing BNCT drugs, providing a better selection of carriers for BNCT.

#### 2.1.2 Boron-cluster-based porphyrin carriers

Among the various boron-cluster-based drug carriers for BNCT, porphyrins and their derivatives are valuable and efficient in cancer diagnosis and therapy. Their modifiable tetrapyrrole macrocyclic structure, low cytotoxicity, fluorescence, bindability to deoxyribonucleic acid (DNA), and high tumor-cell selectivity and accumulation offer them multiple advantages over clinically approved drugs, such as BPA and BSH 60-62. These advantages are listed below.

a) Ultrahigh boron-loading capacity: their backbones can incorporate multiple boron clusters, which can selectively infiltrate tumor cells in large quantities and accumulate in them, thus significantly enhancing the T/N value.

b) Diagnostic synergistic function: based on the photosensitive properties of porphyrins, they provide a diagnostic synergistic function by enabling tumor localization and therapeutic diagnostics. They can also be combined with photodynamic therapy (PDT) to facilitate multimodal synergistic treatment, which promotes their clinical transformation.

**Figure 5:**
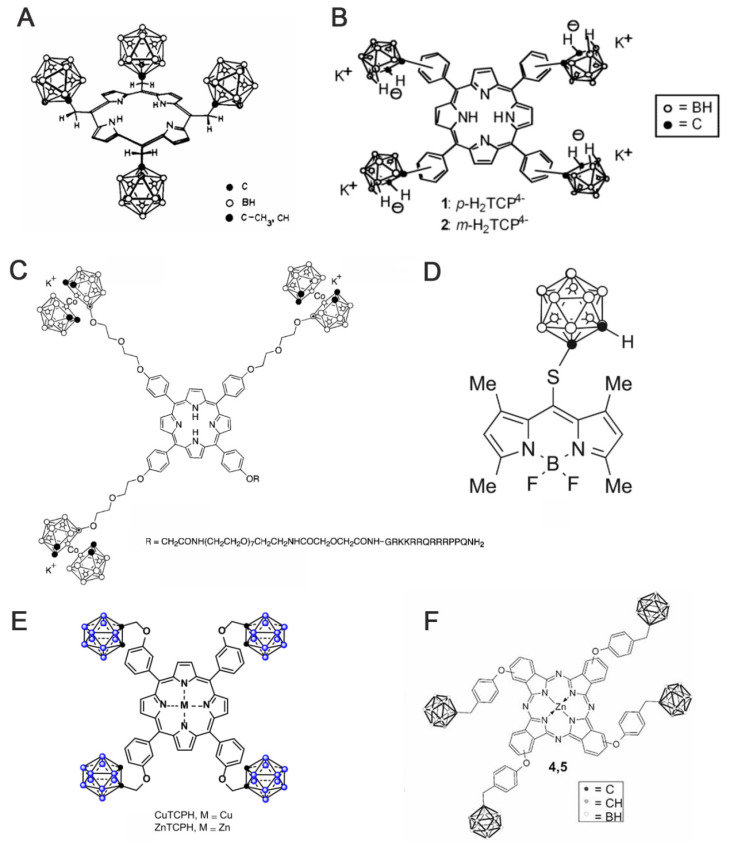
** (A)** α, α, α, β atropisomer of H_2_[P(CH_2_C_2_B_10_H_10_Me)_4_]. Reproduced with permission from Ref. 63. Copyright 1978, ACS. **(B)** Carboranylated porphyrins used in this study. Reproduced with permission from Ref. 68. Copyright 2001, ACS. **(C)** porphyrin-cobaltacarborane-HIV-1 Tat 48-60 conjugate. Reproduced with permission from Ref. 69. Copyright 2006, ACS. **(D)** Structure of BODIPYs 6. Reproduced with permission from Ref. 70. Copyright 2015, Elsevier. **(E)** Structure of CuTCPH and ZnTCPH. Reproduced with permission from Ref. 53. Copyright 2020, Elsevier. **(F)** Structure of ZnB_4_Pc. Reproduced with permission from Ref. 76. Copyright 2006, Springer Nature.

In 1978, Haushalter 63 and Rudolph 64 first synthesized meso-tetracarboranyl porphyrins (Figure 5A), a pioneering achievement that combined boron clusters with porphyrins in an interesting manner. Ever since, several research groups, such as Woodburn's team 65, have reported the synthesis of carbon-boron-based porphyrins (carboranyl porphyrins) and explored their potential in BNCT. Typically, the boron in these tumor-targeting and photosensitizing boron-containing porphyrins derives from hydrophobic pro-carborane, amphiphilic nested carborane, and the cobalt(II) dicarbollide complex 66. Dissimilar to BPA and BSH, boron-containing porphyrins exhibit longer retention time in tumor cells.

Hao *et al.* reported a series of novel porphyrin-cobalt-carborane conjugates with single-molecule boron loading of up to 36 atoms. They optimized the targeting capabilities of these compounds through structural modulations 67. In the series, they observed that Compounds 2 and 4 containing adjacent cobalt-carbon-boron alkyl groups exhibited the highest uptake (up to 0.8 nmol/mg protein in 24 h) in HEp2 cells owing to their amphiphilic nature. Additionally, all conjugates exhibited negligible dark toxicity at 50 μM and a cell survival rate of >75% under phototoxicity. Through the strategy of spatial moiety alignment, they balanced high boron loading with biocompatibility, providing a new paradigm for the design of BNCT carriers. However, the issue of aggregation-mediated loss of delivery efficiency must be addressed.

Furthermore, other research groups, such as Vicente's team, have explored the synthesis and evaluation of various carborane-containing porphyrin derivatives. The key challenge remains the efficient enrichment of these boron agents in tumors during BNCT. To address this, experiments have been conducted on compounds, such as anionic carborane porphyrins (Figure 5A) 68 and cobalt-carborane-porphyrin-HIV-1 Tat 48-60 conjugates (Figure 5C) 69. The anionic carborane porphyrin binds to DNA through protonation or non-covalent interaction, providing a basis for targeting the nucleus. Moreover, the introduction of HIV-1 Tat cell-penetrating peptide significantly enhances the cellular uptake of porphyrin. The study further demonstrated that the targeting and delivery efficiencies of boron-based porphyrins can be effectively optimized by combining chemical modification and functionalized-delivery strategies, providing theoretical support for the development of efficient boron agents for BNCT. To improve the low permeability of the carborane derivatives after integrating different experimental results, the effects of substituent position, the carborane type, and linkage on the fluorescent properties and cellular uptake of carborane-containing boron dipyrrole methylene boron (BODIPY) compounds were systematically investigated (Figure 5D) 70-73. To do this, ortho- and para-carboranes were introduced into the BODIPY molecular backbone using various synthetic strategies (e.g., Suzuki coupling and nucleophilic substitution). Notably, the 8-substituted thio-o-borane-substituted BODIPY (e.g., BODIPY 6) exhibited better BBB permeability than other analogs owing to its low molecular weight and hydrophobicity.

As porphyrins can complex with certain metals (e.g., Cu, Zn, and Mn), researchers have developed two lipid-soluble metalloporphyrins containing carbon-boron alkyl groups, namely CuTCPH and ZnTCPH (Figure 5E) 53, 74. Smilowitz *et al.* employed fluorescence substitution strategies to reveal the distributional properties and safety of these metalloporphyrins as BNCT enhancers 75. They revealed that ZnTCPH traced its uniform distribution in the cytoplasm of liver Kupffer cells of homozygous mice by leveraging the fluorescence property of zinc ions, whereas CuTCPH did not exhibit any fluorescence interference owing to the d-electron quenching effect of copper ions. However, the macroscopic boron distribution was highly consistent with that of ZnTCPH. Remarkably, CuTCPH lacked significant hepatotoxicity at doses up to 400 mg/kg. When combined with BPA, it compensated for the lack of distribution homogeneity for BPA. This study provides a new strategy for developing low toxicity, high-boron-loading BNCT drugs and paves the way for further clinical studies.

Additionally, porphyrin derivatives, such as phthalocyanines, have been further developed. They are structurally similar to porphyrins but comprise four isoindole units. Further, they typically exhibit larger conjugated systems with longer absorption wavelengths, which make them more suitable for deep-tissue treatments. Friso's team proposed an innovative bimodal antitumor strategy based on the synthesis of a four-carborane-modified zinc phthalocyanine (ZnB_4_Pc; Figure 5F). This compound was the first to integrate PDT with BNCT in a single agent 76. Using melanoma as a model, they validated this breakthrough. It demonstrated that the carbon-boron alkyl group introduced at the periphery of the macrocyclic backbone of ZnB_4_Pc conferred ultrahigh boron-loading capacity (40 atoms per molecule) and concurrently retained the excellent photosensitizing properties of phthalocyanine compounds. In vitro experiments have also indicated that ZnB_4_Pc can be efficiently targeted to tumor cells via liposomal delivery, where it can trigger significant oxidative damage under red-light activation. Moreover, neutron irradiation further enhances cytotoxicity through the boron nuclear reaction. Follow-up animal experiments further confirmed that the compound exhibited in vivo tumor-selective accumulation and achieved synergistic efficacy through chronologically regulated PDT (early vascular targeting) and BNCT (late direct cytotoxicity). Owing to the long retention and potential imaging properties of porphyrins, boron-containing porphyrins are highly expected to advance the development of BNCT, especially as this field enters an era of greater precision. While the pursuit of more precise targeting is crucial, the pitfalls of current targeting strategies must also be addressed. Furthermore, there is a need to further advance their clinical translation and generalizability to more cancer types.

#### 2.1.3 Boron-cluster-based peptide carriers

**Figure 6:**
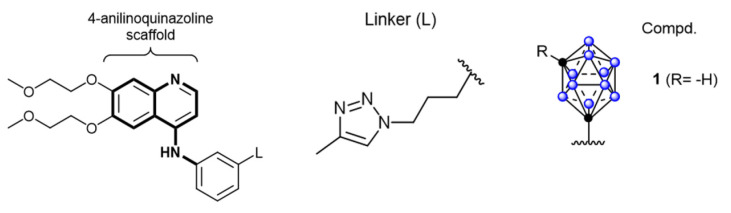
Schematic diagram of the structure of a derivative of closo-caboranyl introduced into 4-aniline quinazoline. Reproduced with permission from Ref. 80. Copyright 2018, Wiley.

**Figure 7:**
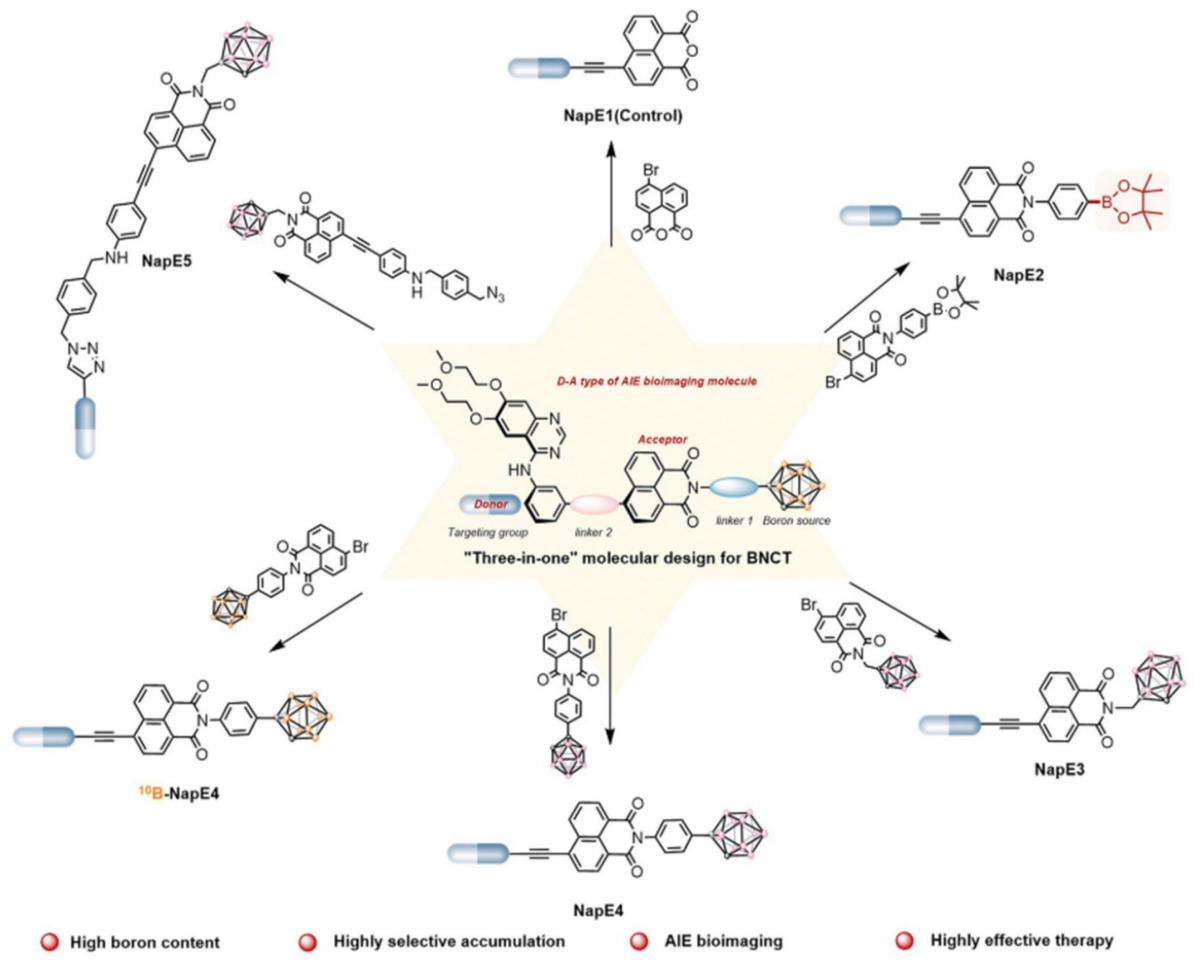
New boron delivery agents for BNCT based on the “three-in-one” molecular design strategy. Reproduced with permission from Ref. 81. Copyright 2024, RSC.

Owing to their high affinity and binding selectivity for receptors and transport proteins specifically overexpressed on the tumor-cell surfaces, peptide molecules have become ideal candidates for boron-targeted delivery systems and a hot research topic. Epidermal growth factor receptors (EGFRs) as receptors for the extracellular protein ligand epidermal growth factor (EGF). As part of the cellular regulatory functions, EGFRs activate downstream signals upon binding to EGF. This activation stimulates physiological processes, such as cell proliferation, differentiation, and cell growth. Critically, the EGFR gene is specifically amplified in primary brain tumors, such as glioblastoma, and its expression is low or absent in normal brain tissues. Studies have demonstrated that this high-frequency gene amplification correlates with the overexpression of receptors on the tumor-cell surfaces 77. The number of receptors on the surface of tumor cells can be more than 100-fold higher than on normal glial cells. This feature has emerged as a key target for the development of precision therapies for brain tumors 78. Consequently, two dominant strategies have formed a clear paradigm for the development of EGFR-targeted therapies for clinical drug discovery. These strategies involve inhibiting the activity of tyrosine kinases or disrupting EGFR signaling by blocking its binding sites 79. The most widely utilized drugs are monoclonal antibodies that target the structural domains of the extracellular receptors, such as cetuximab. Additionally, molecules with a 4-anilinium quinazoline core scaffold, such as gefitinib and erlotinib, have been characterized as highly selective EGFR inhibitors and potent anticancer drugs. Couto *et al.* 80 constructed a novel EGFR bifunctional inhibitor by introducing 1,7-closo-C₂B₁₀H₁₁ as a 3D aromatic bioequivalent to the 4-anilinoquinazoline scaffold (Figure 6). Carboranes exhibit a rigid cage structure with a σ-off-domain electron system, which endows them with unique molecular recognition properties. These properties enable them to form a multiple binding mode through hydrophobic interactions with the Glu762 and Asp855 residues of the EGFR kinase domain. Consequently, the inhibitory activity of the heterodimer against wild-type EGFR (IC_50_ = 2.3 nM) is approximately 10-fold higher than that of erlotinib (IC_50_ = 22.9 nM). Additionally, the ¹⁰B-enriched property of carboranes enables targeted boron accumulation in glioma cells. Combined with its excellent BBB-penetration capability, this makes the molecule synergistic with EGFR-signaling-pathway inhibition and BNCT. This study reaffirms the innovative potential of boron clusters in the design of kinase inhibitors, providing a new template for the development of precision therapeutic regimens for gliomas that combine brain-targeted delivery with radiosensitization. Furthermore, Yan's group 81 proposed a “three-in-one” molecular engineering strategy for designing and synthesizing a novel boron-delivery system that combines targeting, imaging, and therapeutic functions. The system utilizes erlotinib as the targeting unit, which is coupled with boron clusters (C_2_B_10_H_12_) and naphthalimide aggregation-induced emission (AIE) luminescent units via amide bonds or click chemistry, resulting in the NapE series of compounds. Among the compounds in this series, erlotinib targets EGFR-overexpressing lung cancer cells to achieve the selective enrichment of the boron-delivery agent. The carborane cluster provides a high boron-loading capacity of up to 10 ^10^B atoms, ensuring a high T/N. Moreover, the AIE unit enables real-time tracking in vivo and in vitro through its AIE properties. This provides a multifunctional delivery platform for the BNCT of deep-seated tumors (Figure 7).

### 2.2 Boron-cluster nanomedicine

Generally, a promising boron agent must be capable of targeting tumors and delivering large amounts of boron to achieve a T/N value of more than 3:1. It must also exhibit low systemic toxicity, have long retention in the tumor during neutron-irradiation treatment, and be readily eliminated from normal tissues after the treatment. Therefore, the development of boron-cluster nanomedicines is very pivotal, as the auxiliary effects of some nanomaterials can effectively help boron drugs meet these requirements 82-84.

#### 2.2.1 Liposomes

**Figure 8:**
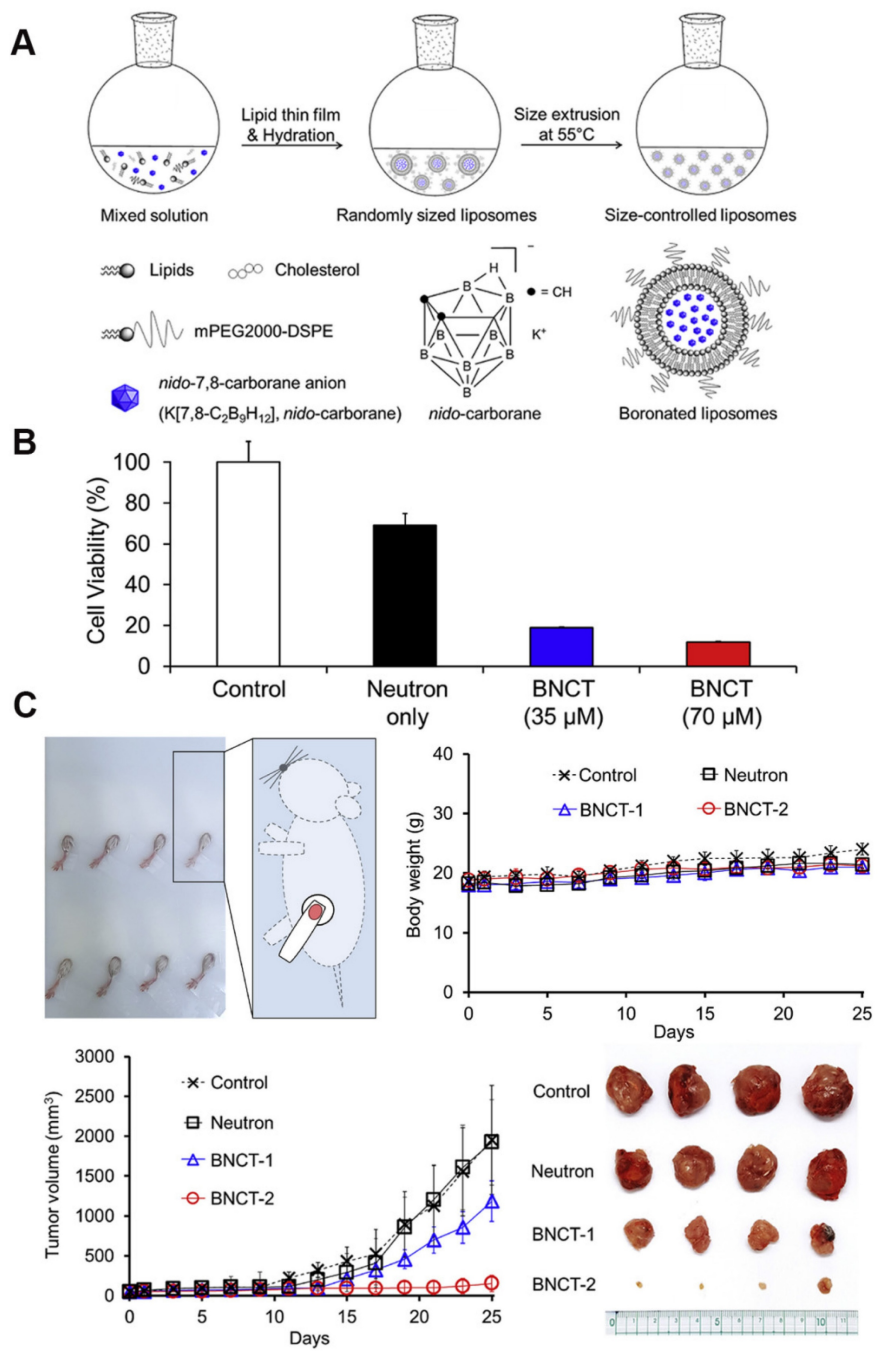
** (A)** Schematic representation of the synthesis and components of boronated liposomes. **(B)** Cytotoxicity of boronized liposomes after thermal neutron irradiation. **(C)** Experimental results of boronized liposomes applied in the CT26 tumor model for in vivo boron neutron capture therapy (BNCT). Reproduced with permission from Ref. 89. Copyright 2020, Elsevier.

Liposomes, the first nanomedicines to enter FDA clinical trials, are vesicles consisting of an aqueous volume surrounded by a lipid bilayer. Owing to their excellent biocompatibility, biodegradability, non-toxicity, and controlled-release properties, liposomes are widely employed for therapeutic and diagnostic applications. Owing to the high endocytosis activity of some tumor cells and the increased local permeability of capillaries, small liposomes tend to accumulate much more in tumor tissues than in normal tissues, a phenomenon that has also been described as the “enhanced permeability and retention effect (EPR).” Furthermore, the targeting properties of liposomes can be readily improved because of the ease of modifying their surfaces compared with other drug carriers. Numerous methods have been developed to activate the targeting properties of liposomes by binding them to various tumor-targeting carriers. Consequently, liposomal boron-delivery systems are also considered effective for BNCT because they can carry large amounts of boron compounds. Since 1990, several studies have attempted the encapsulation of boron-containing compounds in liposomes for BNCT 85-87. Further, the following two methods have been explored to achieve the utilization of liposomes as boron carriers: a) the encapsulation of boron compounds into liposomes and b) the doping of boron-conjugated lipids into the liposome bilayer. Early experimental studies typically adopted the first approach to improve the targeting effect of boron clusters. For instance, Shelly *et al.* 88 encapsulated a variety of highly water-soluble boron-cluster compounds (e.g., B₁₀H₁₀^2-^ and B₂₀H₁₈^2-^) into liposomes, significantly enhancing their tumor-targeting efficiency for BNCT. Experiments demonstrated that the liposomes accumulated selectively in tumors via the enhanced permeability of tumor vasculature (the EPR effect). Thus, the boron concentration of the tumor reached a therapeutic threshold (>15 μg/g) and T/B exceeded 3:1 after 48 h of a single intravenous injection 88. As illustrated in Figure 8, Lee *et al.* 89 innovatively leveraged freeze-thaw cycling to efficiently encapsulate water-soluble nested carbocyanine in the hydrophilic core of polyethylene glycol (PEG)-attached liposomes. By leveraging the EPR effect, these modified liposomes achieved deep tumor penetration and intracellular distribution. A single injection of 21 mg of ^10^B/kg combined with 20 min of neutron-irradiation resulted in an 88.2% tumor-inhibition rate without systemic toxicity. This approach is significant as it simplifies the complex synthesis required for conventional carbocyanine owing to its modification requirements. This study circumvented the complicated synthesis of traditional carbocyanine and simplified the experimental steps by implementing “direct encapsulation without modification.” However, this approach is susceptible to the risk of hepatic metabolism due to the prolonged retention of liposomes. Although this shortcoming was not mentioned in the paper, it remains an issue that must be resolved.

**Figure 9:**
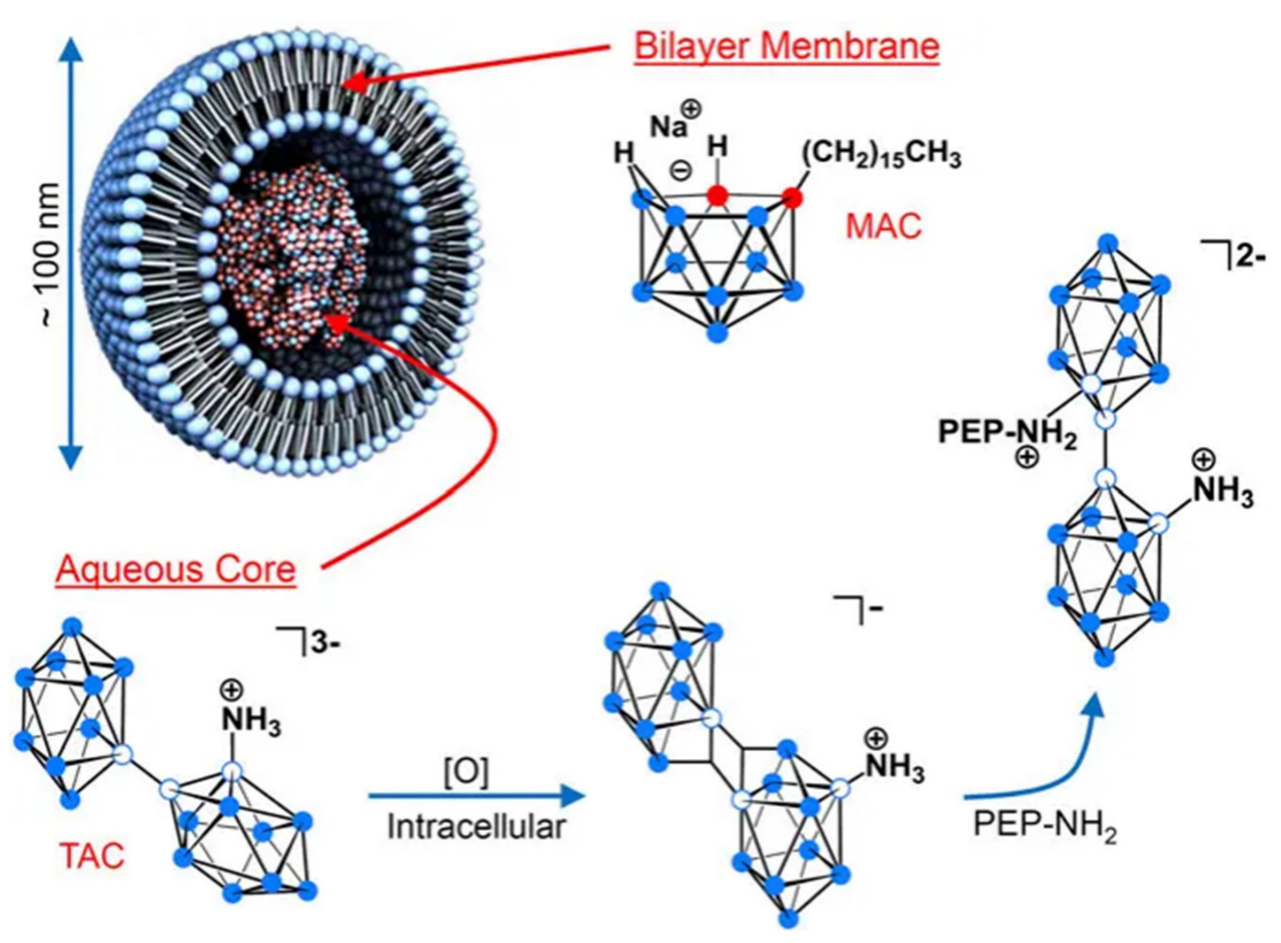
Liposomal formulation in which the lecithin/cholesterol bilayermembrane incorporates the lipophilic boron agent MAC, acting as a complement to the hydrophilic polyhedral borane TAC, which is encapsulated in the aqueous core. Reproduced with permission from Ref. 90. Copyright 2013, NAS.

**Figure 10:**
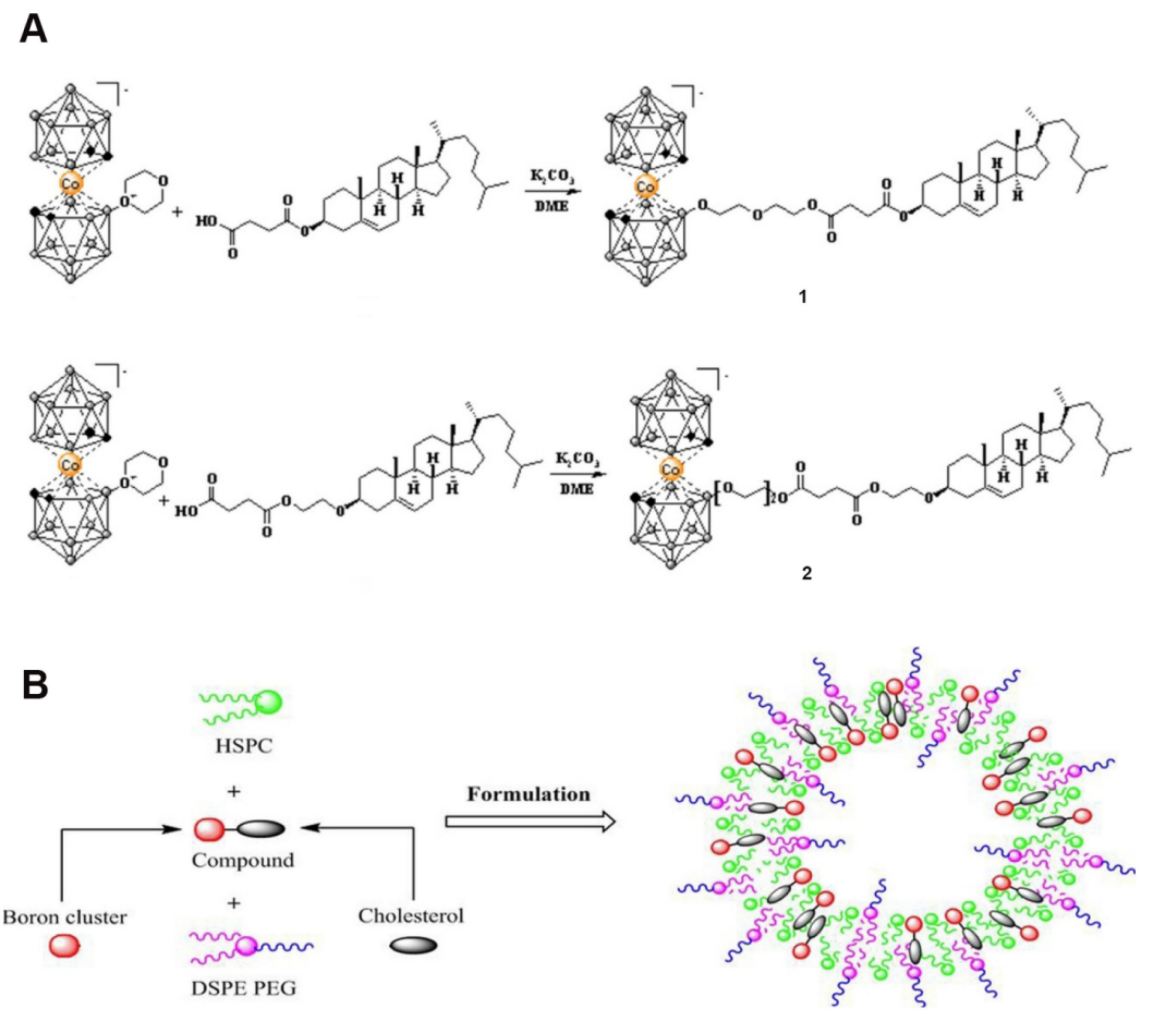
** (A)** Synthesis of boronated cholesterol 1 and 2 using the 1,4-dioxane derivative of cobalt bis(dicarbollide) with cholesterol-based carboxylates. **(B)** Schematic representation of the liposomal formulation of cholesterol derivatives of Boron clusters. Reproduced with permission from Ref. 91. Copyright 2020, Wiley.

As shown in Figure 9, Hawthornex addressed key limitations of BNCT for cancer treatment by designing a liposome-based boron-delivery system. The study achieved selective delivery of boron by constructing liposome monolayers containing two different compounds: a hydrophilic polyhedral borane (TAC) encapsulated in the aqueous core of the liposome, and a lipophilic nested carborane (MAC) embedded in its membrane layer. This strategic design was used to exploit the tumor-EPR effect 90. The boron concentration of the tumor reached 67 μg/g in 54 h (significantly exceeding the therapeutic threshold, 20 μg/g), and the T/B value increased to 5.68:1 in 96 h, thereby optimizing the delivery efficiency. Thermal-neutron-irradiation experiments revealed that the 14-day tumor volume in the BNCT group was much smaller than that in the control group and that a second treatment or prolonged irradiation further inhibited tumor growth, with no toxicity or radiation side effects. This study validates the effectiveness and safety of liposome delivery systems in BNCT, providing experimental evidence for clinical translation. Based on this, Hawthorne's team explored the effectiveness of this liposome delivery system in other cancer types, such as oral and colon cancer, further expanding BNCT to the management of other cancer types. Second, the boron-embedding efficiency in the first method was relatively small owing to osmotic factors. Consequently, the liposome dose required to deliver sufficient boron atoms to tumor tissue is considerably large. Notably, high doses are traditionally pre-injected to achieve targeting by saturating the clearance capacity of the liver. However, such high liposome doses may lead to hepatotoxicity owing to the limited clearance function of the liver. Therefore, using low lipid doses may prevent lipid-uptake-saturation-induced hepatotoxicity and achieve consistent pharmacokinetic behavior in repeated injections, as lipid-uptake saturation in the liver is a common issue with high lipid doses. Therefore, to reduce the total liposomal dose, liposomes with higher boron content must be developed by incorporating boron-conjugated lipids into the liposomal bilayer. This approach is necessary for realizing the practical application of liposomes as a boron-delivery system. Bregadze's team constructed novel boron liposomes by covalently conjugating polyhedral boranes with cholesterol (Figure 10) 91. They employed a chemically modified strategy to directly integrate the boron cluster into the lipid bilayer, thereby avoiding the inefficiency of traditional physical encapsulation and significantly enhancing the boron loading. Moreover, the cobalt bicarbonylboron alkyl liposomes exhibited a survival rate of more than 95% in normal human mammary epithelial cells (MCF-10A) and BC cells (MCF-7), confirming their low toxicity. Furthermore, the chemical binding of boron clusters to lipids reduced the hepatic accumulation of free boron (the hepatic uptake was reduced by 60% compared with the uptake in conventional encapsulation), providing a new strategy for overcoming the high-dose hepatotoxicity possibility of conventional delivery systems. This strategy achieves a balance between efficient boron delivery and biosafety through the “boron cluster-lipid hybridization.”

As boron carriers, there have been significant breakthroughs in the development and engineering of BNCT liposomes. Their core advantages are the ability to achieve passive tumor targeting via the EPR effect, as well as to achieve high boron loading. However, the lack of long-term toxicity assessment further complicates their clinical translation. Moreover, the balance between cost and safety remains a key to achieving a breakthrough in the clinical “last kilometer.”

**Figure 11:**
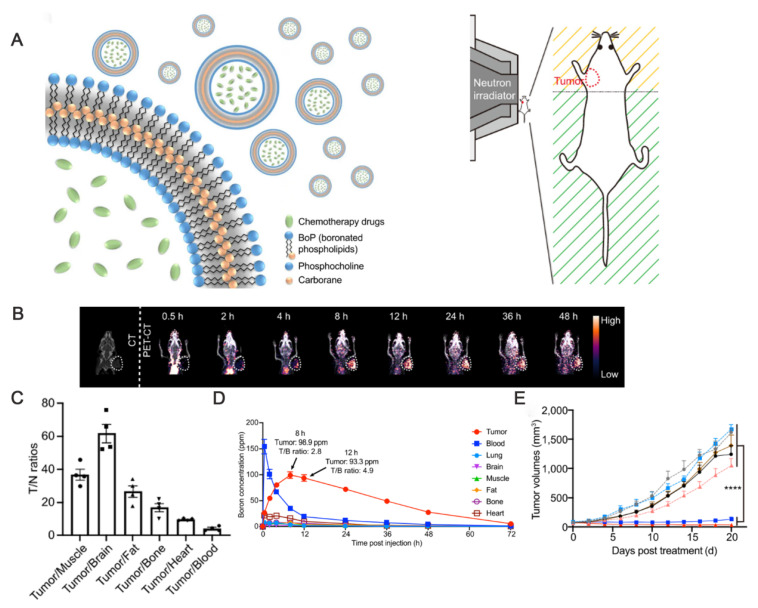
** (A)** Schematic diagram of Boronsome. **(B)** Representative CT and PET-CT images. **(C)** Tumour-to normal tissues ratios (T/N). **(D)** Changes in boron concentrations over time in tumors, blood, lungs, brain, muscles, fat, bones, and heart. **e** Mean tumor volume of mice in each group. Reproduced with permission from Ref. 92. Copyright. 2022, Springer Nature.

Thus, researchers are exploring the possibility of combining BNCT with other therapies using liposomes as carriers. Moreover, other clinical modalities, such as chemotherapy and immunotherapy, can be used to overcome the shortcomings of BNCT and provide patients with more comprehensive treatment options. For example, Liu's group developed a novel boron-loaded liposome, “boronsome,” to simultaneously deliver boron and chemotherapeutic drugs (e.g., doxorubicin or PARP1 inhibitors), thus exerting synergistic antitumor effects (Figure 11) 92.

**Figure 12:**
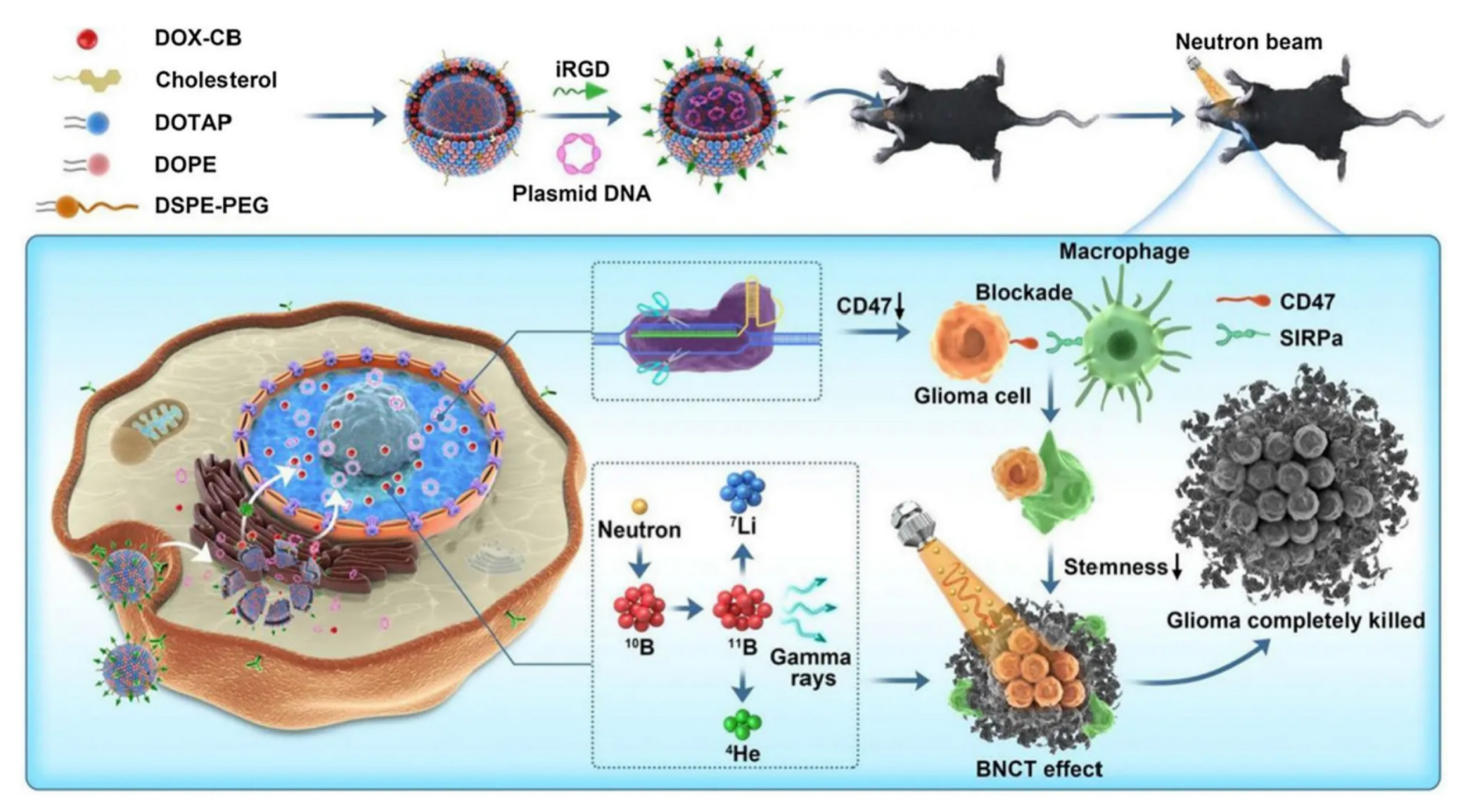
DOX-CB@lipo-pDNA-iRGD Schematic diagram of the mechanism. Reproduced with permission from Ref. 93. Copyright. 2022, Springer Nature.

The innovative covalent attachment of carbon-boron alkyl groups to the hydrophobic tails of phospholipids forms a stable membrane structure. This design improves boron loading and tumor targeting, as well as allows for the encapsulation of chemotherapeutic drugs within its internal cavities. Under neutron irradiation, the ^10^B(n,α)^7^Li reaction generates high linear energy transfer (LET) particles that damage tumor DNAs. The encapsulated PARP1 inhibitor simultaneously prevents DNA repair, and this synergistic effect significantly enhances the antitumor effect. Han and Wei's group 93 designed a nuclear-targeted boron-delivery agent, doxorubicin complexed with carborane (DOX-CB), which was combined with a multifunctional nanoliposome system to synergize BNCT with chemo-/immunotherapy (Figure 12). Leveraging its nucleotropic properties, DOX delivers CB to the nucleus of the tumor cells, and ^10^B in CB undergoes a nuclear reaction to produce high-LET particles that directly damage DNA. Concurrently, the nanoliposomes carry the CRISPR-Cas9 plasmid to knock down the CD47 gene of tumor cells. This action blocks the “don't eat me” signal (the CD47-SIRPα pathway), thereby activating the phagocytosis of macrophages and reducing the characteristics of tumor stem cells to inhibit tumor recurrence.

#### 2.2.2 Other nanomaterials

The development of other nanomaterials, such as carbon nanomaterials, mesoporous silica nanoparticles (NPs, gold NPs (AuNPs), and polymers, provides a wide variety of options for BNCT-targeted boron carriers.

**Figure 13:**
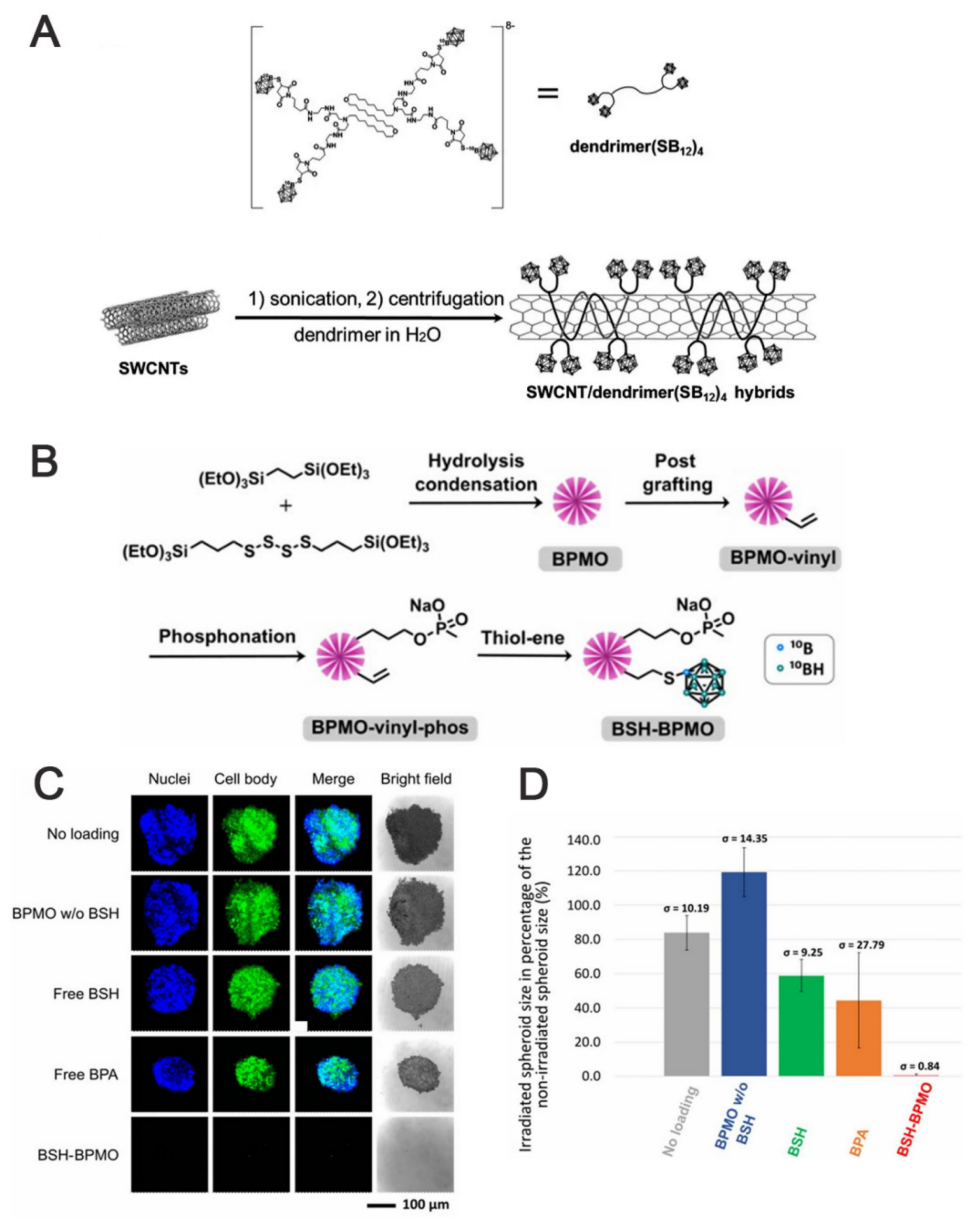
** (A)** Schematic illustration of the dendrimers and Fabrication of the SWCNT/**dendrimer(SB_12_)_4_** nanohybrids. Reproduced with permission from Ref. 94. Copyright 2018, Wiley. **(B)** BSH-BPMO synthesis process. **(C)** Image of OVCAR8 spheroids after 1 hour of neutron irradiation and 3 days of incubation under confocal microscopy. **(D)** Comparison of the contraction rate of OVCAR8 tumor globules after neutron irradiation. Reproduced with permission from Ref. 95. Copyright 2023, RSC.

**Figure 14:**
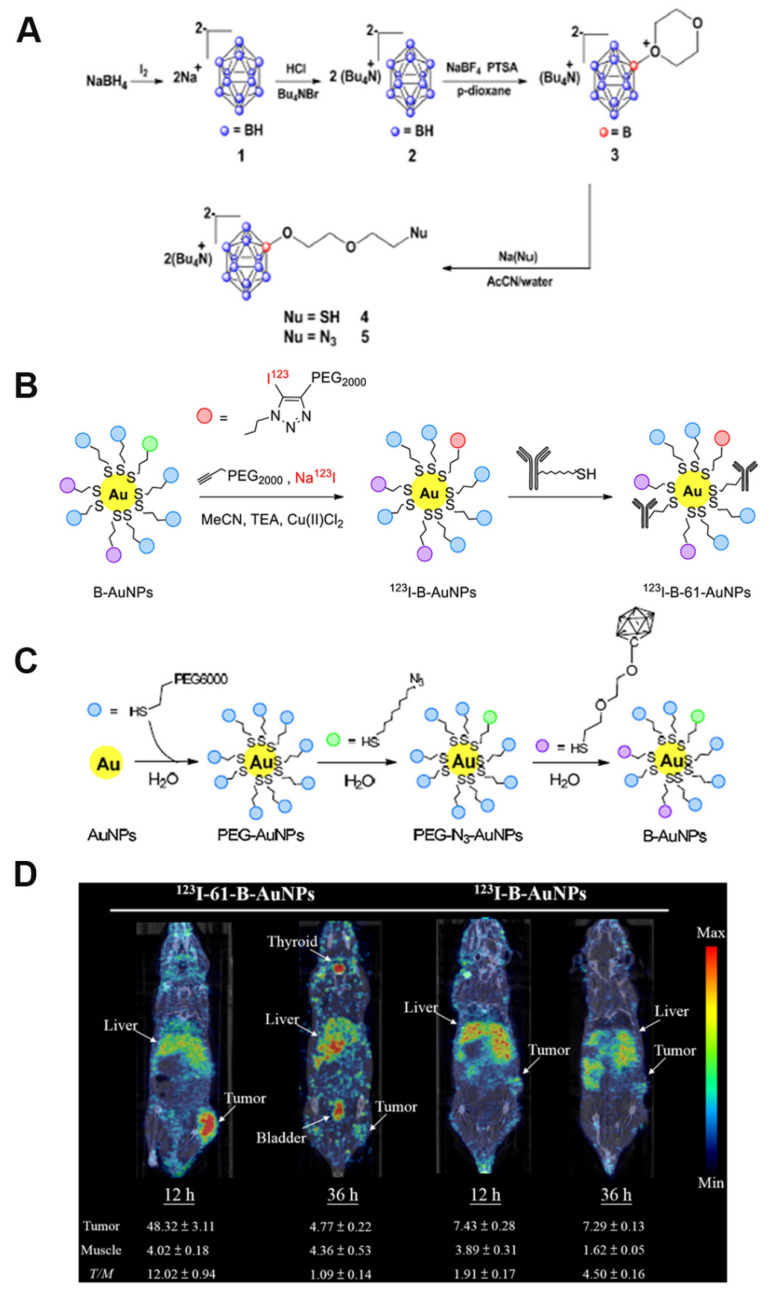
** (A)** Synthesis of boron cage-SH.** (B, C)** Synthesis of ^123^I-B-AuNPs and ^123^I-61-B-AuNPs. **(D)** SPECT/CT imaging images of mice 12 and 36 hours after injection of ^123^I-B-AuNPs and ^123^I-61-B-AuNPs.Reproduced with permission from Ref. 96. Copyright 2019, Elsevier.

For example, single-walled carbon nanotubes (SWCNTs), which are nanomaterials that can be utilized for in vivo fluorescence imaging, can also be employed as carriers for drug delivery. For instance, Yamagami *et al.* designed an SWCNT-based multifunctional nanohybrid system to endow BNCT boron agents with enhanced tumor targeting and in vivo imaging functions (Figure 13A) 94. The SWCNT/dendrimer (SB12)4 nanohybrids were prepared by first synthesizing a polyamidoamine-amine dendrimer (dendrimer (SB12)4) containing BSH terminals and using it to non-covalently modify SWCNTs. The hybrids retained the fluorescence properties of SWCNTs in the NIR-II region without destroying the SWCNT structure. This endowed them with in vivo imaging properties and enhanced their application for real-time in vivo monitoring and precision therapy in BNCT. As shown in Figures 13B-D, Mathilde *et al.* 95 designed and synthesized a mesoporous organosilicon nanoparticle skeleton, which was covalently loaded with BSH (BSH-BPMO) to address the limited efficacy of BSH in BNCT owing to its poor cellular uptake: BPMO was constructed via Schiff base condensation, after which BSH was stabilized and anchored by vinyl modification and thiol-enclave clicking reaction. Finally, a negatively charged surface was produced, i.e., nanocarriers with negatively charged surfaces and a particle size of approximately 300 nm. Experimental evaluations indicated that BSH-BPMO could be efficiently endocytosed by ovarian cancer cells (OVCAR8) and localized in the perinuclear region. Additionally, its boron uptake was significantly higher than that of free BSH. Further, this nanocarrier enhanced the BSH-by-BSH cell uptake and allowed for the expansion of the BSH application in other cancer therapies, thus significantly enhancing the BNCT effect. AuNPs have attracted attention in tumor therapy for their unique physicochemical properties and modifiability. Their tunable size can be leveraged to optimize the passive targeting of tumors via the EPR effect. Additionally, their surface can be modified with targeting ligands for active tumor-receptor recognition. The neutron-activated Au nucleus (¹⁹⁷Au→¹⁹⁸Au) can also be employed in BNCT to release β-particles, which synergistically enhance the tumor-cell-killing effect using boron-neutron-captured α-particles, making it a groundbreaking advancement in BNCT. The HER2-targeting AuNP-CB complexes (61-B-AuNPs) developed by Wu *et al.* 96 (Figure 14) were modified on the surface of 20 nm Au cores by click chemistry. Subsequently, they were coupled with an anti-HER2 antibody (61 IgG), and the boron distribution therein was monitored in real time via SPECT/CT imaging after labeling with the radioactive element, ^123^I. The IgG recognition of HER2 epitopes triggered receptor-mediated internalization, resulting in a three-fold increase in intracellular boron concentration. The integration with imaging technology also promoted diagnostic and therapeutic consolidation.

**Figure 15:**
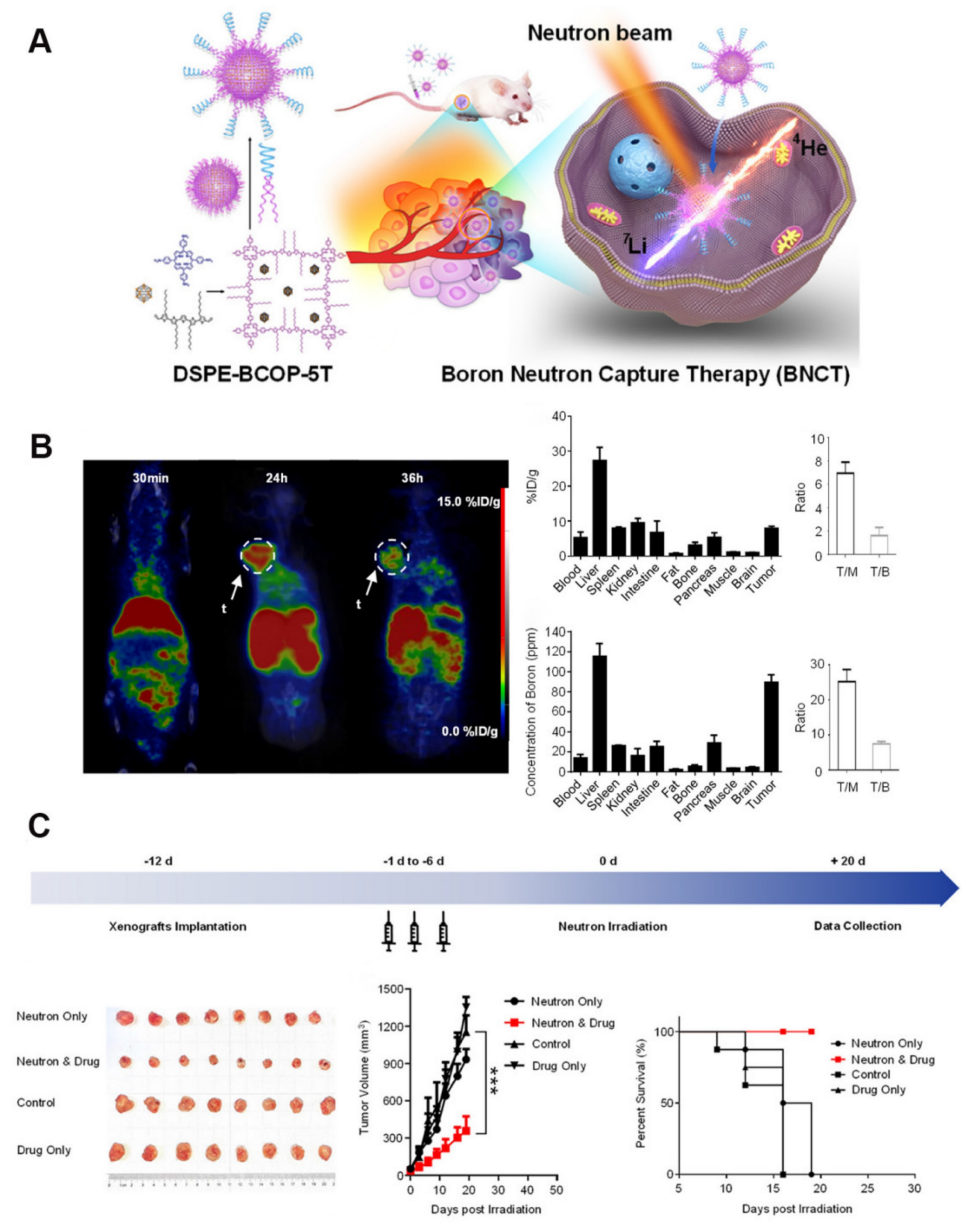
** (A)** Schematic of DSPE-BCOP-5T for boron neutron capture therapy. **(B)** Biodistribution map and experimental data of ^64^Cu-DSPE-BCOP-5T in hormonal mice. **(C)** Experimental protocols for BNCT treatment, morphological observations of BNCT experimental tumors, and average tumor volume in each group of mice. Reproduced with permission from Ref. 97. Copyright 2020, ACS.

Liu's group 97 constructed a nanoscale covalent organic polymer (COP) carrier, DSPE-BCOP-5T, through Schiff base condensation. This innovative design directly encapsulated CB into the COP skeleton and leveraged the porphyrin structure to chelate ⁶⁴Cu for real-time positron emission tomography (PET) imaging. It increased the boron concentration of tumors to 84.93 ppm (four-fold higher than the therapeutic threshold) in a BC model based on a thrice-dosed strategy. When combined with neutron irradiation, the system achieved significant tumor suppression without systemic toxicity. Although the split-injection strategy increased the clinical burden, it optimized the EPR effect. Overall, this type of organic carrier can be a next-generation boron agent capable of monitoring and therapeutic functions (Figure 15).

**Figure 16:**
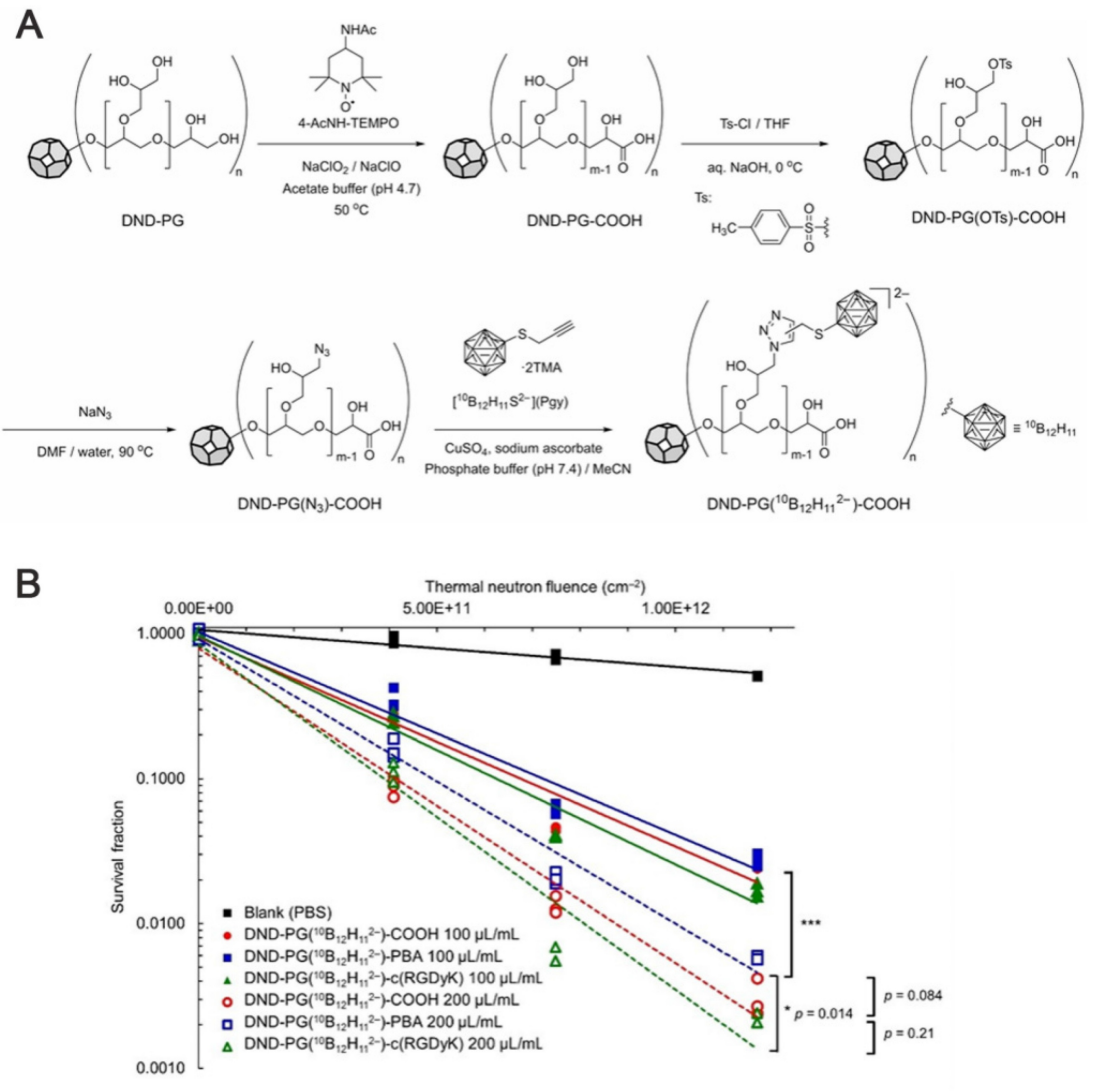
** (A)** Drug synthesis route based on DND-PG. **(B)** Colony formation assay results after B16 cells were treated with functionalized nanomedicines and exposed to thermal neutron irradiation. Reproduced with permission from Ref. 98. Copyright 2023, Wiley.

Nishikawa *et al.* 98 addressed the insufficient targeting and boron-drug accumulation in BNCT by designing a modular nanodelivery system based on poly(glycerol) functionalized nanodiamond. They employed click chemistry to covalently couple a ^10^B-enriched cluster (^10^B_12_H_11_^2-^) to the poly(L-lysine) dendrimer (DND) modified by a grafted PEG surface. By modifying the carboxyl sites with phenylboronic acid or an RGD peptide as active targeting ligands, they constructed a nanomedicine exhibiting high boron loading and dual-targeting functionalities. Following thermal-neutron irradiation, the nanomedicine demonstrated significant cytotoxicity. Their study provided novel insights for designing boron-delivery agents for BNCT, and its modular functionalization concept further advanced the development of multi-modal tumor therapy nanoplatforms (Figure 16).

In conclusion, the targeting, tumor enrichment, and biosafety of boron agents are the core factors that determine their efficacy. Various boron-delivery agents can be considered efficient tools for improving BNCT efficiency. In this section, the key performance parameters of recently developed boron systems were clarified, including their targeting mechanisms, boron concentrations of the targeted tumors, and their T/B and T/N values (Table 1). These clarifications allowed for the visual comparison of the advantages and limitations of different boron agents. Overall, these delivery systems further enhanced boron accumulation within tumors and facilitated disease diagnosis, as well as the development of personalized BNCT.

## 3. Anticancer mechanism of boron neutron-capture therapy

As a targeted radiotherapy, the anticancer mechanism of BNCT involves inducing tumor-cell-specific DNA damage. This is achieved using high LET particles, which are released during a nuclear reaction and overwhelm the cellular DNA-repair system to achieve precision killing. LET and the relative biological effect (RBE) are fundamental radiobiology concepts. LET refers to the number of ionizations that are induced per unit radiation distance. Generally, ^7^Li^3+^ facilitates a high LET (~100 keV/μm), whereas γ-particles facilitate a much lower LET (~0.2 keV/μm). The RBE of a given radiation refers to the reference radiation dose that exerts the same biological effect as that radiation, typically X-rays. The RBE of BNCTs significantly depends on their LET. The physical basis of BNCT relies on the nuclear reaction between the stable isotope (^10^B) and a thermal-neutron, represented by the notation, ^10^B (n,α)^7^Li. Briefly, when ¹⁰B captures a neutron, it undergoes fission (^10^B (n,α)^7^Li), releasing high-LET particles: ^4^He^2+^ and ^7^Li^3+^.

**Figure 17:**
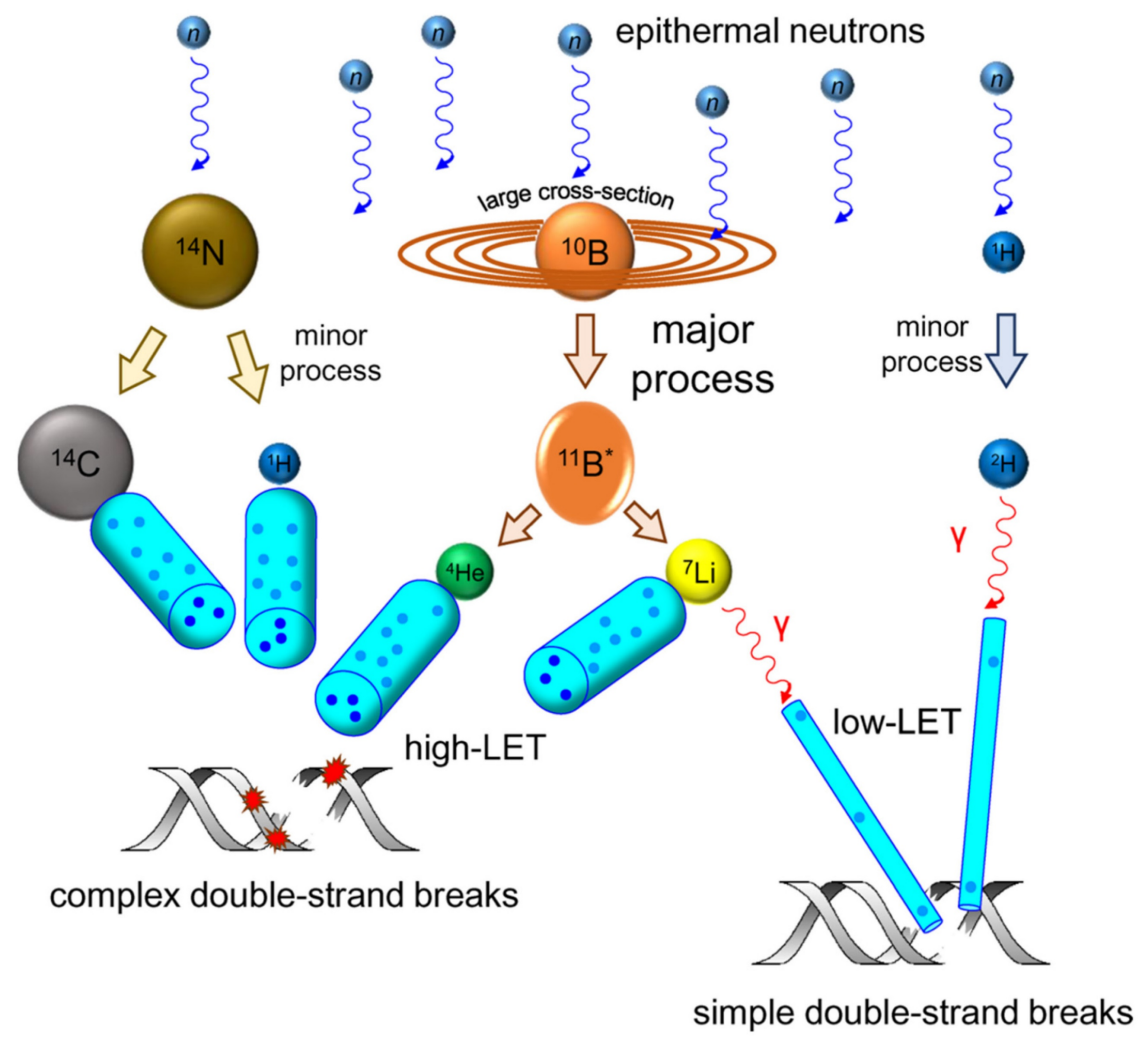
Schematic depiction of nuclear reactions, ionization processes, and DNA damage taking place during BNCT. Reproduced with permission from Ref. 102. Copyright 2022, MDPI.

Both particles exhibit extremely short ranges (4-9 μm), covering only a single-cell diameter range. This ensures that the energy deposition is confined to boron-containing tumor cells, thus preventing cascading damage to surrounding normal tissues. This feature renders BNCT particularly suitable for the treatment of infiltrating tumors (e.g., glioblastoma), whereas conventional radiotherapy can barely achieve similar selectivity owing to physical dose-distribution limitations. To date, BNCT has been clinically studied across a variety of disease sites. Ionizing radiation (IR) exerts anticancer effects by inducing DNA damage that impairs cancer-cell proliferation or by damaging healthy cells 99. A mixture of primary and secondary particles of various energies is involved in BNCT. Although ^4^He^2+^ and ^7^Li^3+^, as high-LET particles, exhibit significantly higher ionization densities than low-LET radiations, such as γ-rays, these low-LET radiations typically induce isolated DNA damage, such as double-strand breaks (DSBs) and single-strand breaks (SSBs). Additionally, the proportion of clustered/complex DNA damage (CDD) increases with the increasing LET of the radiation 100, 101. Therefore, high-LET ^4^He^2+^ are more likely to produce CDDs. Therefore, the frequency and complexity of CDD increase in high-LET ^4^He^2+^ than in low-LET radiations. However, CDD, which contains multiple DNA damages within one or two helical DNA turns, is much more challenging to repair, and in some cases, it cannot be repaired (Figure 17) 102. Radiation damages various parts of the cell, including the cell membrane, cytoplasm, and nucleus, although DNA damage may account for the most significant cause of mitotic death. Unrepaired breaks result in cell death, whereas poorly repaired breaks increase the likelihood of chromosomal rearrangements, mutagenesis, and the loss of deterministic genetic information. Normally, a cell will continue its cycle if DNA damage can be completely and correctly repaired. Otherwise, the cell will undergo cell-cycle arrest in the G2/M phase, apoptosis resulting in cell death, mitotic catastrophe (MC), or senescence.

**Figure 18:**
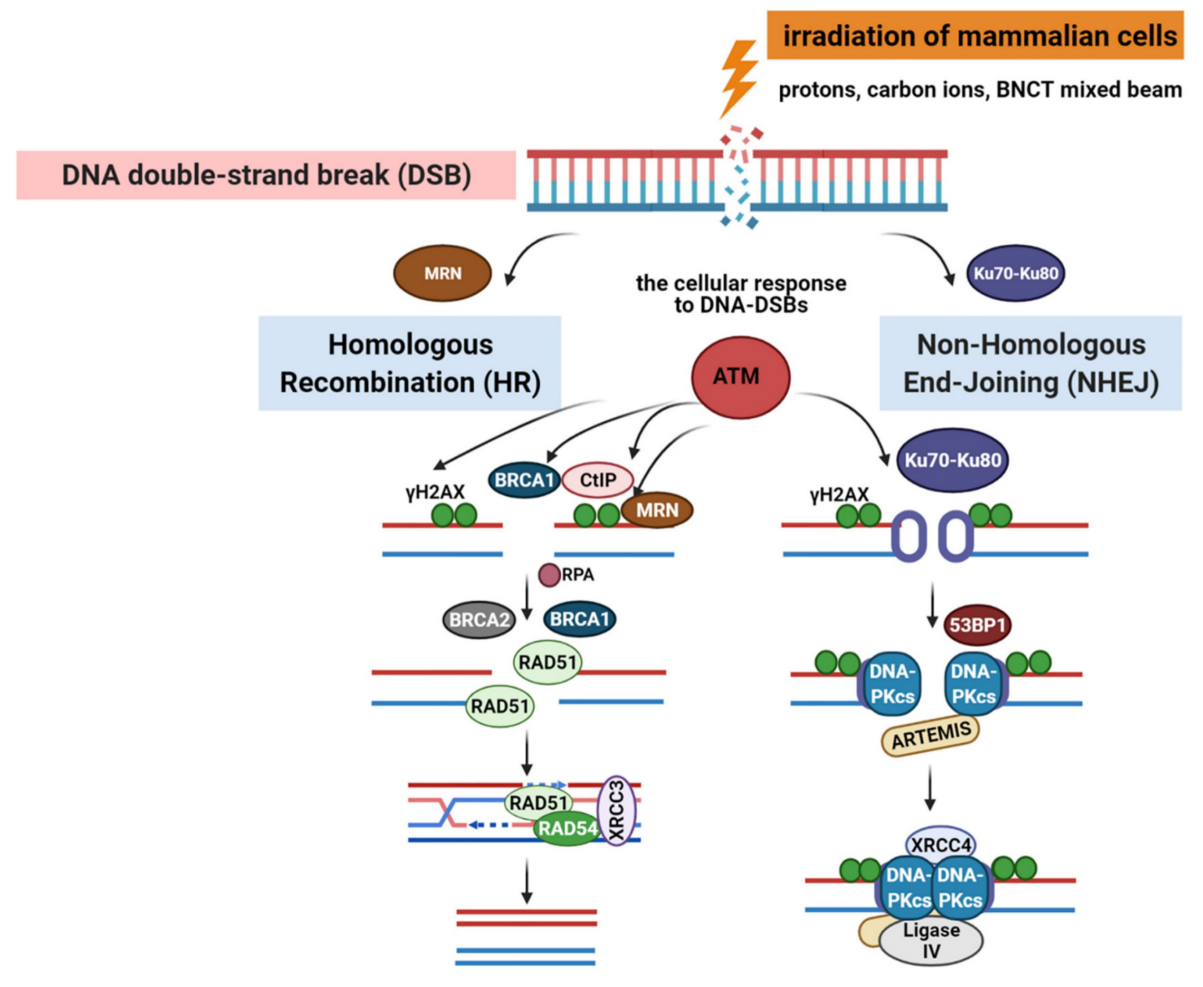
DNA-double-stranded breaks repair pathways: homologous recombination repair (HRR) and non-homologous end-joining (NHEJ) pathway induced by low and high linear energy transfer (LET) radiation. Reproduced with permission from Ref. 104. Copyright 2020, Frontiers.

**Figure 19:**
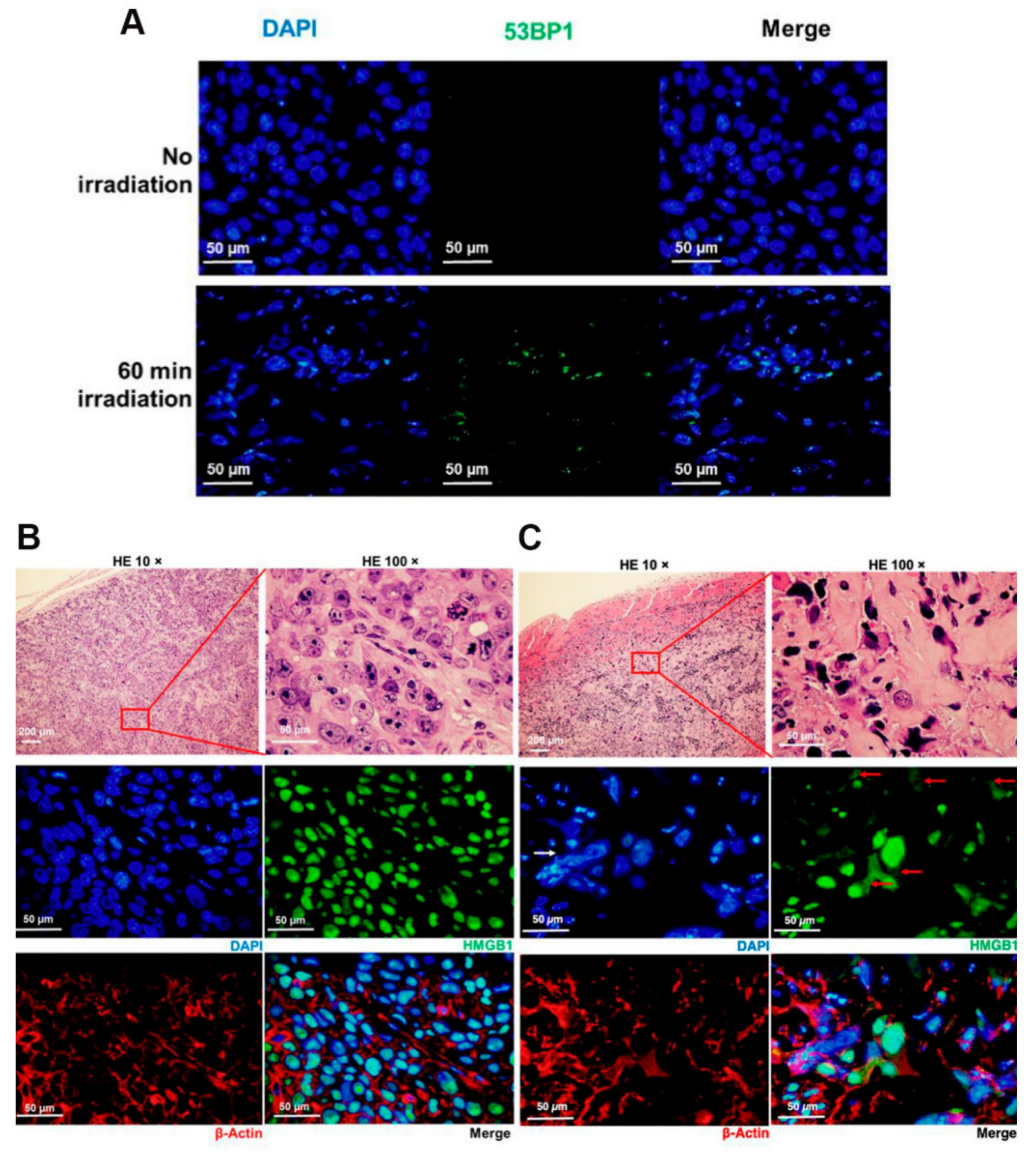
SAS cells were subcutaneously grafted in nude mice and 30 min after administration of BPA-fructose at 500 mg/kg bodyweight, tumors were mock-irradiated. **(A)** Immunostaining of 53BP1 in sections from tumor xenografts at day 3. **(B, C)** Immunostaining of the HMGB1 (green) and β-actin (red) in sections from tumor xenograft-bearing mice at day 3. In **(C)**, HMGB1 panel, solid red arrow shows the distribution of HMGB1 in the cytoplasm; solid white arrow shows the irregular nuclear morphology. Reproduced with permission from Ref. 106. Copyright 2022, MDPI.

In higher eukaryotes, radiation-induced DNA-DSBs are among the most lethal types of genomic damage. To counter this, cells have developed two main repair mechanisms: non-homologous end-joining (NHEJ) or homologous recombination repair (HRR) pathways. However, the efficiency of these repair pathways is closely related to the cell type and cell-cycle stage 103. HRR is a highly accurate repair pathway that utilizes sister chromatids as templates. This process primarily occurs during the late S- and G2/M-phases of the cell cycle. HRR relies on the Rad52 epistasis gene family, with Rad51 and Rad54 acting as key proteins. Rad51 binds to single-stranded DNA (ssDNA) to facilitate the search for homologous sequences and DNA-strand exchanges, whereas Rad54 activates the pairing function of Rad51 104. NHEJ functions at all periods of the cell cycle, including the G0/G1 phases. Additionally, the major factors in the NHEJ pathway, the Ku70/Ku80 heterodimers, are highly abundant in human cells and exhibit a high affinity for DSBs. Upon recognizing DSB, Ku70/80 binds to the ends of the break and recruits DNA-dependent protein kinases (DNA-PKcs) to complete the repair process (Figure 18). To further evaluate the post-BNCT cellular response, detecting its early and late markers is necessary. Poly(ADP-ribose) represents another immediate marker for SSB and DSB. It is synthesized by poly(ADP-ribose) polymerase (PARP) in the nucleus. PARP-1 is a major molecule that is activated by DNA breakage, whereas PARP-2 and PARP-3 are activated by DNA lesions. These PARP molecules are involved in SSB and DSB DNA repairs, as well as oxidative lesions via base-excision repair. After DNA damage, PARP regulates the HMGB1 translocation 105, whereas HMGB1, an early marker, usually binds directly to various bulk DNA damages and participates in DNA-repair pathways, including base-excision repair, DNA-mismatch repair, and NHEJ. HMGB1 deficiency causes DNA damage and reduces its repair efficiency. Imamichi *et al.* revealed the potential of HMGB1 as a biomarker for early efficacy assessment by investigating its dynamic release during BNCT 106. Employing human squamous cell carcinoma (SAS) and melanoma (A375) cell lines on an SAS xenograft mouse model, they observed that extracellular HMGB1 release was significantly higher than that of an equivalent dose of γ-irradiation 24 h after BNCT neutron-beam irradiation (boron-containing carrier, BPA). The in vivo experiments revealed that plasma HMGB1 levels in the BNCT-treated mice were significantly elevated on the third day and remained highly expressed after tumor-volume reduction (Day 8; Figure 19A). The following immunohistochemistry revealed that HMGB1 was translocated from the nucleus to the cytoplasm of the BNCT-treated tumor cells (Figures 19B-C), indicating that its release was associated with cell death. Furthermore, the BNCT-induced DNA-damage marker, 53BP1, was significantly enriched in the tumor tissues, further validating the irreparable clustered DNA damage caused by high-LET particles (^4^He^2+^ and ^7^Li^3+^). This study was the first to demonstrate the close association between extracellular HMGB1 release with the early tumor-cell-killing effect of BNCT. As a late marker, the earliest detectable response is the phosphorylation of histone γ-H2AX at the serine 139 site to produce a focal fluorescent-antibody-detectable product 107. Further, p53-binding protein 1 (53BP1) serves as another crucial marker that typically binds to DNA-DSBs. It contributes to the regulations ofHRR and NHEJ. Additionally, γ-H2AX and 53BP1 foci are well-known IR-induced foci for quantifying DNA damage 108. For instance, Rodriguez *et al.* induced high-LET particles via the ^10^B(n,α)^7^Li reaction and observed that the BNCT group formed γ-H2AX foci with significantly larger volume after 24 h, dissimilar to groups treated with only a neutron beam or γ-rays. Additionally, Kondo's group 109 compared the retention times of γ-H2AX foci generated by γ-rays and the ^10^B(n,α)^7^Li reaction over a 24h period. They observed that γ-H2AX in the neutron-ray group persisted in the tumor cells for more than 24 h, demonstrating again that high LET produces more complex and poorly repaired DSBs. Similar results have been reported using different cancer-cell models. Kinashi *et al.* 110 revealed the unique biological effects of fractionated irradiation in BNCT by comparing the effects of a fractionated neutron beam with γ-ray irradiation on Chinese hamster ovary (CHO) cells. They observed that fractionated neutron-irradiation reduced the number of 53BP1 foci by 25% while significantly increasing the focal volume. This indicated that the repair of high-LET-induced clustered DSBs was challenging and led to the accumulation of unrepaired damage. Conversely, the number of foci decreased by 30% after fractionated γ-irradiation, although the volume did not change, demonstrating the possibility of partially repairing sublethal damages due to low-LET radiation. Dissimilar to BNCT-induced complex DSBs that hinder the binding of repair proteins (e.g., Ku70/80 and DNA-PKcs) and follow the inefficient NHEJ and complicated HRR pathway owing to the lack of an intact template (ultimately triggering the cell-cycle blockade or death program), low-LET radiation-induced isolated DSBs can be repaired via NHEJ or rapidly repaired by HRR. In Ku80-deficient CHO cells, BNCT has induced high cytotoxicity, and irradiated cells have demonstrated inefficiency in repairing DSBs 111. BNCT-induced DNA-repair pathways differ across cancer-cell lines. While the HRR pathway is activated in thyroid cancer cells 112, HRR and NHEJ are activated in melanoma cells. Chen *et al.* further demonstrated that the HRR pathway mainly accounts for the repair of HCC cells 113.

Thus, the detection of early and late biomarkers for BNCT can facilitate the biological evaluation of boron-delivery agents or the efficacy of BNCT alone or combined with other therapeutic strategies, thus optimizing the conditions for BNCT.

When cells are exposed to ionizing radiation (IR), they exhibit a variety of responses depending on the type of ionizing radiation, LET, dose and cell type. The mechanisms by which radiation induces tumor cell death have been extensively studied; thus, ionizing radiation induces cell death through apoptosis, autophagy or necrosis, mitotic catastrophe (MC), and senescence, which may occur simultaneously. However, when DNA damage is repairable, only a transient growth arrest occurs, which eventually leads to DNA repair, cell cycle continuation, and restoration of cell growth. However, once a cell experiences severe DNA damage (e.g., complex DNA damage), prolonged DDR signaling is triggered. Mitotic catastrophe and apoptosis are the more common outcomes for most cell types. P53 accumulation plays a vital role as one of the targets of ATM/ATR when radiation induces cell death. p53 as The most commonly mutated tumor suppressor gene, regulates genes that control both cell-cycle checkpoints and programmed cell death via apoptosis 104. It plays a critical role in the DNA damage response of cells. When DNA damage cannot be repaired, it causes overexpression of P53. p53 activated by ATM/ATR will initiate the apoptotic pathway, thus delaying the G1 phase of the cell, and more and more experiments have shown that there is a correlation between the status of P53 and cytotoxicity of high LET. In tumor cells, P53 is mutated, and several studies have reported that cells expressing mutant P53 usually have reduced sensitivity to radiotherapy 114. Yusei Fujita's team 115 has demonstrated this by comparing p53 wild-type (SAS/ neo) versus mutant (SAS/ mp53) oral squamous carcinoma cells in terms of treatment response, the effect of mutation was further elucidated by experimental data: wild-type p53 cells were significantly less viable than mutant-type and had more pronounced proliferation inhibition at the same physical dose.

**Figure 20:**
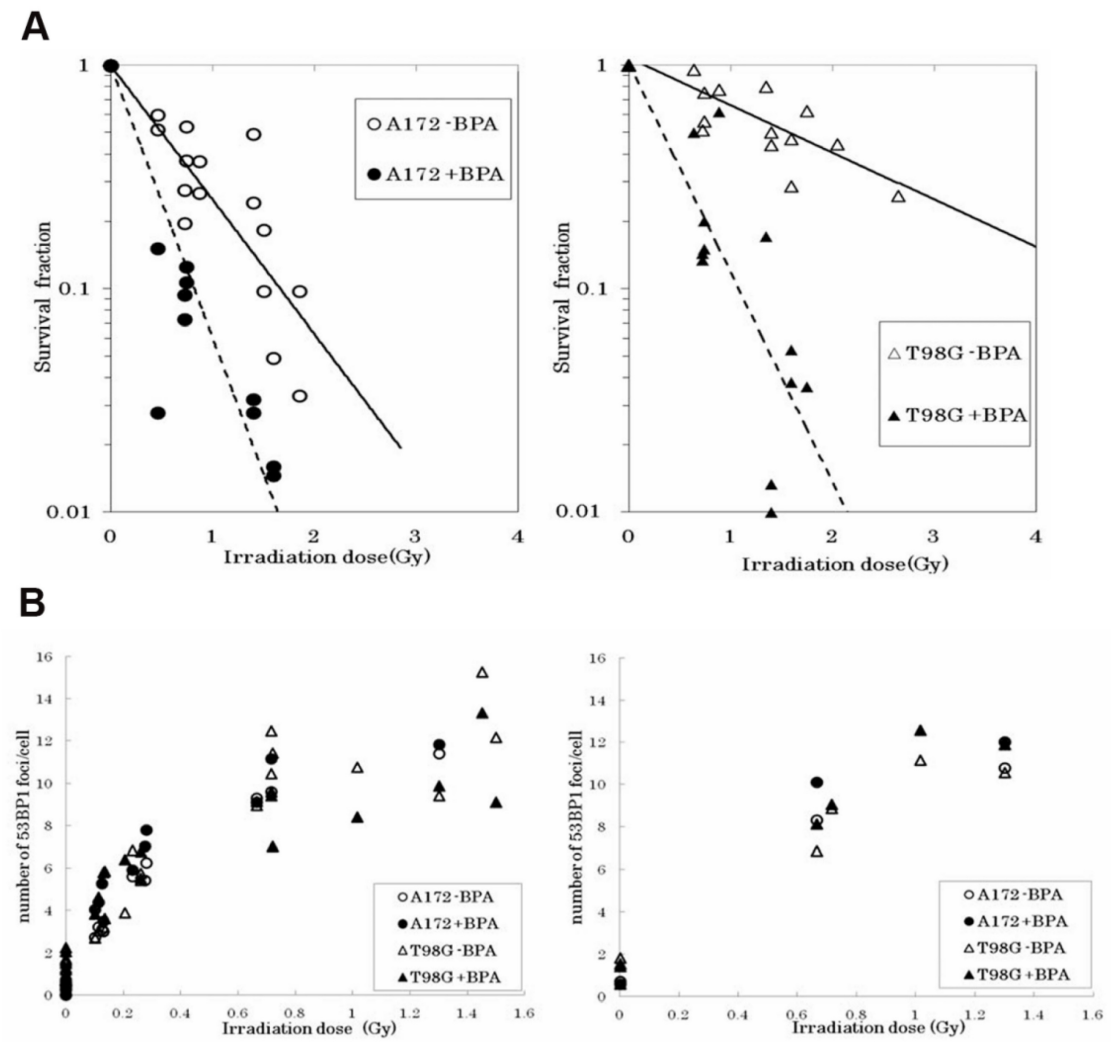
** (A)** Survival fraction of irradiated A172 and T98G cells by mixed-neutron beam for BNCT. **(B)** Number of 53BP1 foci 1 and 3 hours after the irradiation. The right graph shows the number of foci 1 hour after irradiation, and the left graph shows the number of foci 3 hours after irradiation. Each data point represents the average number of foci in more than 50 cells. Reproduced with permission from Ref. 104. Copyright 2015, High Wire.

This confirms that functional p53 enhances BNCT sensitivity by activating G1 phase blockade and early apoptosis. Brain tumors, as a hotspot of BNCT research, have been focused on by many researchers. Seki et al. 104 revealed the mechanism by which p53 status regulates the therapeutic efficacy by comparing the response of wild-type (A172) versus mutant p53 (T98G) glioblastoma cells to BNCT. It was found that T98G cells exhibited significant radio resistance compared to A172 without the boron carrier BPA, but the survival curves of the two converged after the addition of BPA, suggesting that BPA enhances the response to BNCT through the enhancement of ^10^B (n, α)^7^ Li reaction with a high LET particle killing effect, overcoming the resistance caused by the p53 mutation (Figure 20A). Although there was no significant difference in the number of DNA damage marker 53BP1 foci between the two cells (Figure 20B), the apoptosis rate of T98G cells was remarkably lower than that of A172, suggesting that the p53 mutation inhibited the programmed death pathway. However, BNCT combined with BPA was still effective in inducing apoptosis in T98G cells, suggesting that it exerts its efficacy through p53-independent mechanisms such as mitotic catastrophe or necrosis.

MC, also known as mitotic death, has been expressed in a variety of ways. Initially, MC was assumed to be associated with incomplete DNA synthesis and chromosome condensation, exhibiting the same characteristics as apoptosis. Therefore, most researchers believe that MC represents the pre-apoptotic or necrotic stage of cell death. Mitotic death is a delayed response in a p53 mutant that is resistant to genotoxic damage. To maintain genomic integrity during DNA damage, cells are typically removed by decelerating cell-cycle progression or by removing damaged, irreparable cells. Once the DNA damage-detection site is compromised, the cell may advance into mitosis and initiate MC before completing DNA repair. Other researchers have defined MC as a form of mitosis exhibiting an aberrant form. When MC occurs, it generally presents morphological changes, including the generation of micronuclei and multinuclei. Micronuclei are mainly produced by the failure of chromosomes to be evenly distributed to daughter nuclei, whereas multinuclei are two or more similarly sized or unevenly distributed nuclei resulting from abnormal division during cytoplasmic division. Fujita *et al.* 116 revealed a unique cytotoxicity mechanism that involves tumor-cell killing using BNCT. This is achieved by inducing MC based on the effects of BNCT on p53-mutant oral squamous carcinoma (SAS/mp53) xenograft tumors. When treated with BNCT, mutant p53 tumors exhibited multinucleated giant cells accompanied by abnormal chromosome cohesion, nuclear segmentation, and intracellular vacuolization within 6h. However, apoptosis and necrosis predominated in the wild-type p53 tumors (SAS/neo). For the first time, we clarified these molecular mechanisms. Additionally, the investigators observed that although BNCT inhibited tumor growth within two weeks, the recurrence rate of mutant p53 tumors was significantly higher, indicating that the multinucleated cells may have escaped death through aberrant proliferation.

To increase the RBE of BNCT, proteins of the DNA-repair pathway can be specifically targeted to impede the ability of a tumor to repair IR-induced damage. However, identifying a uniform target using the IR pattern is challenging because the DNA-repair pathway varies among cancer types and under different radiation conditions. Despite these drawbacks, numerous studies demonstrate the effectiveness of targeting DSB-repair proteins (e.g., DNA-PKcs and PARP-1). Overall, BNCT may have emerged as the safest method for delivering high-LET IR to cancer cells, as well as inducing complex lesions in their DNAs that are not readily reparable.

## 4. Visual evaluation of boron neutron-capture therapy

**Figure 21:**
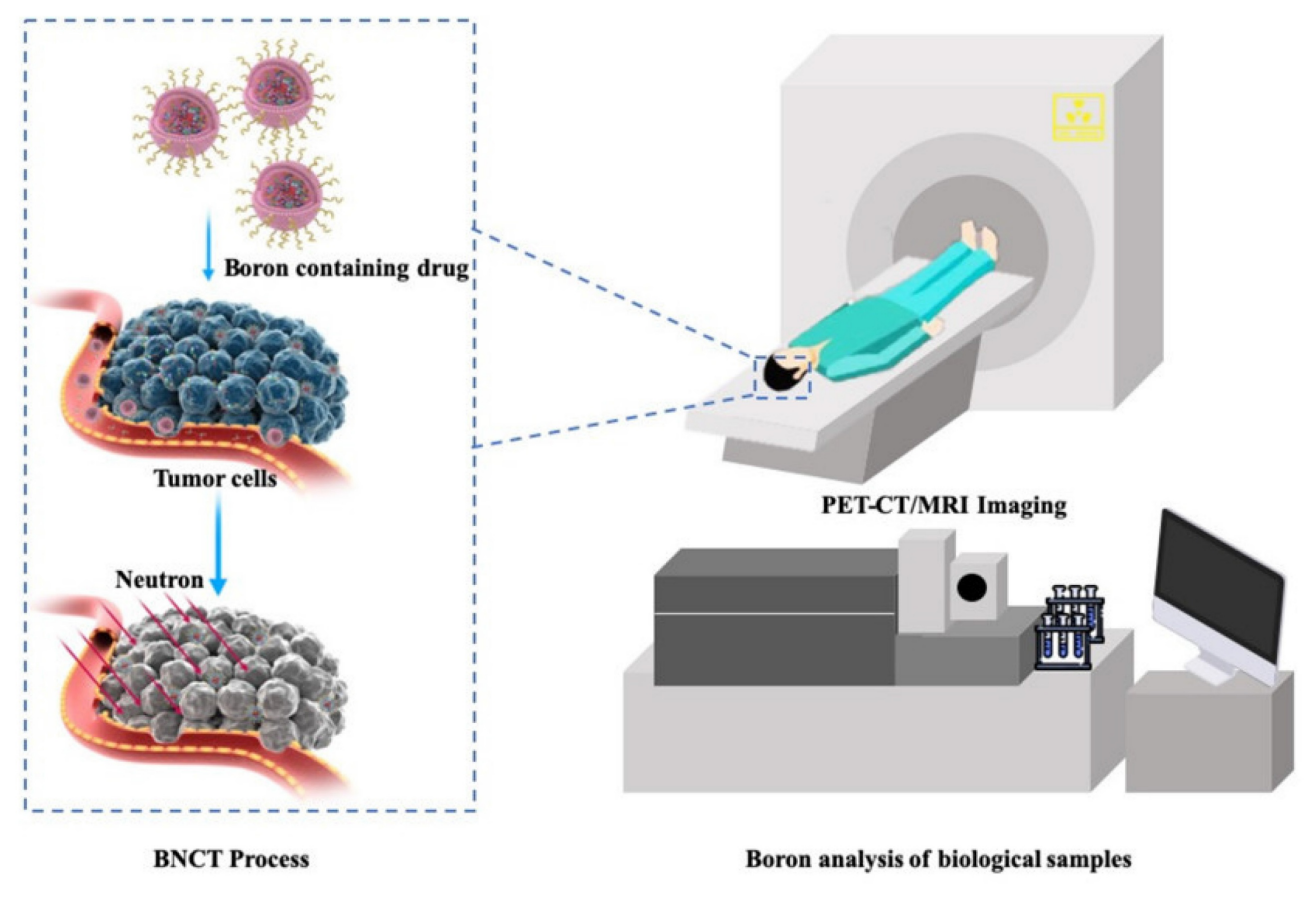
BNCT imaging and content testing. Reproduced with permission from Ref. 117. Copyright 2022, ACS.

Clinical BNCT comprises two main processes: (1) delivery of targeted boron to the tumor, and (2) irradiation of the tumor with a high-intensity neutron beam when the T/N of ^10^B concentration reaches a peak 117. Neutron irradiation exerts complex effects and drives dose distributions in tumor tissues, with the main dose-distribution stemming from the ^10^B (n, α)^7^Li reaction. The reaction range of these particles is typically very small (5-9 μm, which is equal to the diameter of the cell) and can achieve the targeted irradiation of individual tumor cells. Therefore, the clinical efficacy of BNCT can only be achieved using an appropriate boron concentration and at an optimal location in the tumor tissue. In conclusion, to improve the clinical efficacy of BNCT, a boron agent must be used as the precursor, and the radiation dose must also be determined, as it plays a crucial role in BNCT. Currently, the limitations regarding the radiation dose for BNCT mainly stem from the inability to accurately measure the boron concentration of tumors in real time. This has highlighted the need for intuitive, real-time measurements of the T/B boron concentrations, as well as cellular- or subcellular-level microspatial distribution. Such real-time, precise measurements can further enable precise control over the optimal timing of treatment. Currently, imaging is mainly accomplished by various instruments to determine boron distribution and measurement (Figure 21).

### 4.1 Macroscopic imaging techniques

The main macroscopic imaging methods for in vivo boron analysis include computed tomography (CT) and magnetic resonance imaging (MRI). Historically, X-ray CT was the first imaging modality that enabled the accurate, non-destructive reconstruction of the internal image of an object using a sufficient number of X-ray projections. CT investigation is facile and fast, exhibiting high resolution. MRI mainly uses electromagnetic waves emitted during magnetic resonance to form images. These waves and their related parameters are subsequently converted using spatial coding technology, after which they are processed by electronic computers and finally generated as images for accurate diagnosis. Both techniques offer good observation conditions for tumor distribution, although they do not provide information about drug metabolism in the tissues.

#### 4.1.1 Positron emission tomography combined with computed tomography

PET has emerged as one of the most valuable tools in clinical practice and biomedical research. It is used for visualizing and evaluating several biochemical processes in the body. The ability to label specific molecules with radioisotopes (e.g., ^11^C and ^18^F) has enabled the imaging of specific organ structures based on their metabolic activity. This has transformed the detection mode from early disease localization to more effective measurements, marking a new era of PET-based molecular imaging.

**Figure 22:**
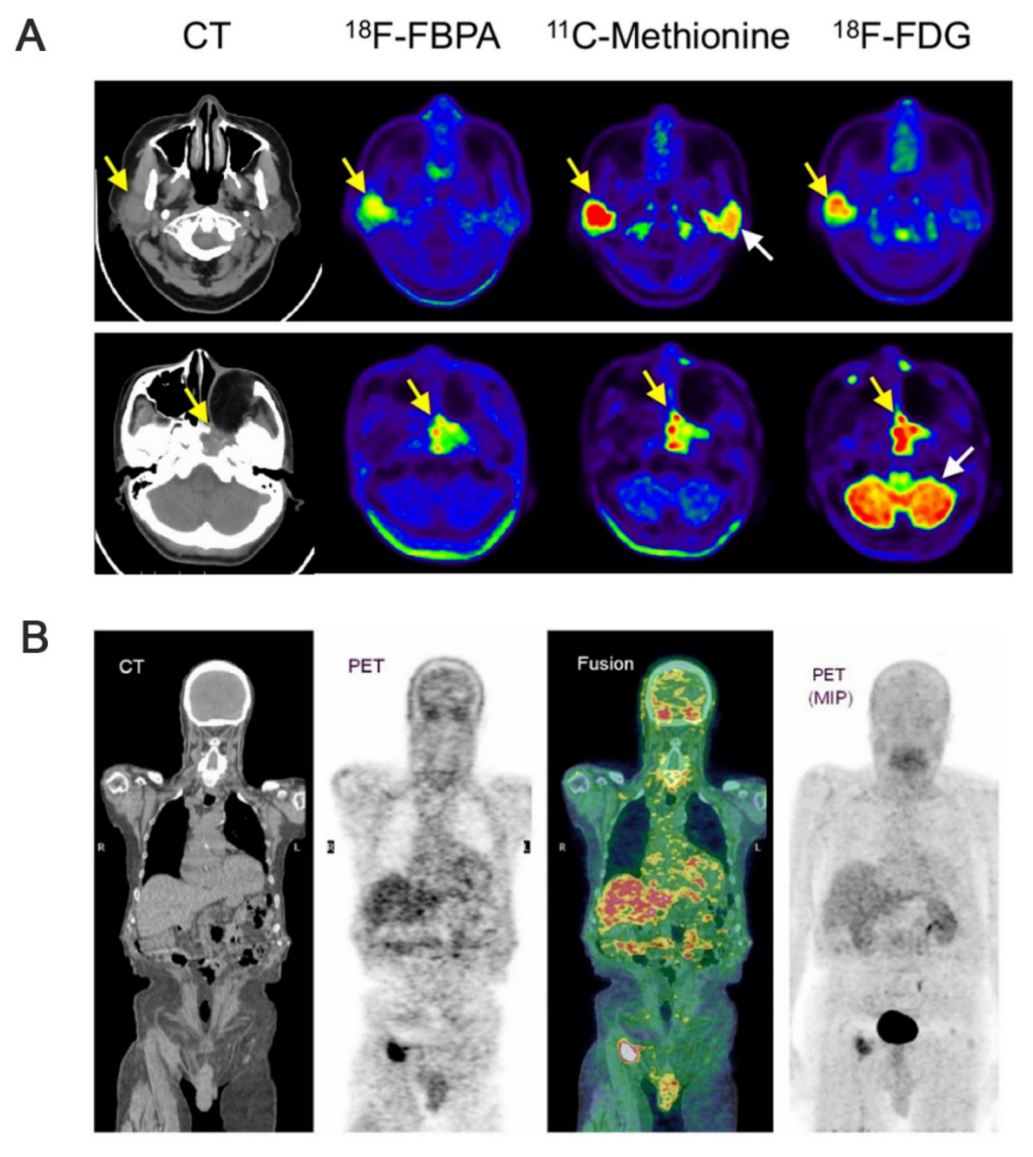
** (A)** PET/CT Images of Patients with Ductal Carcinoma (Upper Row) and Squamous Cell Carcinoma (Lower Row) of Salivary Glands Injected with F-FBPA and Other Radiation Agents. Reproduced with permission from Ref. 119. Copyright 2019, Springer Nature. **(B)** Coronal [^18^F] FBPA PET/CT images of a patient with melanoma in right ankle (not shown) metastatic to right inguinal lymph node (visible in all images). Reproduced with permission from Ref. 120. Copyright 2009, Elsevier.

In 1991, Ishiwata *et al.* 118 synthesized ¹⁸F-labeled BPA ([¹⁸F]BPA) by the direct fluorination of BPA. In the 2000s, marked by technological advances, a high-spatial-resolution PET scanner, which had been developed for small animals, was used for the PET imaging of [^18^F]FBPA in animal models 119. The metabolic properties and in vivo imaging-based monitoring capabilities of [^18^F]FBPA make it an ideal tracer for the real-time monitoring of the boron concentrations of tumors during BNCT (Figure 22A). Clinically, the estimated boron concentrations of tumors based on the kinetic parameters of dynamic [^18^F]FBPA PET are similar to those observed during the continuous injection of ^10^B-BPA. This validates the utilization of [^18^F]FBPA PET for ^10^B-concentration estimation. Thus, PET imaging has been used to quantitatively assess boron concentration and dynamic changes, facilitating its uptake in tumors.

Conventional BNCT lacks a real-time, noninvasive means of monitoring boron concentration and selective BPA accumulation in tumors and normal tissues. Thus, Menichetti *et al.* 120 used PET to quantify the BPA concentration of a tumor. They achieved this by combining the tracer, ^18^F, with BPA to form [^18^F]BPA. Thereafter, they quantified the BPA concentration of the tumor by combining pharmacokinetic modeling with dynamic imaging parameters, including PET uptake rate (K1), retention time, and boron distribution (Figure 22B). This combination also allowed for an improved estimation of the T/N BPA concentration ratio. Based on the concentration ratio for each patient, a decision can be made as to whether BNCT would be advantageous owing to the selective accumulation of BPA in individual tumors. Clinical data from this study indicated that [^18^F]BPA exhibited high T/N uptake ratios (>3) in glioblastoma, head and neck malignancies, and metastatic melanoma, and correlated well with postoperative boron-concentration measurements.

This technology can accurately identify complex tumor foci, such as brain malignancies, and determine the T/N during treatment, thus contributing to the further improvement of the treatment effect of BNCT.

#### 4.1.2 Magnetic resonance imaging

First, natural boron isotopes, ^10^B and ^11^B, can be detected by nuclear magnetic resonance (NMR). This property allows MRI to directly detect the in vivo distribution of boron carriers, such as BPA and BSH. The relative spin-to-magnetic ratio (γ) of ^10^B is relatively low (0.107) compared with that of ^11^B (0.32) 121. Although ¹⁰B exhibits a higher γ, it exhibits the unique relaxation property of spin 3, which gives it an advantage of having a longer T₂ for the same molecular position. T₂ refers to the transverse relaxation time, which indicates how fast the magnetization intensity decays in the transverse plane, and a longer T₂ means that the signal decays more slowly. During MRI, signal acquisition must be completed within the echo time (TE), and a shorter T_2_ means that the signal may have completely decayed before the sample is imaged using in vivo boron MRI. A longer T₂ indicates that the allowable TE can also be longer, providing more time for signal acquisition and potentially improving the signal-to-noise ratio. Conversely, when T₂ is short, a very short TE must be used to avoid the rapid decay of the signal, resulting in a weak or lost signal. A shorter TE may require faster imaging techniques, which may limit the resolution of the resulting image.

**Figure 23:**
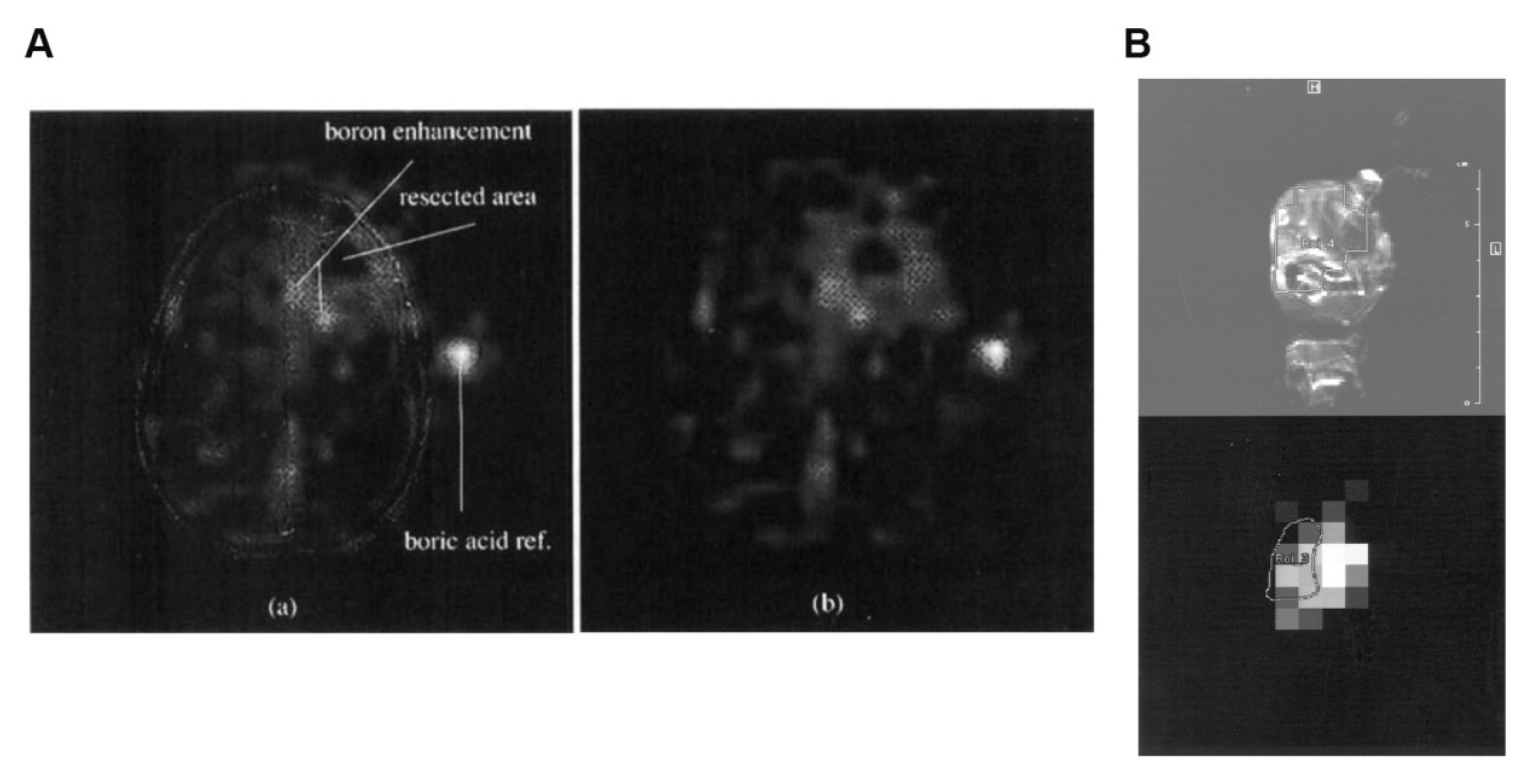
** (A)** Scan of the tumor area obtained 5 minutes after termination of input BSH. Reproduced with permission from Ref. 123. Copyright 1995, Wiley. **(B)** ^1^H (top) and ^10^B (bottom) images of mice with implanted tumors. Reproduced with permission from Ref. 124. Copyright 2001, Wiley.

A shorter relaxation time of ^10^B in biological environments limits ^11^B NMR detection to methods that are suitable for MRI detection. To resolve this issue, researchers have adopted 3D imaging techniques rather than slice-selective imaging techniques and avoided conventional phase-encoding. In the early days, back-projection, mainly attempted on rats, or single-point imaging was employed for imaging. However, the only human ^11^B MRI was performed by Bradshaw and co-workers using a special 3D back-projection scheme developed by Glover *et al.* 122. Unfortunately, the image resolution consists primarily of a variety of 3D data. Employing BSH as the boron-delivery agent, the images were acquired 5 min after a 60 min intravenous injection of the drug. The BSH imaging revealed residual active-tumor areas in the peripheral region of a surgically resected glioblastoma multiforme, and this enabled the identification of the precise target area for BNCT (Figure 23A) 123. However, the low nuclide density would weaken the MRI of ^11^B, limiting its application in the imaging guidance of BNCT.

Thus, ^10^B NMR is the most direct approach for detecting ^10^B-rich molecules. BSH and BPA, two conventionally deployed boron-delivery agents in the clinic, can also be detected by MRI. Beginning from 2001, Bendel and his team 124 performed the first noninvasive imaging of the BNCT drug, BSH, in living mice via ^10^B MRI. By optimizing the 3D gradient echo, they detected a boron concentration of approximately 20 ppm (μg boron/g tissue) in the tumor region with a 6 × 6 × 6 mm^3^ spatial resolution in 16 min. By achieving this, they overcame the low ^10^B γ challenge and validated the applicability of ^10^B MRI.

However, for BPA, detecting the molecule based on the signal of some hydrogen atoms in the molecule, i.e., proton NMR (^1^H NMR), is more appropriate than direct ^10^B NMR. Owing to the low sensitivity and high background interference associated with the direct detection of traditional boron isotopes (^10^B and ^11^B), researchers 125 have explored an alternative approach. By leveraging the noninvasive method, ^1^H NMR, they verified the feasibility of specifically detecting BPA in blood, brain tissue, and tumors. This was accomplished by analyzing the unique aromatic-proton signals (7.1-7.4 ppm) in the BPA molecule at a high field strength of 8.4 T. They demonstrated that the aromatic bimodal signal of BPA maintains a linear response in complex biological environments without any significant overlap with endogenous gmetabolites (e.g., lipids and tyrosine), as shown in Figure 23B. Based on this, Bendel and his team 126 further optimized ^1^H NMR and reported, for the first time, the noninvasive imaging of BPA in animals using ^1^H NMR.

**Figure 24:**
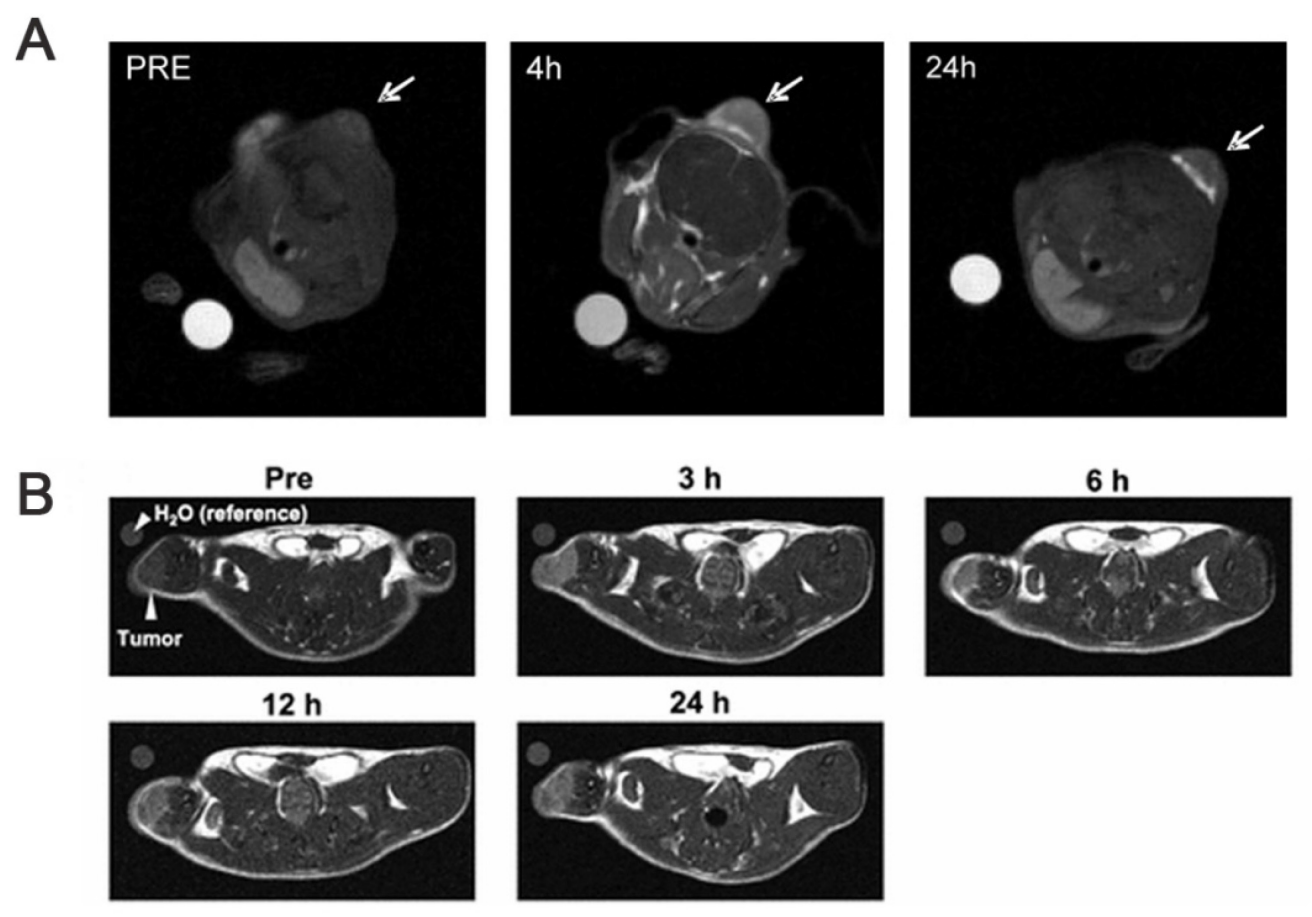
** (A)** MRI “in vivo” in B16 tumour-bearing mice after administration of the Gd/B/L particles. Reproduced with permission from Ref. 131. Copyright 2011, Wiley. **(B)** Imaging of tumor-bearing mice injected with Gd-MID-BSA. Reproduced with permission from Ref. 132. Copyright 2023, ACS.

Additionally, Gd has garnered increased attention as a complementary or alternative to boron-based BNCT owing to its high capture efficiency. Notably, ^157^Gd exhibits a thermal-neutron-capture cross-section of up to 255,000 barn, which is much higher than that of ^10^B (3837 barn). This high neutron-capture cross-section significantly increases its uptake efficiency for the same neutron flux 127. After neutron capture, ^157^Gd mainly releases γ-rays and internally converted electrons. The long-range γ-rays can cover larger tumor areas, whereas the low-energy electrons, with a range of only micrometers, can cause intensive ionization damage near DNA. Thus, boron-delivery agents have been combined with Gd to enhance BNCT 128. Additionally, Gd-based compounds are often utilized as MRI contrast agents in clinical settings and can further enhance BNCT when guided by MRI. The most conventional clinical MRI contrast agent is a complex comprising a Gd ion and diethylenetriaminepentaacetic acid (DTPA), Gd-DTPA. Yamamoto and colleagues synthesized two Gd-DTPA complexes, which were functionalized with CB 129 and BPA 130. The BPA-functionalized complex (BPA-Gd-DTPA) accumulated more in tumor cells than the CB-functionalized one. However, when Gd-DTPA was coupled with BPA, the biodistribution and intra-tumor concentration of the boron atoms in BPA-Gd-DTPA differed significantly from those of BPA alone. Simonetta 131 addressed this issue by constructing an innovative Gd/B bifunctional probe for MRI-guided BNCT based on a low-density lipoprotein (LDL) carrier (Gd/B/L-LDL) (Figure 24A). The study achieved targeted delivery by combining a CB unit containing 10 boron atoms with a gadolinium complex (Gd-DOTA) and exploiting the overexpression of the LDL receptor in tumor cells. The follow-up in vitro experiments indicated that the efficient enrichment of the probe by receptor-mediated endocytosis occurred in human HCC (HepG2), mouse melanoma (B16), and human glioblastoma (U87) cells. The boron concentrations were as high as 34 μg/g, and MRI-signal enhancement correlated well with the boron concentrations determined by ICP mass spectrometry (MS). To further explore imaging and targeting, Okada's team 132 developed a novel Gd-B conjugated bovine serum albumin (Gd-MID-BSA), which serves the dual function of tumor localization and neutron-capture therapy using Gd-DTPA as an MRI contrast agent. Targeted enrichment was achieved via the EPR effect, which ensured high penetration and long retention. The timing of treatment was guided in real time by dynamic MRI monitoring combined with synergistic tumor-cell killing by the γ-rays and ɑparticles from Gd and boron, respectively (Figure 24B).

### 4.2 Boron microimaging

The efficacy of BNCT is closely related to the in vivo concentration of boron. Therefore, it is very crucial to obtain the subcellular-level microdistributions of ^10^B in tumor and normal tissues and cells. The existing boron microimaging methods include neutron-capture radiography (NCR), secondary ion MS (SIMS), and electron energy-loss spectroscopy (EELS). Their spatial resolutions can reach the micrometer level (μm). Moreover, they can detect boron at the subcellular level.

#### 4.2.1 Neutron-capture radiography

In the early days of BNCT, researchers used organic, homogeneous, solid-state detectors to analyze biological samples. This was accomplished by detecting fragments that were produced by ^10^B during neutron-capture reactions and BNCT for tumor-bearing mice. Such fragments were subsequently used to generate the neutron-capture radiographic images of the whole-body tissue sections of the mice. This technique is simply known as NCR. For example, high-sensitivity detection of boron markers has been achieved with excellent spatial resolution and significantly reduced background interference. This was accomplished by covalently conjugating ^10^B to macromolecular carriers, such as immunoglobulins and dextran, as well as by combining a cold neutron beam with a solid-state ^4^He^2+^ detector (e.g., a nitrocellulose membrane) 133.

**Figure 25:**
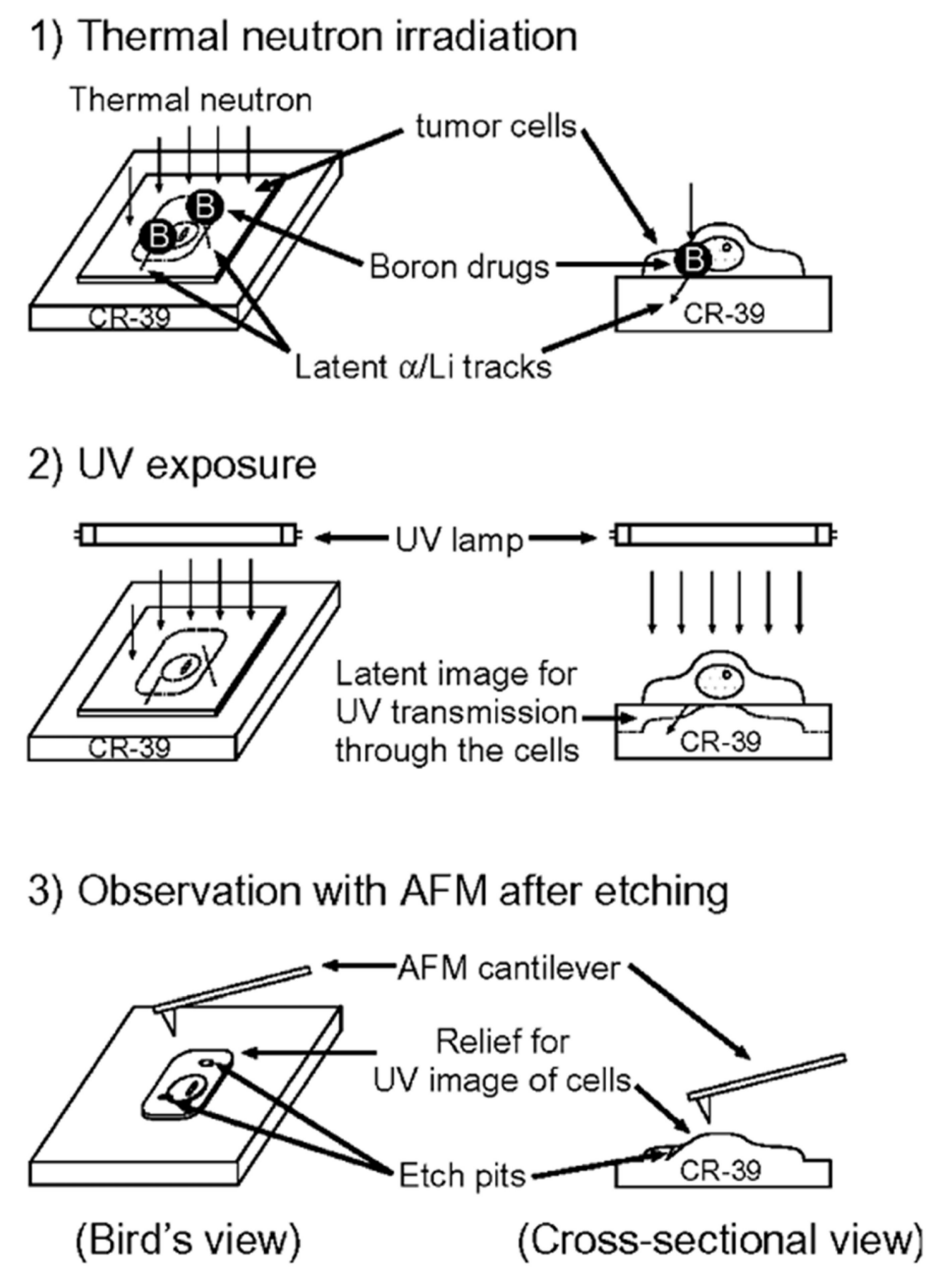
Schematic flow of the high-resolution neutron-induced autoradiography with the contact UV microscopy technique. Reproduced with permission from Ref. 134. Copyright 2005, Elsevier.

Such techniques have evolved with time, and high-resolution neutron-induced α-radiation autoradiography has emerged. This technique can be combined with atomic force microscopy (AFM) for greater precision. Amemiya *et al.* 134 applied cold neutron beams to induce the release of ^4^He^2+^/^7^Li^3+^ from boron compounds (BSH or BPA). This was followed by UV exposure and chemical etching coupled with AFM observations. Overall, they achieved subcellular-level radial-trace imaging with a sub-100 nm resolution (Figure 25).

Altieri *et al.* 135 demonstrated a method for recording the ^4^He^2+^/^7^Li^3+^ particle trails released from boron compounds (e.g., BPA) under thermal-neutron irradiation. This method combines cryosections with CR-39 or CN85 plastic detectors to visualize the spatial distribution of boron concentration, which is achieved by chemical etching, followed by microimaging. The method distinguished between tumor-active areas, necrotic areas, and normal liver tissues in liver metastasis samples, revealing that tiny metastatic foci still retained significant boron selectivity. Additionally, the study validated the feasibility of the method for rapid imaging (<5 min), providing technical support for the real-time analysis of clinical samples.

#### 4.2.2 Secondary ion mass spectrometry

**Figure 26:**
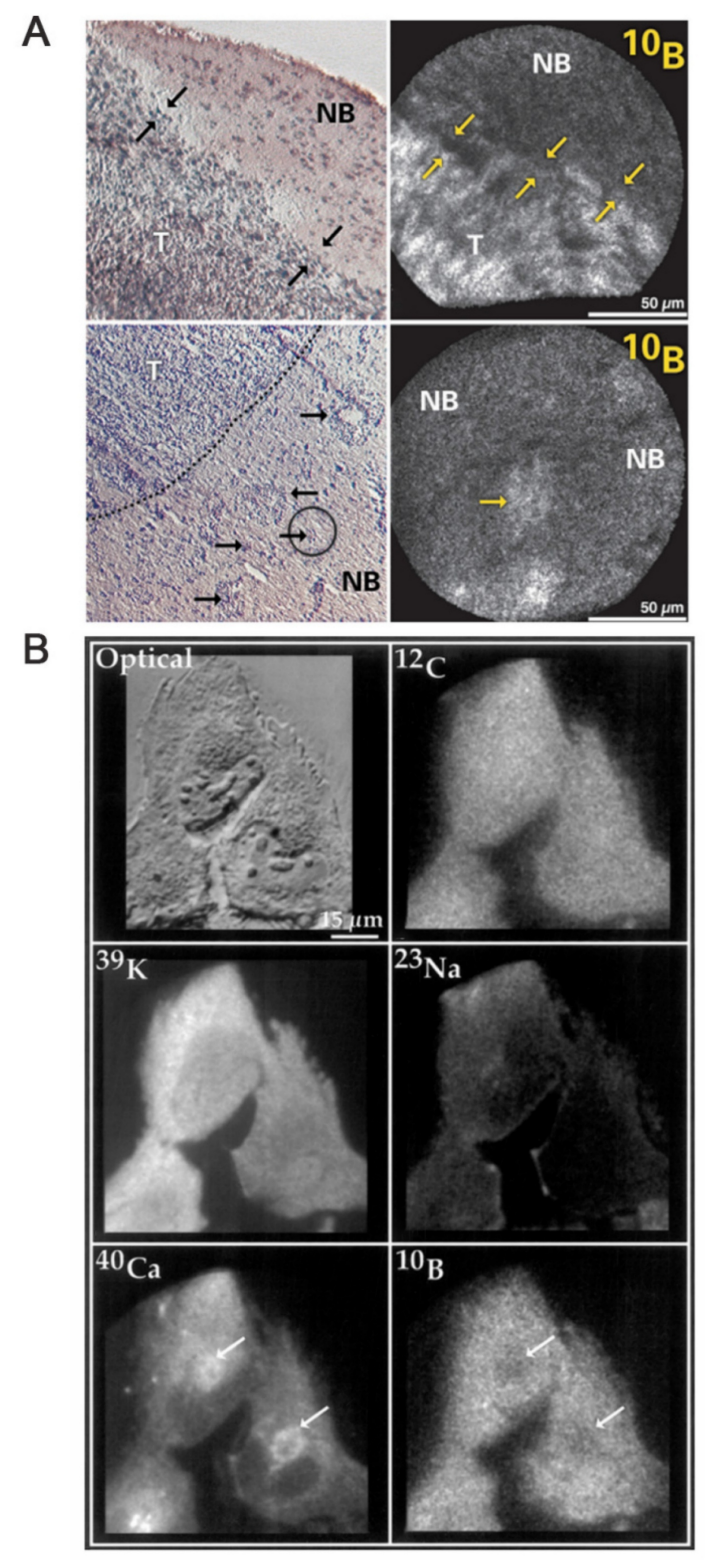
** (A)** SIMS imaging of ^10^B from BPA in a 9L gliosarcoma rat brain tumor model. Reproduced with permission from Ref. 136. Copyright 2000, ACS. **(B)** SIMS isotope images of T98G human glioblastoma cells after a 1-h exposure to 110 g/ml boron equivalent of ^10^BPA-F. Reproduced with permission from Ref. 137. Copyright 2002, BioOne.

SIMS is based on the principle that a focused, high-energy primary ion beam excites the sample surface, generating a few secondary ions, which are subsequently analyzed by MS. Delivering a spatial resolution of less than 10 μm, SIMS can provide subcellular-level quantitative and qualitative information. Chandra *et al.* 136 optimized the sample-preparation process by freeze-drying and other methods. This approach solved the issue of traditional chemical fixation, altering the natural chemical composition of a sample. Further, by preserving the integrity of the sample, they achieved high-resolution analysis of the distribution of ions and molecules within the cell. Subsequently, they processed BPA using the already optimized method and applied it to a glioma rat model. Through SIMS analysis, they detected BPA concentrations in the tumor region that were more than three times higher than those in normal brain tissues. This analysis, which was performed at sub-micron resolution, identified the boron-selective enrichment of the infiltrating tumor-cell clusters for the first time (Figure 26A). In 2002, Chandra's team 137 also employed SIMS as a subcellular-level imaging tool for the precise analysis of the distribution of a BPA-fructose complex (BPA-F) and BSH in T98G glioblastoma cells (Figure 26B). The experiments demonstrated high-resolution, independent imaging of two boron isotopes in the same cell. This was achieved by labeling ^10^B with BPA-F and labeling ^11^B with BSH. The study revealed the distribution patterns of the drugs under independent and combined dosing.

With the rapid development of science and technology, more advanced SIMS systems have been introduced, such as time-of-flight SIMS (ToF-SIMS), 3D orbitrap SIMS, and nanoscale SIMS (Nano-SIMS). These systems, particularly Nano-SIMS and ToF-SIMS, are among the highest spatial-resolution MS imaging techniques available for research and have been applied in medical analysis and other research fields. For example, Nano-SIMS exhibits a lateral resolution of up to 50 nm, making these systems highly effective for boron imaging in BNCT.

#### 4.2.3 Electron energy-loss spectroscopy

Electron energy-loss spectroscopy imaging for boron detection in biological cryofixed tissues: EELS measures the characteristic energy loss of electrons as they penetrate a sample. As different elements exhibit unique energy-loss values, EELS provides a means of measuring elements (e.g., B and C). When combined with transmission electron microscopy (TEM) or scanning TEM, it can achieve high spatial resolution. Michel's team first proposed an EELS-based method for detecting boron in freeze-dried sections of human melanoma cells and further extended this approach from cell to tissue evaluation. The study enabled the analysis of subcellular localization of BSH and BPA in the kidney, liver, and tumor tissues of a mouse by implementing the freeze-drying technique with the spectral imaging mode 138. In addition to the subcellular distribution of boron drugs, researchers have also explored the limit of detection obtainable by EELS. Malac's team 139 performed a quantitative analysis of boron concentration by combining high-resolution TEM (HRTEM) with EELS. They used three types of samples for their experiments: a homogeneous boron layer, a non-homogeneous evaporated boron sample, and BPA-F-labeled tobacco mosaic virus.

Under an electron dose of 0.1 C/cm², the experiments achieved a boron-detection sensitivity of up to 0.2% (2000 ppm) with 10% accuracy. It also obtained spatial-distribution imaging resolution of up to 66 nm pixels, with the ability to detect the lowest concentration of 0.5% (5000 ppm). These results provide crucial technical parameters for analyzing the subcellular distribution of boron drugs in BNCT and for optimizing the radiation dose.

For BNCT, an accurate understanding of the boron distribution and concentration within the body is very critical to fully maximizing the advantages of BNCT. Notably, the therapeutic dose of boron can only be effectively controlled when its biodistribution and pharmacokinetics are accurately understood. As mentioned above, while SIMS and EELS can map the spatial distribution of boron with subcellular resolution, practical limitations prevent them from meeting the current detection needs. Similarly, while PET and MRI have attracted considerable attention in BNCT for noninvasive boron-distribution imaging, both macroscopic measurement techniques have their drawbacks. In recent years marked by the rapid development of integrated systems combining nanomaterials with boron drugs, many researchers have explored new possibilities. Many are now attempting to equip these nanomaterials with imaging capabilities. As exemplified by the combination of Gd with BNCT, the future of BNCT imaging will be driven by integrating different techniques to overcome current constraints.

## 5. Experimental device for accelerator-based boron neutron-capture therapy

BNCT is a novel cancer treatment that can achieve cellular-level targeting with minimal damage to surrounding healthy tissues. It is a promising procedure for treating refractory and recurrent cancers. Based on the neutron source employed, BNCT can be categorized as reactor-based BNCT or AB-BNCT 15. Reactor-based BNCT utilizes nuclear-reactor-generated thermal neutrons for therapy, although it is limited by high costs and large space requirements. Conversely, AB-BNCT utilizes accelerators to produce high-thermal-neutron beams. Owing to their cost-effectiveness, high efficiency, and operational flexibility, AB-BNCT systems can be readily installed in hospitals, thereby enabling in-hospital cancer treatment. Consequently, AB-BNCT has gradually emerged as the dominant trend in BNCT technology, providing a more precise and reliable solution for cancer therapy 140.

### 5.1 Core technologies of accelerator-based boron neutron capture therapy

BNCT systems consist of three core modules: accelerator/neutron source, beam-shaping assembly (BSA), and beam delivery and patient-positioning system. Among them, accelerator technologies are primarily classified as electrostatic (ES) or radiofrequency (RF) accelerators based on the principles of particle acceleration. RF accelerators are further subdivided into linear accelerators (LINACs) and cyclotrons. Additionally, synchrotrons and compact deuterium-tritium or deuterium-deuterium (D-T/D-D) neutron generators have emerged as supplementary technologies with specific application potential.

#### 5.1.1 Electrostatic Accelerators

The evolutionary trajectory of ES accelerators can be traced to the Van de Graaff generator, which was pioneered by Robert J. Van de Graaff in 1930. This accelerator is based on the fundamental principle of directly accelerating charged particles via high-voltage electric fields. Modern iterations, such as the Tandem ES (TES) accelerator, use multi-stage configurations to increase proton energies to the 5-10 MeV range. Although these systems exhibit great potential for particle-acceleration applications owing to their structural simplicity and stable energy output, they are inherently limited by the breakdown or dielectric strength of insulating materials, which results in the ongoing drawback of low neutron yields. One example is the TES quadrupole (TESQ) accelerator facility developed by the National Atomic Energy Commission (CNEA) in Buenos Aires, Argentina. TESQ optimizes the performance of the neutron source to generate high precision, stable neutron beams, thus improving the efficacy and safety of cancer-targeted BNCT 141.

#### 5.1.2 Radiofrequency accelerators

The pivotal advancement of RF accelerators is traceable to the groundbreaking research at the Los Alamos National Laboratory in the 1980s. This research demonstrated the simultaneous control of particle focusing and acceleration using high-frequency electromagnetic fields. These fields were optimized for low-to-medium energy particle beams (2-5 MeV) employed in medical applications. An example is a 2.45 GHz RF quadrupole (RFQ) device that was developed by Kyoto University. This compact design achieves stable proton energies of 2.5 MeV for clinical head and neck cancer therapies. However, its low-energy threshold necessitates the utilization of specially enriched target materials to compensate for the resulting limitations in neutron yield.

The cyclotron is a foundational apparatus in nuclear physics, which was pioneered by Ernest Lawrence in the 1930s. This apparatus functions by exploiting the interaction between oscillating electric fields and static magnetic fields. This interaction enables the acceleration of charged particles in a continuous spiral motion. Modern advancements in superconducting magnet technology have contributed to its significantly reduced energy consumption while elevating its beam intensities to the 10-30 MeV range. Examples include a system developed by Sumitomo Heavy Industries (Japan), which achieves a clinical-grade neutron flux, and a device developed in Finland, which enhances hospital compatibility through its modular design. However, mandatory radiation-shielding infrastructure and the high maintenance costs associated with these systems remain primary constraints to their large-scale clinical adoption. The State Key Laboratory of Nuclear Physics and Technology at Peking University has designed a 162.5 MHz continuous-wave RFQ accelerator with a transmission efficiency of 98.63%. This accelerator accelerated a 10 mA deuteron beam from 0.04 to 2.1 MeV. Subsequent comprehensive multiphysics simulations were performed to validate the reliability of its electromagnetic configuration, thermal management, and vacuum-system design 142.

LINACs employ a multi-stage RF cavity acceleration strategy to generate continuous beams of 10 MeV-GeV-level energies. Originally developed for high-energy physics research in the 1950s, this technology has progressively expanded to medical applications. Notably, Sweden's Skandion Clinic is currently investigating the integration of LINACs with neutron-producing targets to validate the feasibility of implementing BNCT. However, challenges, such as the large physical footprint of these systems and substantial operational costs, must be further optimized to enhance clinical viability.

#### 5.1.3 Exploration of other accelerators

Synchrotrons achieve GeV-scale ultrahigh-energy particle acceleration through toroidal magnetic field confinement and synchronized electromagnetic field modulation, representing a paradigm shift in neutron generation. Japan's J-PARC research center is advancing this technology to develop a high-flux neutron source with a target yield of 1 × 10^10^ n·cm⁻²·s⁻¹. Nevertheless, the intrinsic technological complexities associated with these systems currently restrict their deployment to large-scale research infrastructures, underscoring unresolved challenges in miniaturization and cost-effective scalability for clinical applications.

D-T/D-D neutron generators based on D-T or D-D fusion reactions (e.g., a generator series developed by the Budker Institute of Nuclear Physics in Russia), exhibited great promise in experimental trials for superficial tumor therapy owing to their compact design and neutron yields, which approached 1 × 10¹¹ n/s. However, the 14 MeV neutrons characteristic of D-T reactions require advanced moderation systems to optimize the neutron-energy spectrum for therapeutic applications owing to their intrinsic monoenergetic neutron-emission profile.

### 5.2 Target-material system

Lithium target technology is based on the principle of nuclear reactions, in which protons bombard a ^7^Li target to induce the ^7^Li(p, n)^7^Be reaction and generate neutrons within a low-energy range of 0.1-1.2 MeV. Thus, these neutrons are suitable for use with low-energy proton beams of up to 2.5 MeV 143. After moderation, these neutrons can be deployed for deep-seated tumor therapies. Initial implementations used solid lithium targets made of metallic lithium or lithium alloys. However, these targets melted under the excessive thermal load from proton beams. Modern advancements have led to the emergence of liquid lithium targets, as demonstrated by the circulating Li-F eutectic alloy developed by Japan's Kashiwagi Heavy Industries. This alloy extends target longevity to hundreds of hours through active flow cooling. Finland's Neuboron has further enhanced thermal management by integrating thin-layer liquid lithium with silicon carbide (SiC) substrates to improve heat-dissipation efficiency. Despite their compact dimensions, thermal management under high heat fluxes remains a critical technical constraint for these targets.

Beryllium target technology is also based on the principle of nuclear reactions, whereby protons bombard a beryllium-9 target to induce the ^9^Be(p,n)^9^B reaction and generate neutrons across an energy spectrum of 0.5-5 MeV. The resulting neutron flux exhibits significant medium-to-high-energy components, making it compatible with high-energy proton beams in the 8-30 MeV range.

However, robust spectral-shaping moderators are required to achieve the therapeutic energy spectrum. Although conventional beryllium targets utilized high-purity metallic beryllium plates, they suffered from thermal embrittlement and exhibited limited operational lifespans under intense beam irradiation. Modern iterations have addressed these limitations through engineered solutions: rotating beryllium targets now distribute the beam-induced thermal loads via rotational motion, thereby extending service lifetimes to approximately 200 h. Concurrently, beryllium-tungsten composite targets integrate tungsten substrates to enhance heat dissipation for sustained tolerance to power densities of up to 3 kW/cm². Although beryllium targets deliver exceptional neutron yields, the high-energy neutrons they generate in the MeV range pose critical challenges regarding the radiation-shielding efficacy and induced target-material activation. This necessitates advanced mitigation strategies for clinical implementation 143.

Beyond conventional lithium- and beryllium-based targets, advanced target systems that offer diverse solutions to neutron-generation challenges are emerging. High-temperature-resistant tantalum-lithium composite targets leverage a layered structure in which a tantalum base absorbs undesired thermal energy, and a lithium surface coating enables the neutron generation via ⁷Li(p, n)reactions. These targets are particularly well-suited to high-power accelerator operations. Concurrently, SiC-Li targets leverage the exceptional thermal conductivity of SiC as a heat-dissipating substrate that is paired with lithium thin films that produce neutrons. This design achieves prolonged operational longevity and stable neutron yields under sustained irradiation. In compact neutron-source applications, deuterated titanium targets exploit the ^2^H(d, n)^3^He reaction to generate 2.45 MeV neutrons via solid-state D-D fusion. This offers passive thermal management and a minimized system footprint, eliminating the necessity for liquid cooling infrastructure. However, the neutron yields are lower than those of conventional D-T systems. Although these innovations advance thermal resilience and system integration, they are limited by the issues of balancing neutron flux intensity, spectral control, and radiation safety across diverse operational regimes.

### 5.3 Beam-shaping assembly

BSA serves as the critical interface between the neutron source and patient, performing three principal functions: the spectral transformation of high-energy neutrons into therapeutically applicable epithermal ranges (0.5 eV-10 keV), the spatial confinement of the beam profile to match the tumor morphology, and the real-time optimization of dose-delivery parameters.

The main role of the moderator is to slow down high-energy neutrons (MeV class) to superthermal neutrons (keV-eV class) through inelastic scattering. Among the traditional materials employed as moderators, magnesium fluoride (MgF₂) exhibits a high moderating efficiency, although it requires a significant thickness of more than 30 cm to be effective. Additionally, heavy water reduces the neutron loss, although its large size limits its practical application. Thus, novel materials, such as lithium fluoride, simultaneously integrate neutron-slowing and -absorption functions to reduce γ-ray contamination. Additionally, a polyethylene (PE)/boron carbide composite layer exploited a structural gradient to fine-tune the neutron-energy spectrum. The innovation of a BNCT device by Kyoto University (Japan) lies in its rotatable slowing body structure, which dynamically regulates the neutron-energy-spectrum distribution through physical movement, thus opening up a new path for slowing technology. Currently, topology optimization algorithms and Monte-Carlo transfer codes can automatically adapt the design of the BNCT moderator to the specific characteristics of a patient's tumor and morphology, thus enabling more precise BNCT treatments 144.

To further enhance neutron-utilization efficiency, reflectors are used to redirect scattered neutrons into the beam channel. In the application of radiation-shielding materials, the characteristics of different materials must be comprehensively evaluated based on their operational contexts. Lead, as a high-density material, effectively reflects radiation, although it tends to generate secondary γ-rays under high-energy radiation impact, potentially causing secondary contamination. Tungsten exhibits superior radiation-reflection efficiency to lead, yet its shielding system must be integrated with a neutron-absorption layer to achieve comprehensive protection. Beryllium reflects neutrons and minimizes concomitant γ-ray contamination, which makes it more suitable for scenarios requiring precise shielding. Multi-layered composite structures, such as alternating lead and PE layers, are widely employed in practical engineering. By leveraging the synergistic effect of metallic and organic materials, these structures address the shielding requirements for diverse radiation types.

The collimator achieves precision radiotherapy in tumor-targeted regions by constraining the neutron-beam direction and the irradiation scope. Early-stage fixed collimators, constructed from lead/tungsten alloy materials, provided fundamental therapeutic functionality via fixed apertures. However, they were inadequate for complex tumor morphologies. The subsequent development of multi-leaf collimators enabled dynamic adaptability via the positional adjustments of metallic leaves. This hardware is now synergistically integrated with artificial intelligence (AI)-driven real-time optimization algorithms to refine irradiation-field geometries. This innovation substantially enhanced compatibility with biological dynamics, such as respiratory motion. Recent advancements in 3D-printed collimators transcended conventional manufacturing constraints, enabling the fabrication of customized complex-channel architectures with exceptional efficacy in the personalized treatment of irregular tumors, particularly in craniofacial regions. This advancement from rigid configurations to dynamically adjustable systems, and ultimately to customized designs, underscores the continuous technological evolution of collimators in advancing radiotherapy toward enhanced precision and reduced adverse effects.

The world's first and currently the only approved AB-BNCT system for clinical care was initially configured with only a planar collimator (or flat collimator, FC) on the patient side. However, FC struggled to bring the lesion site sufficiently close in the management of some patients with head and neck cancers, posing the risk of prolonged irradiation time and excessive exposure of normal tissues. To address this challenge, a new type of collimator with a raised profile (i.e., the extended collimator) was developed for patients with head and neck cancers. It received drug approval in February 2022. 145.

Neutron filters perform essential spectral shaping by selectively attenuating fast neutrons with energies of more than 10 keV and thermal neutrons with energies of less than 0.5 eV, thereby preserving therapeutically vital epithermal neutrons ranging from 0.5 eV to 10 keV. Bismuth serves as the primary material for fast-neutron filtration owing to its moderate atomic number, which allows it to effectively scatter high-energy neutrons while suppressing secondary γ-ray generation. Cadmium, characterized by its exceptionally high thermal-neutron-absorption cross-section (~2450 barns), precisely eliminates thermal-neutron background, although its thickness must be meticulously optimized through Monte-Carlo simulations to avoid the excessive attenuation of epithermal neutrons. Gadolinium provides a compact solution for mobile neutron-therapy systems by leveraging its ultrahigh thermal-neutron absorption capacity of approximately 49,000 barns in geometrically constrained configurations. Generally, advanced gradient filters utilize layered bismuth-aluminum architectures to synergistically integrate neutron-moderation and absorption mechanisms. This engineering innovation achieves a 98.5% attenuation rate for detrimental neutron components while maintaining the therapeutic beam flux at 1 × 10^9^ n/cm^2^/s, demonstrating an optimized balance between spectral purification and beam-intensity preservation.

Dose-monitoring systems have undergone continuous technological advancements for improved real-time performance and measurement accuracy. The traditional gold-foil activation method provides high precision, although it requires offline analysis, resulting in several hours of delays. Notably, although the subsequently developed boron trifluoride proportional counters enabled millisecond-level online monitoring, they remained susceptible to γ-ray interference. The next-generation semiconductor detector (SiC), by leveraging its radiation resistance and rapid response capability, suppresses interference errors to less than 3% in high-radiation environments while achieving continuous monitoring. Additionally, integrated imaging technology utilizing zinc sulfide (Ag) scintillators further visualizes dose distribution, and AI-powered deep learning models predict 3D dose profiles within half a second, with errors of less than 3%. These innovations, which span foundational measurement techniques to intelligent analytics, collectively establish a multidimensional real-time monitoring framework, thus driving radiotherapy into an era of precision and AI.

### 5.4 Clinical translation of boron neutron-capture therapy: Recent advances and future Directions

The clinical translation of BNCT has recorded multidimensional breakthroughs in recent years, with core advancements focusing on the optimization of therapeutic systems and the expansion of clinical implications. Supported by pivotal Phase II clinical trial (JHN002) data, Japan became the first country to integrate BNCT into its national health insurance system in May 2020, approving a boron-containing compound for the treatment of recurrent head and neck cancers. The trial demonstrated an overall objective response rate of 71%, with 2-year overall survival rates of 58% and 100% for recurrent and locally advanced patients, respectively. These results underscore the clinical efficacy of BNCT in treating refractory malignancies. Technologically, accelerator-based neutron sources have progressively replaced nuclear-reactor-based sources as the mainstream modality owing to their hospital compatibility and cost-effectiveness. Mature techniques for generating therapeutic neutron beams have been clinically implemented in countries such as Japan and Argentina. These techniques involve the proton bombardment of lithium/beryllium targets. This progress has been complemented in dosimetry by the introduction of the compound biological effect (CBE) concept that has revolutionized dose calculation. By integrating RBE with boron-distribution heterogeneity parameters, researchers established a CBE database. This database, tailored to diverse boron agents and tumor types, significantly enhances the accuracy of dose prediction. These systemic advancements propel BNCT from experimental exploration toward standardized clinical practice and provide a translational framework for extending its application to complex scenarios, such as glioblastoma and melanoma. Collectively, these developments mark the transition of BNCT into an era of precision and standardized cancer therapy 52.

### 5.5 Global development status and emerging frontiers of accelerator-based boron neutron-capture therapy Devices

#### 5.5.1 Accelerator-based boron neutron-capture therapy in Japan: Current status of the technological advancement

**Figure 27:**
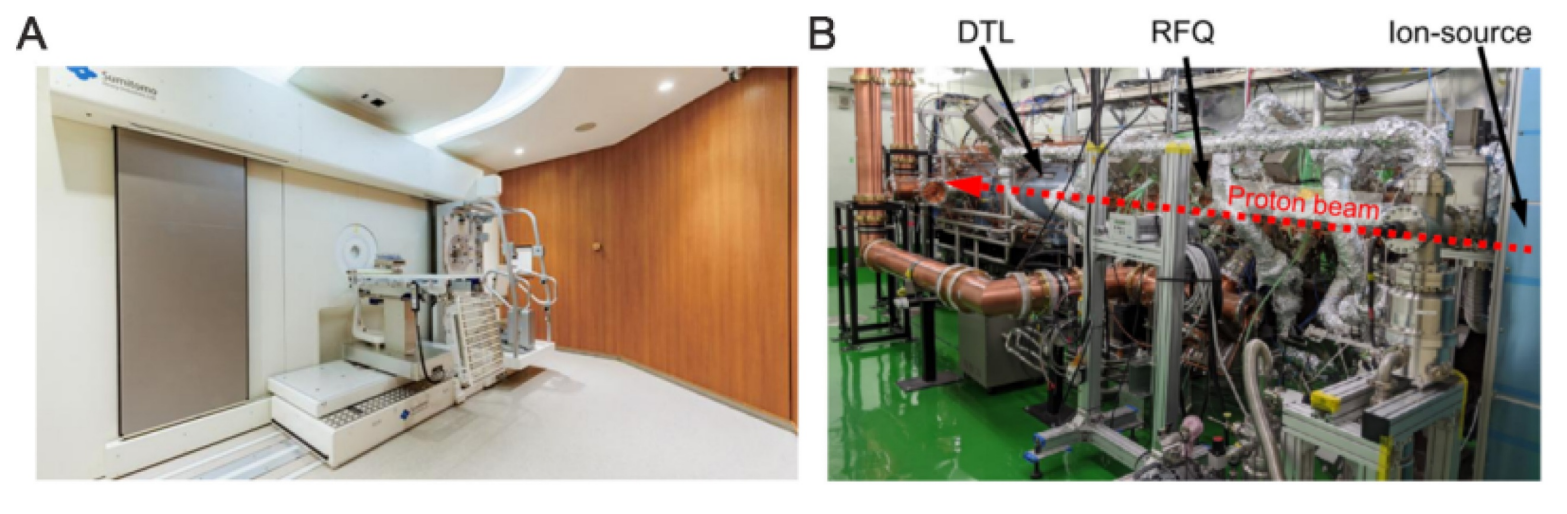
** (A)** Sumitomo's NeuCure® Therapeutic System. **(B)** RFQ+DTL Accelerator Configuration. Reproduced with permission from Ref. 148. Copyright 2023, Mary Ann Liebert, Inc.

Japan is the first country to apply AB-BNCT technology to clinical treatment, specifically for the treatment of head and neck tumors. Globally, the research and development (R&D) of BNCT equipment is expanding rapidly, with Japan taking the lead in clinical applications 146. Currently, three clinical institutions across Japan have operational BNCT systems. The first BNCT system developed in Japan was based on a cyclotron with a beryllium target. This system is installed at Kansai Medical Center and Nanto Hospital 147 and complies with hospital radiation safety standards, as well as innovatively integrates a dual positioning mechanism (sitting/lying), which significantly improves the targeting accuracy for recurrent head and neck cancers and malignant gliomas (Figure 27A). A significant innovation emerged from the collaboration between Tsukuba University Hospital and the High Energy Accelerator Research Organization (KEK). Their LINAC integrates RFQ and drift tube LINAC (DTL) technologies (Figure 27B), enabling the output of an 8 MeV proton beam with an average current of 2.1 mA within a compact structure. This clinically stable system currently supports a Phase II clinical trial for the treatment of newly diagnosed malignant gliomas. Another multimodal platform at Shonan Kamakura Hospital combines an intelligent patient-transfer system with proton therapy and BNCT to explore comprehensive cancer-treatment strategies. Notably, these institutions use a well-known boron agent, which features blood-boron-concentration control of more than 25 μg/g. This significantly enhances the feasibility of adjusting the treatment window. The clinical translation of BNCT technology in Japan is attributed to three key factors: (1) device miniaturization (volume reduction of more than 60%), (2) expanded indications (including head and neck cancers, gliomas, and melanoma), and (3) policy support (national health insurance coverage implemented since 2020). According to the Japanese Radiation Oncology Study Group statistical report, these advancements have benefited over 2000 patients, establishing a replicable model for global oncology applications 148.

#### 5.5.2 Accelerator-based boron neutron-capture therapy in Korea: Current status of technological advancement

**Figure 28:**
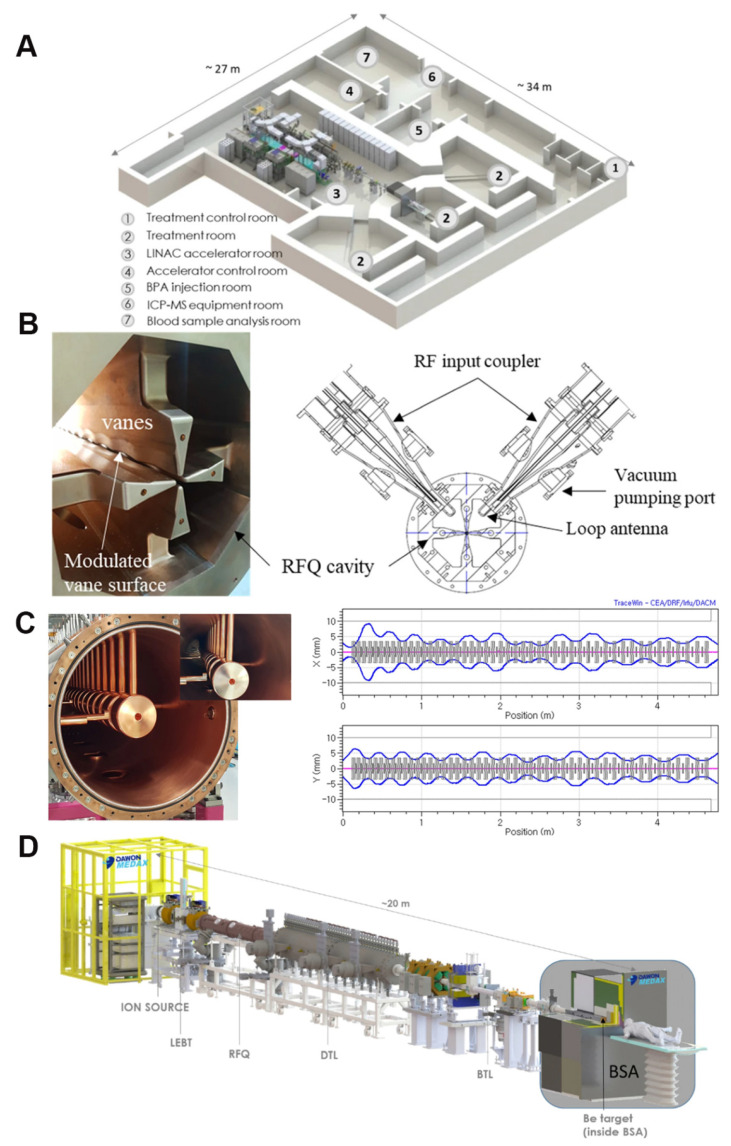
** (A)** DawonMedax AB-BNCT Facility Layout. **(B)** (Left) Photo of 4-vane RFQ and (right) cross-sectional view of RFQ and RF input coupler. **(C)** (Left) Photo of the inner structure of the DTL linac and (right) a beam trace simulation of the output beam from the RFQ into the DTL. **(D)** Layout of the 10-MeV proton linac and neutron generator with a beryllium target and BSA. Reproduced with permission from Ref. 149. Copyright. 2022, Springer.

Korea's first AB-BNCT facility is a prime example of a compact design, thanks to its LINAC-based architecture (Figure 28A). The system employs a 10 MeV proton beam with a beryllium target configuration that can generate a compliant surface neutron flux of ≥1 × 10⁹ n/cm²/s at an average current of 2 mA. This meets the threshold requirements recommended by the International Atomic Energy Agency. By innovatively integrating RFQ (Figure 28B) with DTL (Figure 28C), the footprint of this accelerator was significantly reduced, thereby enhancing its feasibility for hospital deployment. The durability of the target material was also enhanced by depositing a beryllium layer (thickness: 0.7 mm) on a vanadium substrate to effectively mitigate hydrogen embrittlement due to proton bombardment. The BSA (Figure 28D) integrates MgF_2_ moderators, aluminum/lead composite reflectors, and bismuth filters to effectively suppress fast neutron and γ-ray contamination. The most recent advancements in Korea's AB-BNCT follow a dual technical approach: high-energy optimization and precision enhancement. An ultra-compact cyclotron, equipped with a dual-frequency resonant cavity and gradient magnetic field optimization, has realized a breakthrough proton beam energy of 30 MeV, with a neutron yield of up to 1.5 × 10¹³ n/s/cm². Remarkably, the system volume was reduced to one-third of those of traditional devices, and a 35% cost-benefit optimization was achieved through a fully autonomous industrial supply chain. The LINAC system was developed in parallel, utilizing multi-stage beam control technology to achieve an adaptive neutron-energy-modulation device, thereby reducing the 3D dose-distribution deviation to the clinical threshold of 2 mm. The plug-and-play modular architecture also enabled treatment-room deployment within 72h. Both technological frameworks synergistically address clinical needs: the cyclotron system rapidly eliminates superficial tumors, and the LINAC platform enables the precise targeting of deep lesions. These innovations collectively mark a breakthrough in South Korea's field of nuclear medicine 149.

#### 5.5.3 Accelerator-based boron neutron-capture therapy in Argentina: Current status of technological advancement

**Figure 29:**
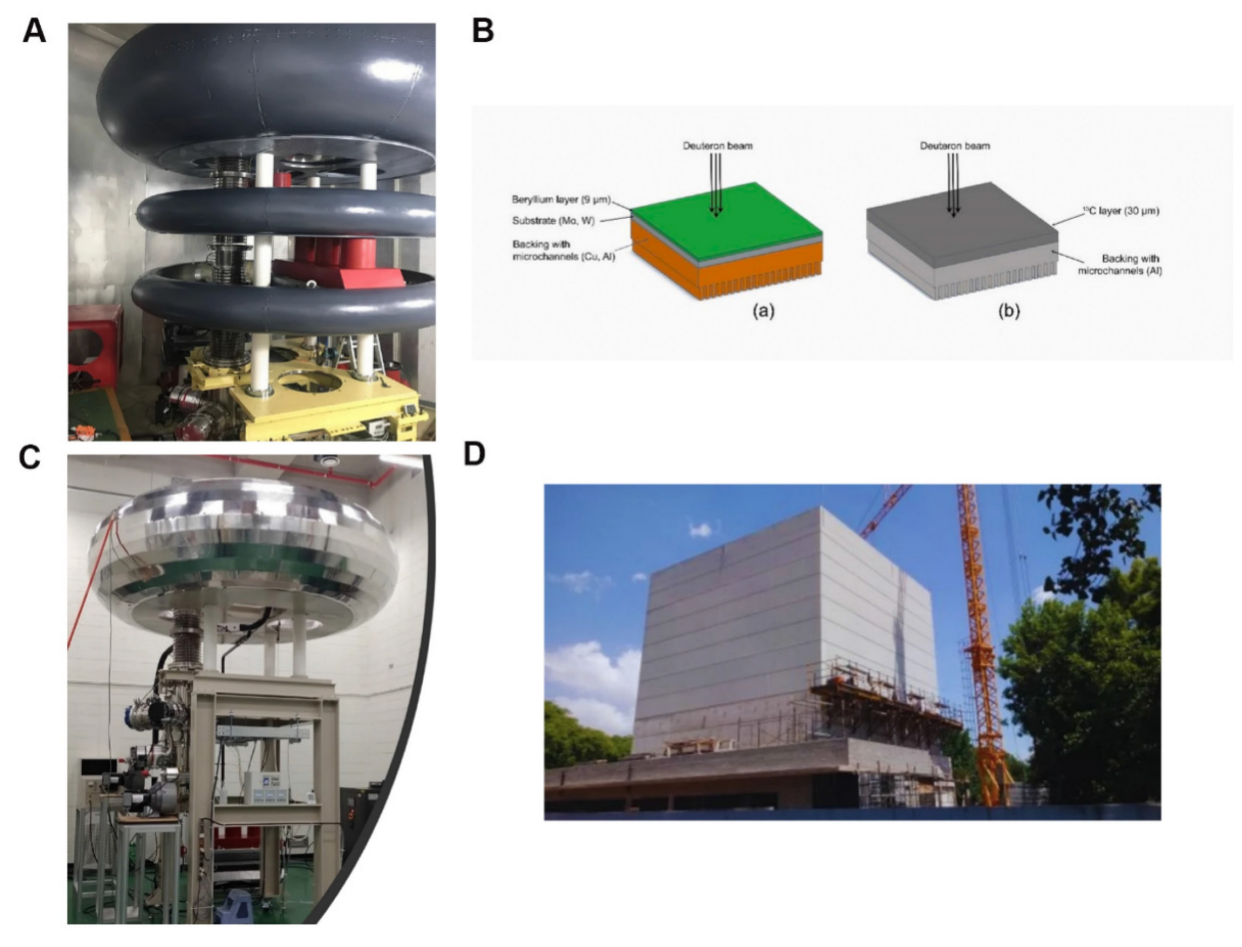
** (A)** Accelerator developed at CNEA (in air operation, no SF6). Reproduced with permission from Ref. 151. Copyright. 2024, Health Technol. **(B)** Beryllium and ¹³C Target Assemblies. Reproduced with permission from Ref. 150. Copyright. 2025, Elsevier. **(C)** Accelerator transferred and installed in KIRAMS-Korea. Reproduced with permission from Ref. 151. Copyright. 2024, Health Technol. **(D)** New lab and BNCT Centre under construction. Reproduced with permission from Ref. 151. Copyright. 2024, Health Technol.

Argentina is developing the world's only Southern Hemisphere AB-BNCT project, with its core being a 1.45 MeV ultra-low-energy ES quadrupole (ESQ) accelerator (Figure 29A). This project utilizes the world's lowest-energy 1.45 MeV ESQ accelerator that can drive ^9^Be(d, n) or ^13^C(d, n) reactions (Figure 29B) 150 to generate therapeutic neutrons through exothermic nuclear reactions produced by the bombardment of a ^13^C target by deuterium nuclei. It also incorporates an aluminum fluoride BSA device to achieve zero long-term radioactive activation. The core advantage of this technology is its extremely low generation of radioactive byproducts. For instance, continuous operation for 30 days generates only 0.78 GBq of tritium activity, which is a reduction of three orders of magnitude compared with mainstream proton schemes. This fully complies with hospital environment as-low-as-achievable safety standards. Regarding engineering implementation, the project has achieved key milestones: the modular prototype accelerator (0.72 MV) has completed system validation, demonstrating its technical feasibility. In 2022, the technology was transferred across borders to the KIRAMS research institute in South Korea (Figure 29C), providing an innovative tumor-treatment pathway for moderately developed countries (Figure 29D), thus combining operational cost-effectiveness with radiation-safety advantages to mark a paradigm shift in ultra-low-energy accelerator technology within the field of clinical radiation therapy 151.

#### 5.5.4 Accelerator-based boron neutron-capture therapy in Finland: Current status of technological advancement

**Figure 30:**
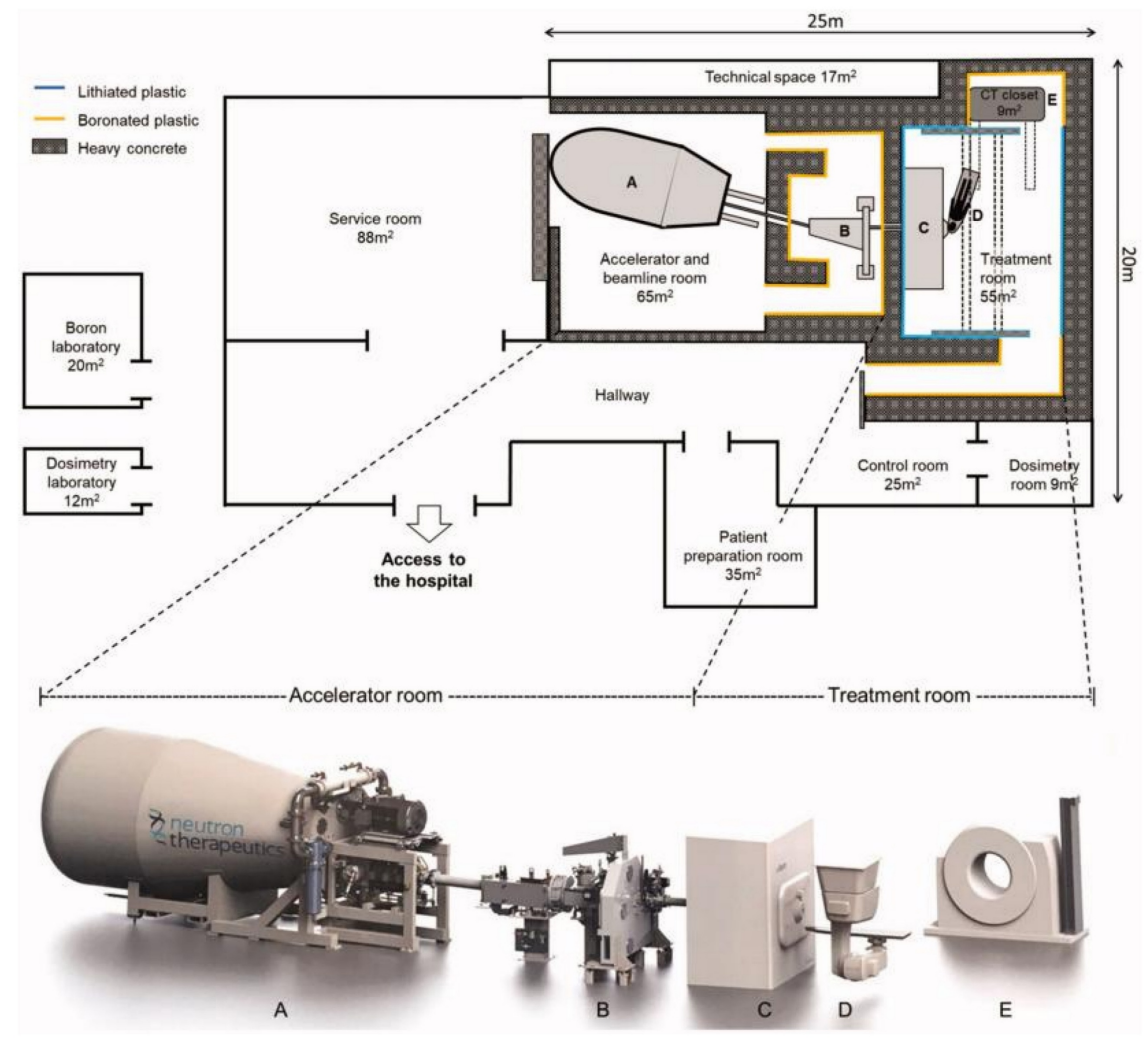
Helsinki University Hospital's nuBeam System. Reproduced with permission from Ref. 152. 2022, Acta Oncol. Found.

The AB-BNCT system at Helsinki University Hospital, Finland, uses the nuBeam technology platform (Figure 30). It features a 2.6 MeV ES proton accelerator that delivers a beam current of 30 mA. In this system, therapeutic neutron beams are generated via the proton bombardment of lithium targets, and the beam-transport system incorporates real-time proton-flux monitoring and a dynamic BSA. A six-degree-of-freedom robotic arm-assisted Exacure positioning system also ensures submillimeter irradiation accuracy. The treatment vault uses radiation shielding based on borated/lithiated polymers and heavy concrete. It is integrated with an on-rail CT scanner for image guidance. The dosimetric protocols combine neutron-activation analysis with paired ionization-chamber measurements, whereas treatment planning is based on GEANT4 Monte-Carlo simulations. Having secured regulatory approval for radiation safety, this facility is prioritizing clinical applications for recurrent head and neck cancers, marking Finland's entry into advanced BNCT-based cancer treatment 152. Additionally, European countries, such as Finland, Italy, Sweden, and the United Kingdom, are collaborating to promote the AB-BNCT program, which focuses on integrating the RFQ field with DTL technology 146.

#### 5.5.5 Accelerator-based boron neutron-capture therapy in China: Current status of technological advancement

**Figure 31:**
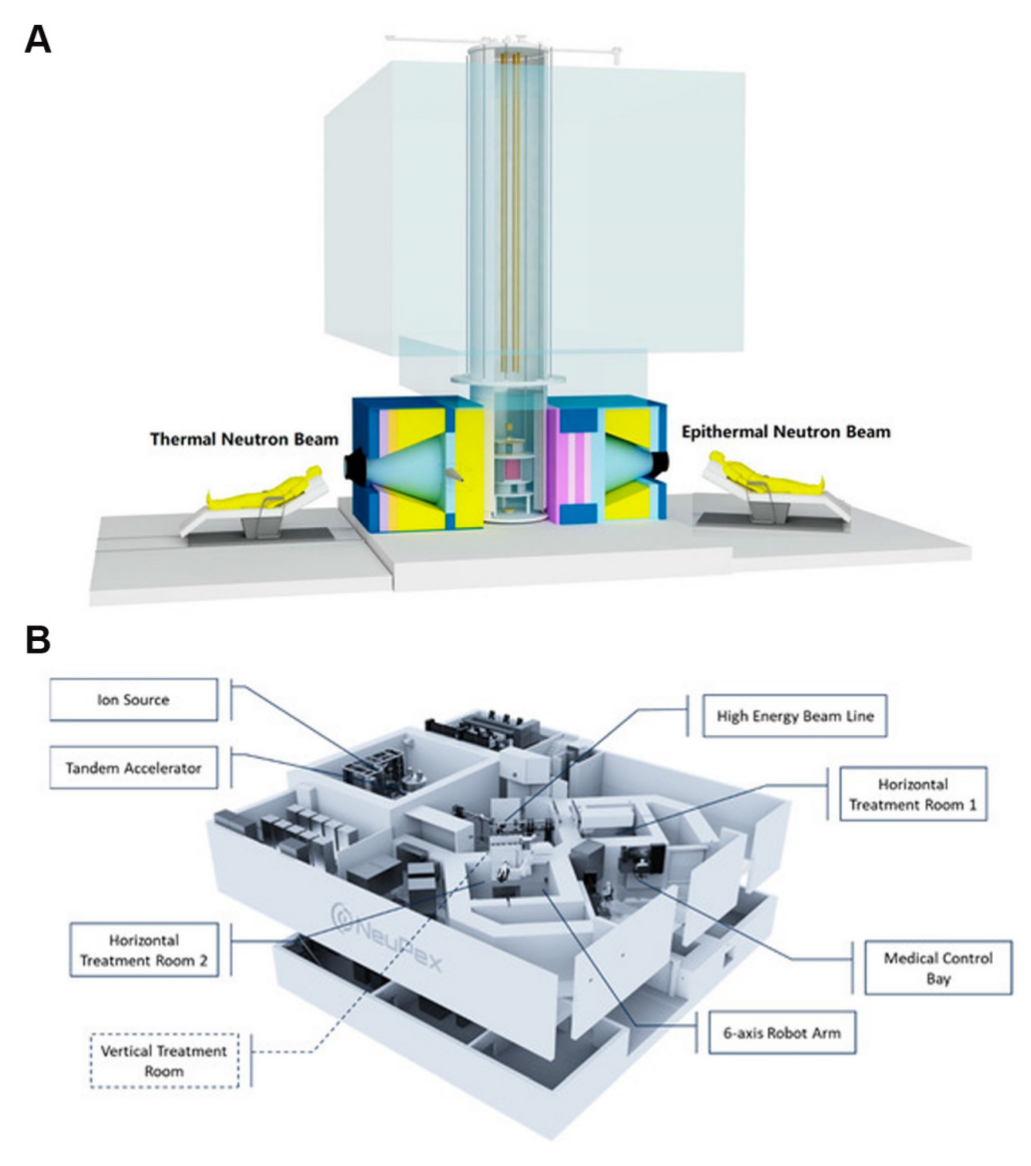
** (A)** IHNI-1 Miniature Reactor System. **(B)** NeuPex Accelerator-Based BNCT System. Reproduced with permission from Ref. 153. Copyright. 2023, MDPI.

The clinical research landscape and BNCT-facility development in mainland China encompasses reactor-based and accelerator-driven systems, which are currently in clinical trial phases with planned medical device registration. The reactor-based system represents the world's first hospital-integrated miniature-reactor neutron source. It operates at a thermal power of 30 kW with a rapid activation time (23 min) and produces zero nuclear waste, making it capable of treating superficial and deep-seated tumors (Figure 31A). The accelerator-driven system, which is China's inaugural AB-BNCT system, features six-axis robotic positioning and adjustable energy-spectrum modulation for depth-adaptive tumor targeting, demonstrating superior treatment efficiency (Figure 31B) 153. Initially, a model of this system was equipped with a TES accelerator produced by TAE Life Sciences. The design prototype was based on a Russian accelerator 154. China's advancement in accelerator technology has resulted in the construction of accelerator-based neutron sources of different technical types. For example, the devices developed by the Institute of High Energy Physics (IHEP) of the Chinese Academy of Sciences and Lanzhou University are based on RFQ LINACs. These devices are operational in different locations, with one of them being used for experimental research on BNCT-related technologies and another currently being installed and awaiting commissioning at a hospital 155. China's notable technological breakthroughs include the high-current proton LINAC developed by IHEP, Chinese Academy of Sciences. It incorporates an RFQ and DTL integrated configuration, achieves a proton energy of 8 MeV with a 2 mA beam current while reducing the physical footprint by 60% compared with conventional systems. It also includes Tsinghua University's all-in-one integrated accelerator that employs an electron gun, an RF system, and a target station co-design to compress treatment-vault dimensions to 40 m². Further, the superconducting cyclotron co-developed by Sun Yat-sen University Cancer Center and China General Nuclear Power Group is another breakthrough that emphasizes operational stability, delivering clinical-grade beam output with 300 h continuous operation reliability. These advancements collectively establish China's strategic positioning in the development of next-generation BNCT system, balancing technical innovation with clinical translation requirements.

#### 5.5.6 Accelerator-based boron neutron-capture therapy in other countries: Current status of technological advancement

Globally, many countries are actively advancing AB-BNCT R&D initiatives. Russia's Budker Institute has developed a vacuum-insulated tandem accelerator employing 2.0 MeV protons and lithium targets, and IPPE-Obninsk has engineered a neutron generator. Italy's INFN Legnaro is developing a high-current (30 mA) RFQ accelerator utilizing 4-5 MeV protons with beryllium targets. In parallel, Italy's National Center for Oncological Hadrontherapy is investigating ES accelerator configurations paired with lithium targets, reflecting diversified technological pathways in the development of accelerator-driven neutron sources 108.

### 5.6 Limitations and directions for the development of accelerator-based boron neutron-capture therapy

Currently, the focus of accelerator technology research is mainly on the development of high-current accelerators, the design of long-lifespan targets, and the optimization of neutron beams, which may further expand the clinical applications of AB-BNCT systems while addressing the dual requirements of equipment miniaturization and radiation safety 156.

Consequently, BNCT accelerator development faces three principal challenges. First involves immature dosimetric verification protocols and a lack of commercialized imaging systems. The second involves the conflicting design requirements for BSAs. Beryllium-based targets induce component activation under high-energy proton bombardment, whereas their lithium-based counterparts struggle with thermal management and radiotoxicity accumulation. The third encompasses insufficient clinical standardization, where convergent thermal-neutron distributions across technologies are undermined by spectral discrepancies, thereby hindering multicenter trial harmonization.

Future priorities for BNCT must converge on three key areas (1) enhancing beam directionality via the use of advanced collimators and superconducting technologies; (2) developing modular systems and standardized components to reduce capital costs; and (3) integrating neutron sources, boron pharmaceuticals, and imaging modalities to establish closed-loop therapeutic systems. With evolving international standards, BNCT is poised to transcend experimental limitations and emerge as a precision radiotherapy modality for refractory malignancies, such as head and neck cancers and gliomas. This breakthrough will be driven by the synergistic effect of advancements in physics, radiochemistry, and clinical oncology 29.

## 6. Conclusion and prospects

Currently, BNCT has transitioned from its early exploratory phase to a critical period of parallel clinical application and technological optimization. The evolution of boron drugs has progressed from the first generation (inorganic boric acid) through the second generation (BPA and BSH), and now to the third generation (boron-cluster compounds, e.g., carboranes). These newer compounds have significantly enhanced tumor-enrichment capabilities through advanced technologies, such as molecular-level targeting modifications and nano-delivery systems. In clinical applications, BNCT has demonstrated efficacy in treating gliomas, recurrent head and neck tumors, and melanomas. Notably, Japan's inclusion of BNCT in its national health insurance in 2020 marked a pivotal shift from “experimental therapy” to “conventional therapy.” However, further work, as highlighted below, is required to advance BNCT development:

1) **Optimization of third-generation boron-cluster compounds**: Future efforts will focus on enhancing tumor specificity and boron loading through molecular-level targeting modifications and the development of nano-delivery systems. Additionally, the development of “theranostic” boron drugs will enable the real-time monitoring of boron distribution, thus facilitating therapeutic synergy.

2) **Advancement of accelerator-based boron neutron-capture therapy**: Replacing conventional reactors with AB-BNCT systems will prioritize the optimization of the neutron-energy spectrum and the miniaturization of devices. This will reduce equipment costs and maintenance challenges, thereby facilitating the widespread adoption of AB-BNCT in hospitals.

3) **Expansion of the clinical applications of the technology**: While BNCT is primarily used for gliomas, melanomas, and recurrent head and neck tumors at the moment, future advancements in boron drugs and neutron sources must focus on expanding its application to deep-seated or metastatic tumors (e.g., liver, breast, and pancreatic cancers). By accumulating clinical data to validate its efficacy across diverse malignancies, efforts must be directed at promoting the integration of BNCT in national health insurance systems, thereby broadening its indication range.

4) **Development of advanced imaging technology**: High-sensitivity, high-resolution multimodal imaging technology is required to monitor the real-time dynamic distribution of boron drugs within tumors. Combined with popular AI algorithms, this technology will predict optimal treatment timing and optimize dose calculations through imaging, thereby enhancing treatment accuracy.

5)** Synergistic combination therapies**: Leveraging the complex DNA damage induced by the high LET particles of BNCT, combination therapies using repair-pathway blockers, such as PARP and DNA-PKcs inhibitors, must be explored to enhance the irreversibility of DNA damage, as well as investigate synergistic mechanisms with other therapies to develop multidimensional anticancer systems.

In summary, ongoing advancements in BNCT are poised to transform cancer-treatment paradigms, making BNCT a more effective and accessible treatment option. This is being achieved by optimizing boron-drug design, enhancing neutron-source technologies, expanding clinical applications, integrating advanced imaging, and exploring synergistic combination therapies. These efforts will improve therapeutic outcomes for patients with various malignancies and establish BNCT as a cornerstone of modern cancer therapy, ultimately benefiting a broader patient population.

## Figures and Tables

**Table 1:** Summary of Boron Cluster-Based Boron Delivery Agents

Boron delivery agents	Tumor-to-Normal ratio	Tumor-to-Blood ratio	Boron in tumor (μg/g)	Tumor or cell model	Ref.
BSH-3R-DOTA	15.5 8.2	2.8 1.9		U87ΔEGFR glioma	**54**
B139		5.88	50.7	SAS tumor-bearing model	**12**
cis-ABCPC	8 5		64±11 64±18	F98 glioma mode/ B16 melanoma model	**56**
trans-ACBC-BSH cis-ACBC-BSH				U251 cell A172 cell U81 cell C6 cell	**57**
ACBC-BSH	14.2 42.4	6.4 13.7	17.8±10.1	F98 glioma model	**58**
boronated hematoporphyrin analogs				V79 Chinese Hamster lung fibroblast cell line	**65**
porphyrin-cobaltacarborane conjugates				Human laryngeal cancer cell line HEp2	**67**
carboranyl-BODIPYs				hCMEC/D3 human brain endothelial cells	**70**
CuTCPH ZnTCPH			71±19 84±21	Mouse EMT-6 breast adenocarcinoma/ SCCVII squamous cell carcinoma in mice/ Human malignant glioma U373	**75**
^10^B-ZnB4Pc	4		0.66	B16F1 melanoma in C57BL/6 mice	**76**
derivative 1				C6 glioma cell line	
Borane anionic sodium salt encapsulated in liposomes	12 3.3 7.5 2.6		13.9 13.6 11.2 8.8	CT26 colon cancer cell model of BALB/c mice	**88**
PEGylated liposome				CT26 colon cancer cell model of BALB/c mice	**89**
TAC MAC		5.66	43.0	EMT6solid flank tumors in BALB/c mice	**90**
Cobalt dicarborane cholesterol derivative				MCF-7 MCF-10A	**91**
Boronsome	37	4	93.3	4T1 tumour-bearing BALB/c mice	**92**
DOX-CB@lipo-pDNA-iRGD				glioma	**93**
BSH-BPMO				OVCAR8	**95**
B-AuNPs	12.02		217.1±47.1	N87 gastric cancer xenografts	**96**
DSPE-BCOP-5T	6.94 25.20	1.63 7.46	55.24±2.13 84.93±2.68	4T1 tumor-bearing mice	**97**
